# Using nitric oxide (NO) for gas sterilization at room temperature: A novel sterilization modality

**DOI:** 10.1371/journal.pone.0355735

**Published:** 2026-08-21

**Authors:** Weilue He, Julie Osborne, Megan C. Frost

**Affiliations:** R&D Department of Sterile State Inc., Grand Rapids, Michigan, United State of America; Johnson & Johnson, UNITED STATES OF AMERICA

## Abstract

The success of terminal sterilization is a critical step for medical device manufacturing to guarantee device functionality and limit infections. The Food and Drug Administration (FDA) Established Category A approved sterilization methods include heat (steam or dry), radiation (gamma, X-ray, electron beam), ethylene oxide (EtO) gas, and vaporized hydrogen peroxide (VH_2_O_2_). Although EtO is the most commonly used method in the industry, its high toxicity has always been a major concern for human health, environmental impact, and medical device integrity. The developement of novel alternative technologies are actively encouraged by the FDA, aiming to provide similar scalable sterility assurance level (SAL) as EtO gas while reducing health and environmental issues. Nitric oxide (NO) gas was evaluated as the novel sterilant provided by Sterile State Inc. (Grand Rapids, MI) to achieve SAL of 10^−6^ at room temperature (RT) and regular atmospheric conditions (21% oxygen and ambient pressure). Commercially available biological indicators (BIs) designed for heat, EtO, and VH_2_O_2_, and widely used spore species, including *B. atrophaeus, B. subtilis* and *G. stearothermophilus*, were used to validate the NO sterilization system. All the species showed a Log-linear reduction pattern under NO sterilization according to ISO 11138−1 guidelines, and the *D*-values of these microorganisms were determined. NO’s ability to inactivate clinically relevant bacteria, *E. coli* and *S. aureus*, was also evaluated through *D*-values. The sterilization cycle was performed at RT, specifically at 21−23 °C, regular atmospheric pressure, and the NO dose potentially exposed to end-users was significantly less than National Institute for Occupational Safety and Health (NIOSH) defined human Immediately Dangerous to Life or Health (IDLH) level. For the first time, we proved the utility and effectiveness of NO as an agent to achieve sterilization at mild conditions using BI and the SAL of 10^−6^ standard. NO sterilization holds great potential to be used as a green alternative to traditional gaseous sterilant sterilization for medical device manufacturing and infection control.

## Introduction

According to the Food and Drug Administration (FDA) [1], sterilization is defined as “a validated process used to render a product free of all forms of viable microorganisms”. To validate a sterilization practice, a predetermined Sterility Assurance Level (SAL) using specific bioburdens (normally bacterial endospores) needs to be established. The validation process involves several key elements including using proper biological indicators (BIs), specifying sterilization parameters, defining SAL (a probability of a nonsterile device occurring, normally 1 in 1,000,000, i.e., 10^−6^), and challenge studies such as overkill half-cycle method.

The medical device industry is associated with a terminal sterilization market that is estimated to be nearly $17 billion globally [2]. Currently, the FDA has approved limited types of sterilization methods including steam, dry-heat, radiation, ethylene oxide (EtO) gas, vaporized hydrogen peroxide (VH_2_O_2_), and some newer options like chlorine dioxide gas (ClO_2_), vaporized peracetic acid (VPA), and nitrogen dioxide (NO_2_). EtO accounts for approximately 50% of the medical device sterilization market because of its high materials compatibility with devices that are temperature, steam, and radiation sensitive or have complicated geometries such as catheters and combination products [3]. This large market share persists despite the high toxicity and carcinogenic nature of EtO gas. EtO sterilization operators can develop serious health issues such as irritations, nervous system damage, hematologic change, increased risk of spontaneous abortion and cancer [4]. EtO residue absorbed by medical devices is another important concern for patients and end users. Additionally, EtO can damage sensors and drug loaded devices. All of these risk factors underscore that an alternative sterilization method is urgently needed, especially for innovative devices. In fact, the FDA has been actively promoting alternative sterilization methods to reduce reliance on EtO since 2019, including the Sterilization Master File Pilot Programs. Most recently, VH_2_O_2_ was accepted as an Established Category A method in hopes of stabilizing the medical device supply chain and reducing EtO related air pollution. While effective, VH_2_O_2_ sterilization has its problems such as need for precise control of physical parameters, low penetration, limited compatibility with many cellulose-based medical device packaging materials, and residues that can be a risk to both workers and end users.

Sterile State Inc. aims to meet the need for an effective, safe, and widely compatible sterilization method through the use of nitric oxide (NO). NO is a highly reactive and diffusive free radical gas that is generated constitutively and inducibly in the human body and plays critical roles in the regulations of a wide variety of physiological processes. It is a potent inhibitor of platelet adhesion and aggregation [5,6], inhibits bacterial adhesion and proliferation [7], is implicated in mediating the inflammatory response toward implanted devices [8], inhibits smooth muscle cell growth and proliferation [9], and is a neurotransmitter [10]. NO is also the natural antimicrobial agent that allows the immune system in the human body to eliminate pathogens. NO exerts its antimicrobial functions via multiple reliable modes of action. It can immediately inhibit bacterial adhesion to surfaces by altering cell membrane adhesion proteins that mediate cell-substrate interactions [11,12]. NO destroys bacteria by puncturing and perforating the cell wall of bacteria through lipid peroxidation [11,12]. In addition, it can damage bacterial DNA beyond repair, modify proteins with reactive nitrogen species, and inhibit crucial metalloproteins that are part of the bacterial respiratory reactions [11,12]. This multi-modal antimicrobial property is important to ensure that there is no loophole for microbes to escape NO’s action and no chance to develop NO resistance. Another advantageous property of NO is that under ambient conditions (21% oxygen O_2_ and 1 atm pressure) it is consumed rapidly by O_2_ with a half-life reported to be in the second range. All of these properties, presence in natural, biological systems, multi-modal action, and short half-life when exposed to room air, makes NO an ideal safe and effective sterilant.

Previously, NO has been incorporated into various materials for anti-bacterial use and infection control [13,14]. However, the application of NO gas for sterilization has not yet been reported. In this study, we used a sustained NO delivery system developed by Sterile State Inc. (Grand Rapids, MI) to perform room temperature (RT) NO sterilization. The success of the sterilization cycle was validated by various commercially available biological indicators (BIs). Here we report, for the first time, the use of gaseous NO to meet sterilization standards at ambient temperature and pressure. This has important implications for compatibility of this sterilization method with sophisticated medical devices that are fragile or have sensitive bioactive properties.

## Materials and methods

### Reagents

Sterile Solution® 0.2 M from Sterile State Inc. was used as the sustained NO source. Sterile Solution® was prepared freshly according to IFU right before each sterilization cycle.

### Biological indicators

To evaluate the sporicidal efficacy of NO sterilization, a series of commercial BIs meeting the minimum performance criteria of ISO 11138−1 were utilized. The commercial indicators evaluated included: *Bacillus subtilis* (*B. subtilis* or *B. sub*) paper strips (Crosstex, REF: BS52306, LOT: B012, population: 2.0 × 10^6^ CFU/carrier), *Bacillus atrophaeus* (*B. atrophaeus* or *B. atro*) 6 mm paper discs (Crosstex, REF: BG-106D, LOT: ND105, population: 3.5 × 10^6^ CFU/carrier), *Geobacillus stearothermophilus* (*G. stearothermophilus* or *G. stearo*) 3 mm paper discs (Crosstex, REF: DS18−06, LOT: S2200502, population: 1.8 × 10^6^ CFU/carrier), and Apex® Stainless Steel Discs inoculated with *B. atrophaeus* (Mesa Labs, REF: GRS-090, LOT: AG-041, population: 2.9 × 10^6^ CFU/carrier). All commercial BIs were stored, handled, and maintained in strict accordance with the manufacturers’ specifications, using resealable airtight plastic storage bags supplemented with desiccant pouches at 2–8 °C to preserve spore dormancy. For customized BIs, commercial spore suspensions of *B. subtilis* 5230 (Mesa Labs, REF: SS5230E/8, LOT: SS5230−491, stock: 1.9 × 10^8^ CFU/0.1 mL) and *G. stearothermophilus* 7953 (Mesa Labs, REF: SSSE/7, LOT: SSS-981, stock: 2.3 × 10^7^ CFU/0.1 mL) were used following Mesa Labs’ Technical Report, *Product Inoculation*. Working suspensions were prepared by performing a 10-fold dilution in 40% isopropanol to achieve a target density profile in the ~ 10^7^ CFU/0.1 mL range. A 15 μL aliquot of the prepared suspension was then aseptically inoculated onto the bottom of a 4 mm high / 4 mm diameter medical grade polypropylene cup as the substrate. Following ambient air-drying in the biosafety cabinet for 6 h, these custom carriers were stored under refrigerated desiccant conditions (2–8 °C) for no more than 2 weeks before use. Prior to experimental deployment, the baseline viable spore populations of all types of BIs were verified via mechanical elution, serial ten-fold dilution (shown in Table 1), and agar plate enumeration in triplicate to confirm compliance with manufacturer-stated specifications.

**Table 1:** Illustration of different dilution preparations.

Dilution ratio	PBS in Vial I	V from Vial I transferred to Vial II	PBS in Vial II	V for inoculation	Extra PBS for inoculation
1:10000	1 mL	10 μL	990 μL	10 μL	90 μL
1:1000	1 mL	10 μL	900 μL	100 μL	0 μL
1:100	1 mL	N/A	N/A	10 μL	90 μL
1:10	1 mL	N/A	N/A	100 μL	0 μL
N.D.	100 μL	N/A	N/A	100 μL	0 μL

N.D. represents no dilution.

### Bacteria strains

Bacteria were purchased from Microbiologics (St. Cloud MN 56303) as *Escherichia coli* (*E. coli*) pellets (LOT: 483-1284-1, derived from ATCC©: 8739^TM^, REF: 0483E7, population: 5.4 × 10^7^ CFU per pellet) and *Staphylococcus aureus* (*S. aureus*) pellets (LOT: 485-1190-1, derived from ATCC©: 6538^TM^, REF: 0485E7, population: 5.7x10^7^ CFU per pellet). The bacterial samples were stored at 2–8 °C until use. To prepare the working bacterial suspensions, isolated colonies from fresh agar cultures were harvested and suspended in the phosphate-buffered saline (PBS). To ensure a homogenous suspension free of microbial clumping, the mixture was thoroughly disrupted using a vortex mixer. The initial population density was standardized photometrically by measuring the optical density at 600 nm (using OD600 of 1.0 corresponding to approximately 5 × 10^8^ CFU/mL as the rough reference). In accordance with USP < 61 > , the exact viable concentration of this baseline inoculum was verified via ten-fold serial dilutions in PBS, followed by spread-plating onto Mueller–Hinton agar before each experiment. The plates were incubated at 35 °C for 12 h or 20 h (for *E. coli* and *S. aureus*, respectively) to confirm the starting population via direct CFU counting. A standardized volume of the bacterial suspension (normally 2 μL in our study) was aseptically inoculated onto the interior surface close to the bottom of the 1.8 mL sterile glass sample vials. These prepared sample vials were then immediately subjected to the varying NO sterilization cycles for kinetic evaluation.

### NO sterilization unit architecture and parameter monitoring

**Fig 1:**
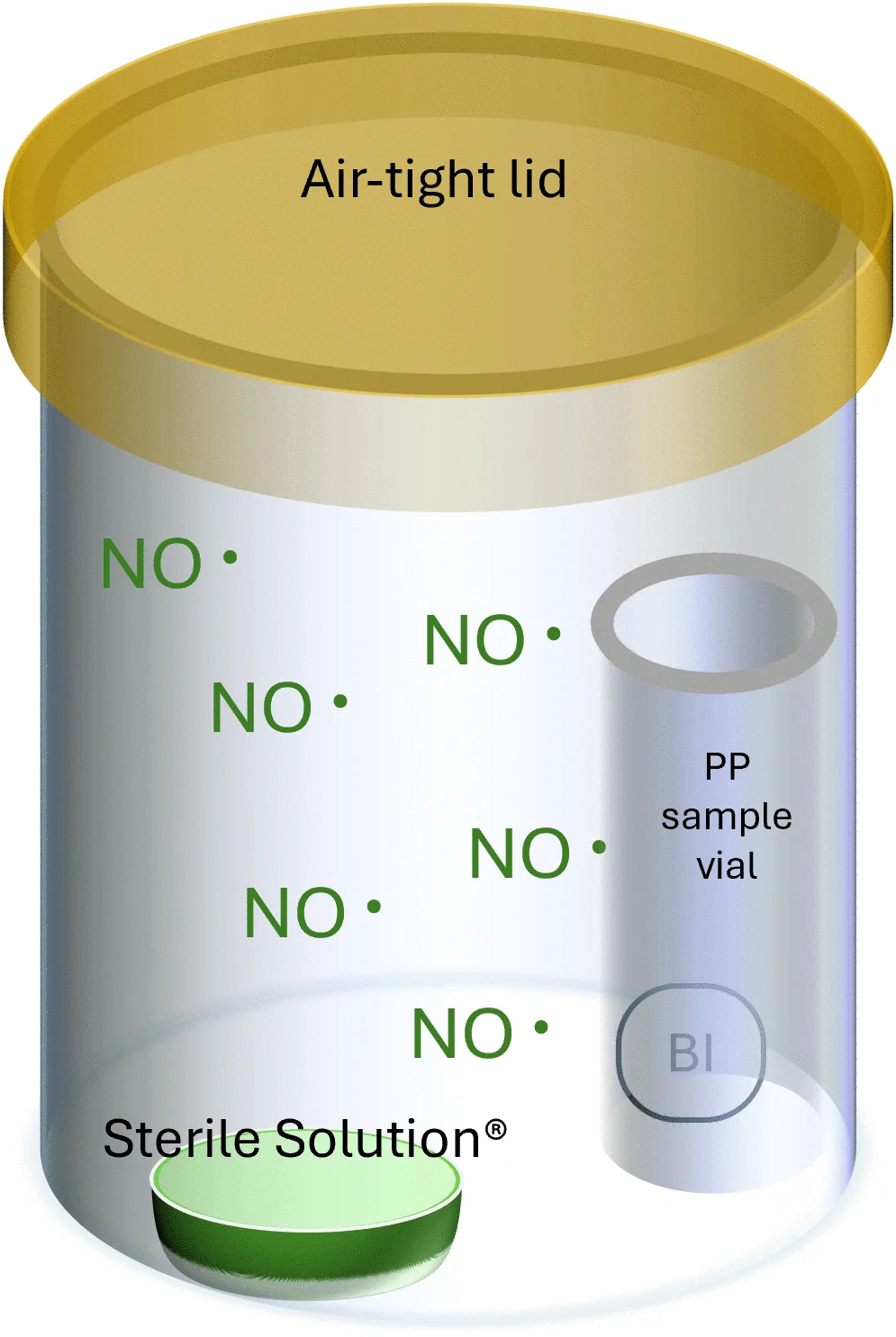
A representative setup of the Sterile State™ NO sterilization device. A polypropylene (PP) sample vial (the target object to be sterilized) containing a spore-inoculated commercial BI was loaded into the airtight 8 oz sterilization device. NO was delivered via 2 mL freshly prepared 0.2 M Sterile Solution®. The container was securely sealed, allowing the internal NO concentration to reach above 100 PPM. The sterilization cycle was conducted at RT. Different sterilization durations were achieved by controlling the time when to open the vessel.

NO generation and the sterilization cycles were conducted within individual, completely sealed vessel possessing an internal enclosure volume of 8 oz (or 236 mL), see Fig 1 as an example. NO was delivered *in situ* via the addition of a 0.2 M Sterile Solution® formulation. Specifically, a precise aliquot of 2000 μL of freshly prepared 0.2 M Sterile Solution® was dispensed into a 1-inch diameter containment tray situated at the base of the unit. A representative challenge load, a 5 mL polypropylene (PP) target vial as an example, with BI placed inside the sample vial, were positioned within the vessel to be sterilized. When evaluating multiple enveloped BIs, BIs were distributed well to prevent physical overlapping or stacking artifacts, thereby ensuring unhindered, uniform gas-phase mass transfer to all inoculation sites. After component loading, the chamber was sealed to establish an airtight environment, and the kinetic exposure period was initiated immediately upon closure. All sterilization cycles were conducted at ambient RT (defined as 18–23 °C in this study). To ensure environmental validation, profiles were continuously monitored and recorded per min using calibrated Govee™ digital sensors positioned both adjacent to and inside the active sterilization units.

### Qualitative evaluation of BI sterility via direct plating

To assess the binary (growth/no-growth) endpoint of the BI inactivation, i.e., to verify complete microbial inactivation, a direct plating method was employed. This approach was selected in accordance with the microbial recovery and neutralization principles outlined in USP < 1227> and ISO 11737−1 to mitigate potential sterilant carryover effects. Specifically, after exposure to the designated NO sterilization cycles, the treated BI carriers were aseptically retrieved and transferred directly onto the surface of Mueller–Hinton agar plates (Hardy Diagnostics). The plates were inverted and incubated under humidified conditions at either 35 °C (for *B. atrophaeus* and *B. subtilis BIs*) for 3 days then to 50 °C (for *G. stearothermophilus* BI) for 3 days. Qualitative evaluation was performed via visual inspection. Complete absence of any localized germination or macroscopically visible colony formation surrounding or originating from the carrier substrate after the incubation period indicates no-growth, while microbial growth is characterized by the proliferation of distinct colonies emerging directly from the carrier. All plates exhibiting no initial growth were maintained in the incubator for an extended validation period of no less than 3 days.

### Bacterial cultivation and surviving population determination

To evaluate microbial inactivation kinetics across distinct exposure durations, multiple independent NO sterilization units were set up for different end points. The initial baseline population (*N*_0_) was determined at *t* = 0. For spore BIs, samples were retrieved sequentially every 2 h, whereas vegetative bacterial carriers were sampled at 30 or 60 min intervals. For each designated exposure time point, a minimum of three independent replicates (n = 3) were analyzed. Following exposure, treated BIs were aseptically retrieved and subjected to quantitative evaluation in accordance with the recovery and methodological validation principles of ISO 11737−1 and USP < 61 > / EP 2.6.12 for quantitative enumeration. More specifically, for viable count determination, treated BIs were transferred into 1 mL of PBS buffer and mechanically agitated to optimize microbial detachment, adhering to the extraction guidelines of ISO 11737−1. Serial ten-fold dilutions were performed in PBS to achieve an ideal target density of approximately 30–300 CFUs on the plate, which is to meet the statistical accuracy requirements of USP < 61 > . For those samples where the surviving population was expected to be lower than the 10^2^ CFU level, no dilution was performed. The whole suspension was plated directly, and the corresponding BI carrier was also plated in the agar matrix to maximize the recovery efficiency.

### Calculation of *D*-value

Based on guidelines specified in ISO 11138−1 (Annex C) and ISO 14161, microbial inactivation kinetics were analyzed by constructing semi-logarithmic survivor curves. The *Log*_*10*_ of the surviving fraction (*Log*_*10*_*N*_*t*_) was plotted as a function of exposure time *t*. The *D*-value, defined as the exposure time required to reduce the microbial population by 90% (one *Log*_*10*_ cycle), was determined using the standard equation: *D*-value = *t*/(*Log*_*10*_*N*_*0*_ - *Log*_*10*_*N*_*t*_), where: *t* is the exposure time; *N*_*0*_ is the initial viable population when *t* = 0; *N*_*t*_ is the surviving population at time of exposure. The *D*-value was derived as the negative reciprocal of the slope of the linear regression line fitted to the survivor plot data. In alignment with ISO 11138−1, a coefficient of determination (R^2^ ≥ 0.8) was established as the minimum threshold for linear conformity. Potential deviations from linearity were evaluated using the contextual guidance provided in ISO 14161. To achieve a theoretical SAL of 10^−6^ from a starting population (normally over 10^6^ spores), the total required exposure time was calculated using: Time to SAL of 10^−6^ = (*Log*_*10*_*N*_*0*_ – (−6)) × *D*-value.

### NO level measurement

Quantification of NO level was performed using a Sievers Chemiluminescence Nitric Oxide Analyzer (NOA 280i; GE Analytical Instruments) [15,16]. The instrument operates based on a gas-phase chemiluminescent reaction between NO and ozone, emitting light that is detected by a cooled photomultiplier tube to generate a proportional electrical signal. Prior to analysis, a two-point calibration protocol was executed by a zero-gas baseline calibration using a filtered sweep gas and followed by a span calibration using a certified standard reference gas containing 45 PPM of NO (GE HealthCare). To monitor internal concentrations during processing, the NOA sampling line was coupled to the sterilization chambers via a customized, small-gauge needle interface. Real-time data acquisition was recorded at a frequency of 1 Hz (PPM per second) and archived in raw, unmodifiable formats for trace validation. Each independent sterilization unit was sampled for a fixed duration of 30 s, followed by a 2-min purging interval between consecutive units to clear the sampling line and prevent cross-contamination. The representative NO level for each experimental run was determined by averaging the stable concentration profile obtained during the initial 10 s data integration window. Our internal reference threshold for effective NO sterilization was established at 100 PPM. We designate this level as the empirical sterilization unit (artificial unit or a.u.). This benchmark was selected based on our observations that NO exhibits a high sporicidal efficiency against resilient bacterial endospores around this concentration without causing discoloration of the target. Consequently, the real-time internal concentrations quantified by the NOA 280i were continuously evaluated and reported against this reference baseline.

### Data and statistical analysis

Data points were reported as mean ± SD. For each time point, a sample size ≥ 3 was used. *D*-value calculation, linear regression, and trendline were calculated using MS Excel.

## Results and discussion

### Validation of physical parameters

**Fig 2:**
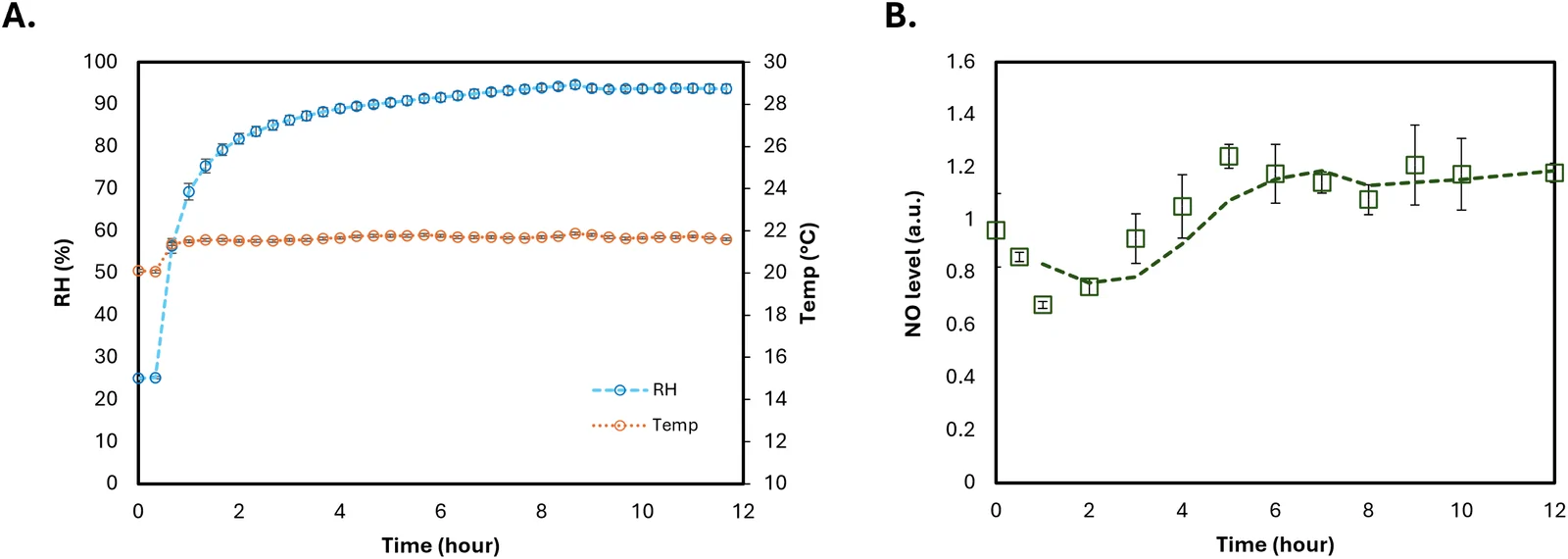
Physical parameter tracking during NO sterilization cycle. **A.**) RH and temperature over the course of sterilization cycle (mean ± SD); **B.**) Normalized NO levels over the course of the 12-h sterilization cycle.

The physical conditions were monitored and recorded over the course of the NO sterilization cycle using temperature and humidity sensors. All the raw data was shown in Supporting information S. Table 1 in S1 File. Fig 2A shows the average RT used in this study was around 22.3 °C (varying from 21.0–22.7 °C). The RH inside the sterilization device increased rapidly at the beginning stage, followed by leveling off, resulting in a plateau curve where the final RH was around 93%. NO levels inside the device were also monitored during the sterilization cycle using chemiluminescent analyzer. The measured NO levels in PPM are shown in Supporting information S. Table 2 in S1 File, while the normalized values are shown in Fig 2B, confirming that the NO sterilization device held the NO level close to the recommended range across the entire sterilization cycle. The immediately dangerous to life and health (IDLH) level recommended by National Institute for Occupational Safety and Health (NIOSH) was 100 PPM for exposures of 30–60 min [17]. While the targeted internal concentration within the sealed sterilization vessel can exceed this level for sporicidal applications, operator safety is ensured through strict engineering controls. The air-tight sealed unit prevents gas migration into the ambient environment during the exposure cycle. Furthermore, after the cycle, the micro-volume of gas released undergoes immediate, exponential volumetric dilution into the ambient room air, and NO’s short half-life allows rapid oxidation of NO into benign secondary products under standard atmospheric conditions [18].Like other chemical sterilization methods, sterilization efficacy of gaseous NO depends on concentration, humidity, and exposure duration. However, NO’s highly reactive with an extremely short half-life of several seconds under oxygenated conditions [19]. Our system uses a continuous NO generation source Sterile Solution®, a specialized formulation of S-nitrosothiol solution, that can provide sustained NO generation for days [20]. According to established kinetic modeling of NO concentration profiles based on equilibrium of generation and consumption of NO through oxidation [21], this constant flux allows us to achieve a steady-state where the internal NO concentration can reach a predictable and sustained plateau for sterilization applications [22]. In addition, due to the aqueous nature of Sterile Solution®, the internal RH concurrently reaches a stable equilibrium simply determined by the fixed liquid to headspace volume ratio (confirmed by Fig 2A). Therefore, this controlled configuration transforms a highly unstable gas into a controlled sterilization environment capable of inactivating microbial reliably.

### Confirmation of sterility using commercial BIs

**Fig 3:**
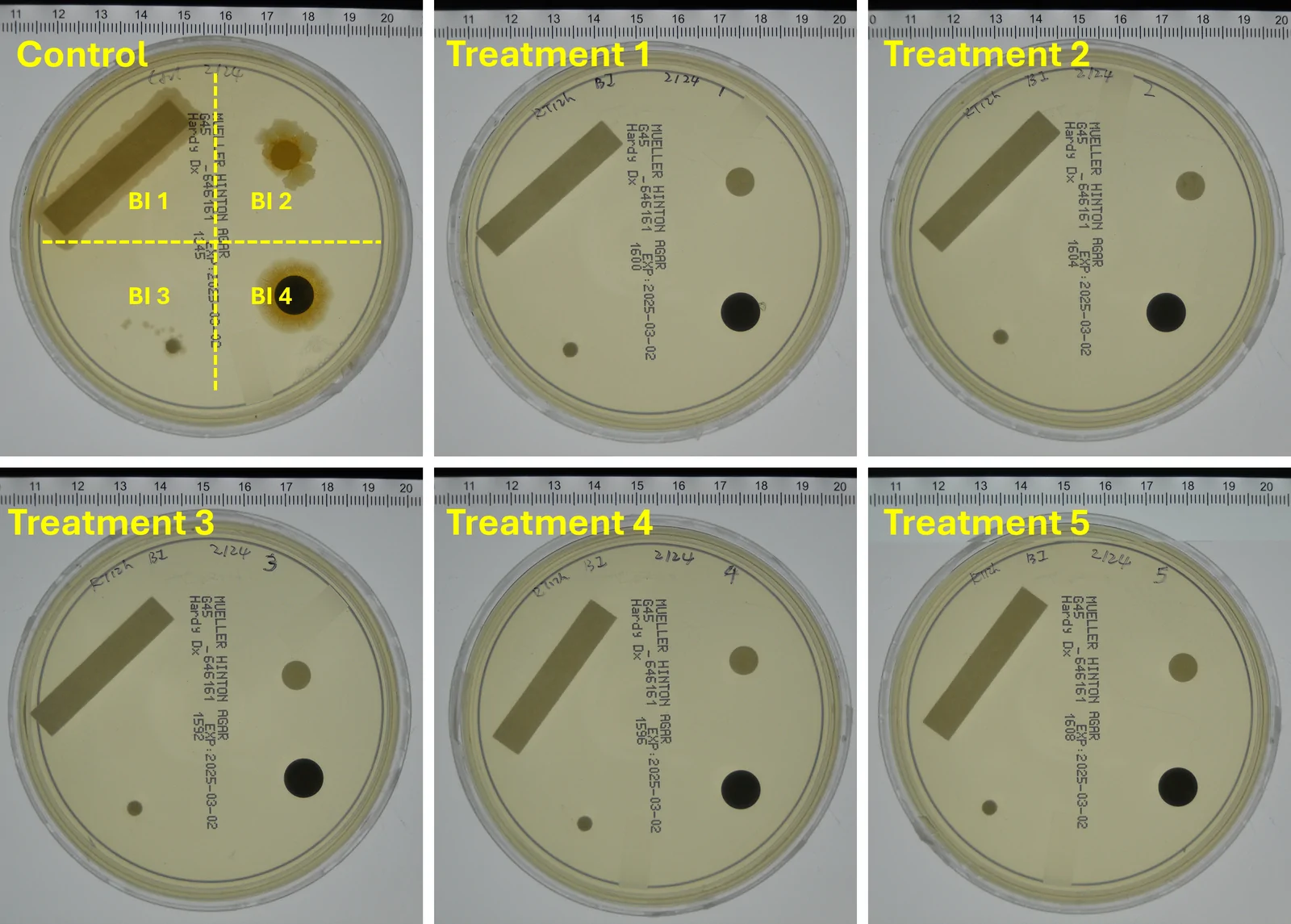
Direct plating culture method to confirm BI sterility after 12-h RT NO sterilization cycle using four different types of commercially available BIs (n = 5 for each). BI 1: Crosstex *B. subtilis* BI, BI 2: Crosstex *B. atrophaeus* 9372 BI, BI 3: Crosstex *G. stearothermophilus* BI, and BI 4: Apex® Stainless Steel Disc *B. atrophaeus* 9372 BI. Treatment conditions: BIs of each type were loaded inside a 16 oz vessel without overlapping. NO was delivered through 2 mL of 0.2 M Sterile Solution^®^. Then the vessel was tightly capped to form an air-tight completely closed sterilization unit. After the sterilization cycle at RT for 12 h, the BIs were immediately collected and placed on agar plates for culture. In the positive control plates, all BIs grew after culture (after overnight culture), while all treated BIs in different repeats showed no bacterial growth (after over 3-day culture).

Four different types of commercially available BIs, including: Crosstex *B. subtilis* BI (designed for low temperature steam, < 121 °C), Crosstex *B. atrophaeus* BI (designed for EtO and dry heat sterilization), Crosstex *G. stearothermophilus* BI (designed for steam sterilization), and Apex® Stainless Steel Disc *B. atrophaeus* BI (designed for VH_2_O_2_ sterilization), were used to test the efficacy of NO sterilization cycle at RT for 12 h, using n = 5 for each BI type. None of the BIs exposed to NO sterilization cycles had bacterial regrowth after over 3-days of culturing, while control BIs demonstrated clear regrowth just after overnight culture (Fig 3).

Note that these four BIs represent three most used bacterial endospores, two different spore carrier materials, and four different carrier material dimensions. They also represent validation of heat (steam and dry), EtO, and VH_2_O_2_ sterilization. All individual BIs contain over 10^6^ CFU, indicating at least Log 6 reduction of spores. Our results show NO’s sterilization efficacy is independent of BI type or carrier materials, underscoring the potency of the NO sterilization method. In practice, the standard NO sterilization cycle developed by Sterile State^TM^ Inc. is 24-h. For this study, the use of a 12-h sterilization cycle was chosen for validation of the overkill method. Since it is not practical to prepare BIs with a population of 10^12^ CFU to directly demonstrate the spore Log reduction (SLR) of 12, the overkill method relies on the half cycle using a Log 6 population BI under the same conditions as the full cycle and using the demonstrated Log linear reduction curves. Thus, when a Log 6 population BI for half cycle achieves sterility, theoretically, over 12 SLR of the same bioburden can be claimed by doubling the time.

### Spore *D*-value calculation

**Fig 4:**
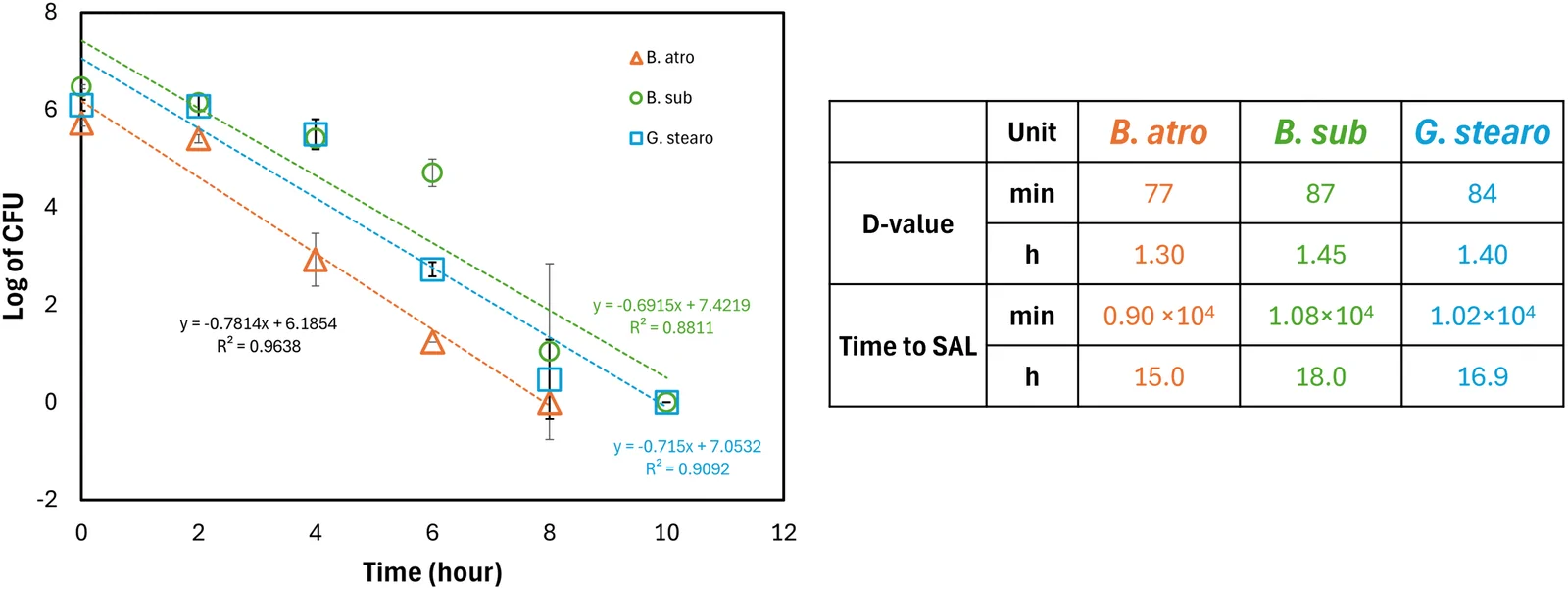
Calculation of *D*-values of three different types of commercially available BI on different carriers (n ≥ 3 for each type at each time point) undergoing RT NO sterilization cycle. BIs used were Apex® Stainless Steel Disc BI, *B. atrophaeus* Mesa Lab GRS-090, Mesa Lab SS5230E/8 *B. subtilis* 5230 35021, and Mesa Lab SSSE/7 *G. stearothermophilus* 7953. Log of remaining CFU over time (mean ± SD) and kill curves are shown in the plot and the calculated *D*-values are summarized in the table.

To compare kill kinetics for different endospores during NO sterilization at RT, we exposed the three most common BI spores, *B. atrophaeus*, *B. subtilis*, and *G. stearothermophilus* to the same RT NO sterilization cycle and examined the survival CFU over treatment time. The plate counting results with the corresponding dilution ratio are reported in Supporting information (S Table 3–5 in S1 File). The remaining CFU was calculated and expressed as Log numbers. In accordance with ISO 11138−1 guidelines, we evaluated the goodness-of-fit for each curve to test whether it meets the standard linear conformity requirements (R^2^ ≥ 0.8) before calculating the final *D*-values. Plotting and curve results are shown in Fig 4, and the calculated *D*-values, as well as the time to sterility assurance level (SAL) of 10^−6^ for different species are summarized in the table next to the graph. All three BIs responded to NO similarly, showing comparable *D*-values. Compared to *B. atrophaeus*, *B. subtilis* and *G. stearothermophilus* are slightly more resistant to NO sterilization cycle at RT, while *B. subtilis* and *G. stearothermophilus* performed very similarly.

Apex® Discs BI has one of the most resilient bioburdens and is designed for vaporized hydrogen peroxide sterilization (VHP). Our results directly show that RT NO sterilization eliminates viable spores (passed the BI test) using this highly resilient commercial BI. The calculated time to SAL indicated a 24-h sterilization cycle is sufficient to guarantee sterility even if a Log 12 bioburden is requested. Note that normally 55–65 °C is recommended as optimum for *G. stearothermophilus* culture. Our temperature, 50 °C falls within the organism’s growth range (30–75 °C) and is widely documented as an effective condition for colony forming [23,24]. In our studies, untreated positive controls were processed alongside all test samples at this temperature, resulting in consistent microbial recovery, which eliminated the risk of temperature-induced artifacts. Furthermore, this selection maintains experimental consistency across our broader research program, including our other ongoing investigations running NO sterilization cycle at maximum of 50 °C. Matching temperature will ensure our future cross-study compatibility.

### Clinically relevant bacteria *D*-value calculation

Although most sterilization methods use bacterial endospores as the bioburden, this approach has low correlation with healthcare species. Thus, we selected the top two bacterial species involved in infection-related deaths, *Staphylococcus aureus* (*S. aureus* Gram positive) and *Escherichia coli* (*E. coli* Gram negative) [25], to examine how vegetative bacteria respond to NO sterilization cycles. This will help us understand how NO sterilization can be used to control infections and disease spreading.

**Fig 5:**
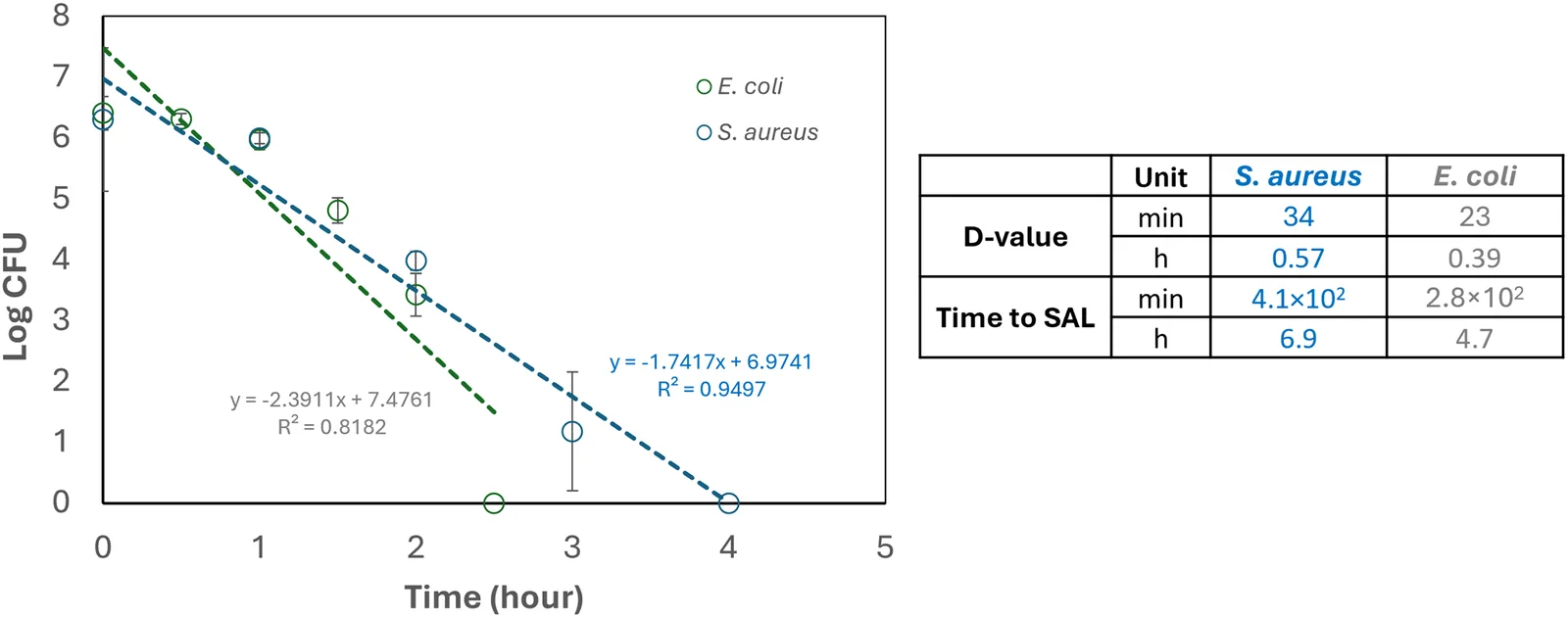
Calculation of *D*-values of two leading clinically relevant bacteria, *S. aureus* and *E. coli*, (n ≥ 3 for each type at each time point) undergoing RT NO sterilization cycle. Log of remaining CFU over time (mean ± SD) and kill curves are shown in the plot and the calculated *D*-values are summarized in the table.

The plate counting results with the corresponding dilution ratio are shown in Supporting information (S Table 6-7 in S1 File). Our results reveal that NO sterilization is very effective in inactivating both bacterium types (Fig 5). Compared with endospores (Fig 4), *D*-values of vegetative bacteria were significantly shorter. Note that R^2^ of *E. coli* is relatively low. Unlike bacterial endospores or *S. aureus*, which can maintain high viability in a dehydrated state, *E. coli* rapidly loses activity upon atmospheric drying even without treatment. To isolate NO’s effects, *E. coli* trials were executed using wet suspension, meaning the sterilization cycles started immediately following inoculation. Since NO operates as a gaseous sterilant, the presence of a liquid droplet introduces a mass transfer boundary, where NO must first dissolve into and diffuse through the aqueous phase to reach the embedded bacteria. The observed time lag of reduction curves at the initial stage and smaller R^2^ in fact presents a shoulder effect or non-linear deviation as recognized by ISO 14161. This setup was intentionally designed as a worst-case scenario challenge test. Our data demonstrates that despite the physical protection afforded by the liquid droplet barrier, gaseous NO effectively overcame this boundary layer. To establish SAL of 10^−6^ using both Gram-positive and Gram-negative bacteria as bioburden, 5–7 h under RT is recommended.

### Study limitations and future directions

This investigation was primarily designed as a fundamental, proof-of-concept mechanistic study rather than a final sterility validation protocol. The primary objective was to establish a scientific groundwork demonstrating that controlled NO conditions, delivered via controlled volume of Sterile Solution® in a defined closed space, can successfully achieve complete inactivation of highly resilient BIs under mild environmental conditions. However, we understand the constrained sample size used in this study only represents a preliminary phase of evaluation. Ongoing studies in our laboratory are focused on expanding sample sizes and conducting comprehensive tests in strict compliance with the industrial performance validation guidelines.

Under the FDA’s established hierarchy of microbial resistance, bacterial endospores are considered as the most resilient biological entities to chemical and thermal inactivation. Our current data demonstrate that four commercial BIs evaluated in this study were completely inactivated by RT NO sterilization (Fig 3). *G. stearothermophilus*, *B. subtilis*, and *B. atrophaeus* displayed comparable resistance profiles (Fig 4). By demonstrating that controlled NO conditions can consistently achieve inactivation of these high-resistance strains, we are trying to establish a foundational understanding of the broad-spectrum sporicidal potential of this new sterilization modality. However, note that proposing or validating a specific BI to represent the worst-case scenario challenge for a novel sterilization modality is a stringent, multi-variable, and systematic process required for formal regulatory approval, which is not the scope of this early-stage study. Characterizing a customized process challenge device (PCD) remains a core focus of our ongoing research in our research center.

Although standards such as ISO 11737−1 and USP < 61 > recommend a minimum incubation period of 2–3 days for general bioburden enumeration, we acknowledge that a 3-day negative result (clear plates) is not universally acceptable under other stringent standards, such as USP < 71 > . We recognize that our current data is preliminary, and more comprehensive, long-term incubation studies are underway for sterility tests.

## Conclusions

Confirmed by overkill half-cycle test, RT NO sterilization cycles can inactivate the commercial BIs that are currently widely used to validate steam, EtO and VH_2_O_2_ sterilization methods, including *B. atrophaeus*, *B. subtilis*, and *G. stearothermophilus*, as well as achieve SAL of 10^−6^ within 18 h*.* Here we proved that NO inactivates all three endospores following a Log-linear reduction pattern according to ISO 11138−1 guidelines. And RT NO sterilization is highly effective at eliminating clinically relevant bacteria *S. aureus* and *E. coli*. The calculated *D*-values show that vegetative bacteria, *S. aureus* and *E. coli*, are much more sensitive to RT NO sterilization cycles compared with endospores. Thus, we conclude that NO can potentially be used as a novel gaseous sterilant for RT sterilization applications.

## Supporting information

S1 File S. Tables 1-7. Physical parameter monitoring and plate counts raw data. (DOCX)
