# Supplementary material for: Using nitric oxide (NO) for gas sterilization at room temperature: A novel sterilization modality
Source: PLoS One. 2026 Aug 21;21(8):e0355735. doi: 10.1371/journal.pone.0355735 (PMC13496434; doi:10.1371/journal.pone.0355735)
Supplement: Supplementary file 1 [file pone.0355735.s003.pdf]

Dear Editor and Reviewers,

We would like to express our sincere appreciation for your time, effort, and constructive feedback on our manuscript. These comments help us improve both the content and the overall flow of our work. In response to the feedback, we have significantly revised the manuscript. Specifically, we have expanded the text to provide comprehensive information regarding the chemical properties and delivery mechanisms of NO, alongside detailed methodologies for both the sterilization set-up and the bacterial experiments, especially clarification of sampling and adjustment in data presentation. A point-by-point response to each of the specific comments is provided below.

Sincerely,

Reviewer #1:

Response:

We sincerely thank the reviewer for all the constructive feedback.

Sorry for the previous confusion. We would like to clarify that the scope of the current manuscript is fundamentally a proof-of-concept, mechanistic investigation rather than a regulatory filing or a medical device product validation. The true intent and primary goal of this work is to demonstrate the fundamental fact of NO's effect to highly resilient bacterial endospores under mild, ambient-temperature conditions. Our ongoing work is explicitly focused on translating these mechanistic findings into scaled, optimized, and robustly reproducible engineering platforms.

Accordingly, we have thoroughly revised the manuscript, especially insofar as to provide a more precise and conservative description of our experimental set-up, ensuring the scope of our conclusions matches the preliminary nature of the current data. Our detailed, point-by-point responses to the individual reviewer comments are provided below:

Lines 112-113: Spore count protocol should be detailed for spore count to be successful and reproducible. Otherwise, there could be wrong counting. Was the manufacturer recommendation followed? Two different manufacturers were used.

Response: Thanks for comments. We added the details in the method section.

Revised version:

[Type here]

To evaluate the sporicidal efficacy of NO sterilization, a series of commercial BIs meeting the minimum performance criteria of ISO 11138-1 were utilized. The commercial indicators evaluated included: *Bacillus subtilis* paper strips (Crosstex, REF: BS52306, LOT: B012, population: 2.0×10^6 CFU/carrier), *Bacillus atrophaeus* 6 mm paper discs (Crosstex, REF: BG-106D, LOT: ND105, population: 3.5×10^6 CFU/carrier), *Geobacillus stearothermophilus* 3 mm paper discs (Crosstex, REF: DS18-06, LOT: S2200502, population: 1.8×10^6 CFU/carrier), and Apex® Stainless Steel Discs inoculated with *B. atrophaeus* (Mesa Labs, REF: GRS-090, LOT: AG-041, population: 2.9×10^6 CFU/carrier). All commercial BIs were stored, handled, and maintained in strict accordance with the manufacturers' specifications, using resealable airtight plastic storage bags supplemented with desiccant pouches at 2 – 8 °C to preserve spore dormancy. For customized BIs, commercial spore suspensions of *B. subtilis* 5230 (Mesa Labs, REF: SS5230E/8, LOT: SS5230-491, stock: 1.9×10^8 CFU/0.1 mL) and *G. stearothermophilus* 7953 (Mesa Labs, REF: SSSE/7, LOT: SSS-981, stock: 2.3×10^7 CFU/0.1 mL) were used following Mesa Labs' Technical Report, *Product Inoculation*. Working suspensions were prepared by performing a 10-fold dilution in 40% isopropanol to achieve a target density profile in the ~10^7 CFU/0.1 mL range. A 15 µL aliquot of the prepared suspension was then aseptically inoculated onto the bottom of a 4-mm high, 4 mm diameter polypropylene cup as substrate. Following ambient air-drying under a laminar flow hood for 6 hours, these custom carriers were stored under refrigerated desiccant conditions (2 – 8 °C) no more than 2 weeks before use. Prior to experimental deployment, the baseline viable spore populations on all types of BIs were verified via mechanical elution, serial ten-fold dilution, and agar plate enumeration in triplicate to confirm compliance with manufacturer-stated specifications.

Line 125: no details for spore inoculation (procedure, carrier material, drying, etc.)

Response: Thanks for comments. We added the details in the method section.

Revised version:

To prepare the working bacterial suspensions, isolated colonies from fresh agar cultures were harvested and suspended in the phosphate-buffered saline (PBS). To ensure a homogenous suspension free of microbial clumping, the mixture was thoroughly disrupted using a vortex mixer. The initial population density was standardized photometrically by measuring the optical density at 600 nm (using OD600 of 1.0 corresponding to 5×10^8 CFU/mL as the rough reference). In accordance with USP <61>, the exact viable concentration of this baseline inoculum was verified via ten-fold serial dilutions in PBS (shown in Table 1), followed by spread-plating onto Mueller–Hinton agar before each experiment. The plates were incubated at 35 °C for 12 hours or 20 hours (for *E. coli* and *S. aureus*, respectively) to confirm the starting

population via direct CFU counting. A standardized volume of the bacterial suspension (normally 2 µL in our study) was aseptically inoculated onto the interior surface close to the bottom of 1.8 mL sterile glass sample vials. These prepared sample vials were then immediately subjected to the varying NO sterilization cycles for kinetic evaluation.

Table 1. Illustration of different dilution preparation

Dilution ratio	PBS in Vial I	V from Vial I transferred to Vial II	PBS in Vial II	V for inoculation	Extra PBS for inoculation
1:10000	1 mL	10 µL	990 µL	10 µL	90 µL
1:1000	1 mL	10 µL	900 µL	100 µL	0 µL
1:100	1 mL	N/A	N/A	10 µL	90 µL
1:10	1 mL	N/A	N/A	100 µL	0 µL
N.D.	100 µL	N/A	N/A	100 µL	0 µL

N.D. represents no dilution

Figure 1. NO sterilization device is not well described. More details should be given in order to understand how the BIs and bacterial strains are exposed. The illustration is too simplified.

Response: Thanks for comments. We edited the image and the caption, and adjusted the manuscript to be more specific.

Revised version:

NO sterilization chamber architecture and parameter monitoring

NO generation and subsequent sterilization cycles were conducted within a localized, completely sealed vessel possessing an internal enclosure volume of 8 oz (or 236 mL), see Figure 1. NO was delivered in situ via the addition of a 0.2 M Sterile Solution® formulation. Specifically, a precise aliquot of 2000 µL of freshly prepared Sterile Solution® was dispensed into a 1-inch diameter containment tray situated at the base of the unit. A representative challenge load, a 5 mL polypropylene (PP) target vial, with BI placed inside the sample vial, were positioned within the vessel to be sterilized. When evaluating multiple enveloped BIs, BIs were distributed well to prevent physical overlapping or stacking artifacts, thereby ensuring unhindered, uniform gas-phase mass transfer to all inoculation sites. After component loading, the chamber was sealed to establish an airtight environment, and the kinetic exposure period was initiated immediately upon closure. All sterilization cycles were conducted at ambient RT (defined as 18 – 23 °C in our lab space). To ensure environmental validation, temperature profiles were continuously monitored and recorded per min using calibrated Govee™ digital sensors positioned both adjacent to and inside the active sterilization units.

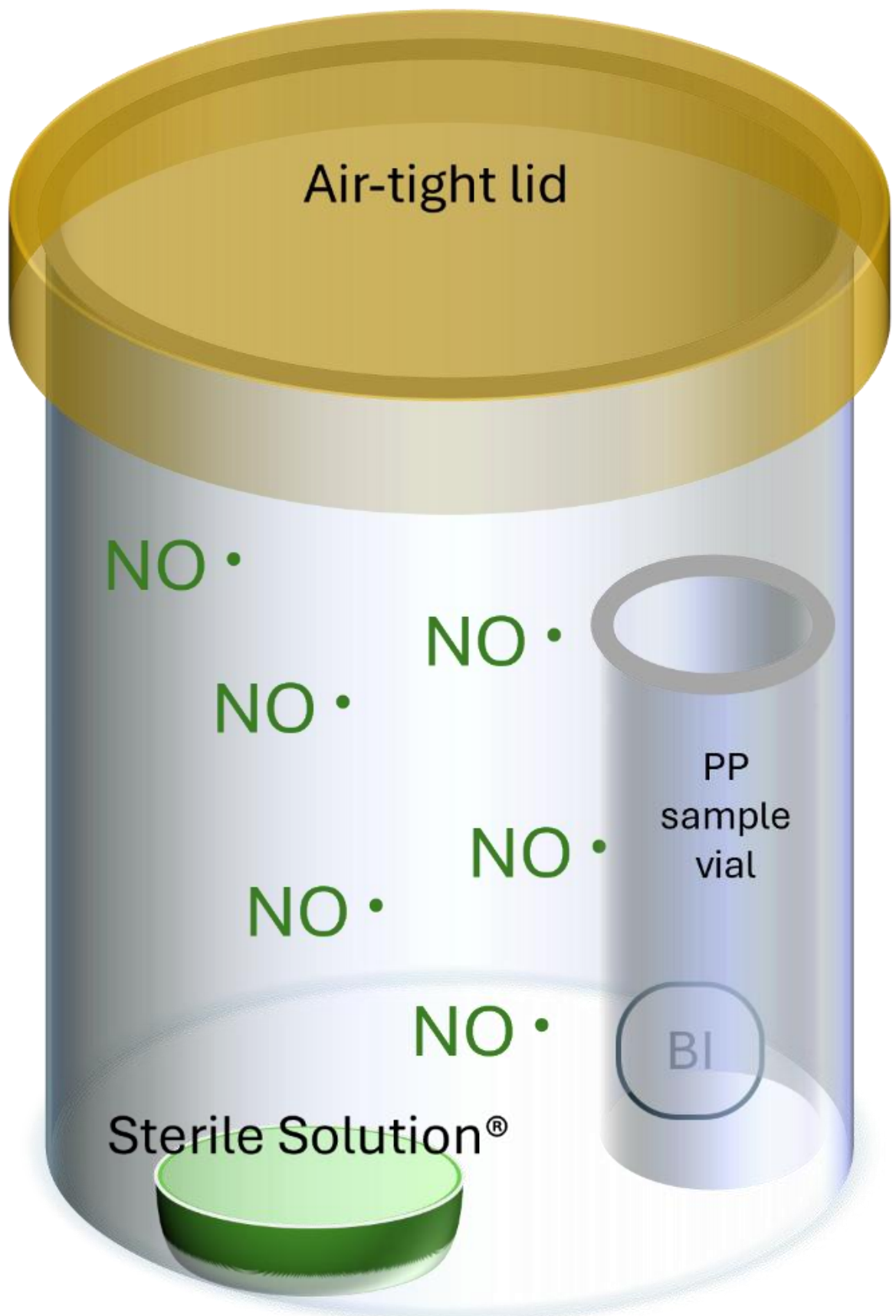

Figure 1. Representative setup of the Sterile State™ NO sterilization device. A polypropylene (PP) sample vial (the target object to be sterilized) containing a spore-inoculated commercial BI was loaded into the airtight 8 oz sterilization device. NO was delivered via 2 mL of 0.2 M Sterile Solution®. The container was securely sealed, allowing the internal NO concentration to reach above 100 PPM. The sterilization cycle was conducted at RT. Different sterilization durations were achieved by controlling the time when to open the vessel.

Line 144: "N=3 different BIs were investigated". Triplicate is ok, but how was the exposure? inside the same container or in 3 different real replicates in separate containers?

Response: Thanks for comments. We understand our system is different from conventional systems, where our individual sterilization unit is an independent sterilization cycle, which can confuse readers. We edited the manuscript to specify the sample numbers under the CFU count tables to explain. Essentially, we have three BIs loaded in each sterilization unit, and when we repeat the study there will be another three BIs loaded. For bacterial experiments, only one aliquot of Sterile Solution® is loaded in the sterilization unit, and three same set-up units for one time point. When we repeat the study, there will be another three sterilization units set up.

[Type here]

Revised version:

Each sterilization unit contains three BI samples. Different sterilization unit numbers indicate different independent repeats of the study.

Each sterilization unit contains one bacterial suspension aliquot. Different sterilization unit numbers indicate different independent repeats of the study.

Lines 145-146: I don't understand the aim of this test. This is an inhibition test, so are you trying to test inhibition of what? the BI carrier inoculated with the spore suspension? or the possible absorption of NO on the BI carrier?

Response: Thanks for reminder. Sorry for the confusion. This is basically a test for viability of the spore after exposed to NO to confirm lethality of the spores in the BI. Although liquid immersion culture (turbidity testing) is a traditional approach for qualitative BI evaluation, we used the direct plating method (agar contact method) for our qualitative and binary assessments, which is based on the microbial recovery principles outlined in USP <1227> and ISO 11737-1 for evaluating surface contamination and carrier validation. Placing the carrier directly onto Mueller–Hinton agar allows for the unambiguous observation of localized germination and visible colony formation originating directly from the carrier surface (as illustrated by the positive controls in Figure 2). If a BI is not fully inactivated, the surviving spores germinate normally to form distinct, identifiable colonies, whereas completely inactivated BIs remain entirely clear of growth as shown in Figure 2. In addition, one of our on-going studies is carrier's effect, which is not included in this manuscript. To avoid misunderstanding, we revised our method section as:

Revised version:

Qualitative evaluation of BI sterility via direct plating

To assess the binary (growth/no-growth) endpoint of the BI inactivation, i.e. to verify complete microbial inactivation, a direct plating method was employed. This approach was selected in accordance with the microbial recovery and neutralization principles outlined in USP <1227> and ISO 11737-1 to mitigate potential sterilant carryover effects. Specifically, after exposure to the designated NO sterilization cycles, the treated BI carriers were aseptically retrieved and transferred directly onto the surface of Mueller–Hinton agar plates (Hardy Diagnostics). The plates were inverted and incubated under humidified conditions at either 35 °C (for *B. atrophaeus* and *B. subtilis* BIs) for 3 days then to 50 °C (for *G. stearothermophilus* BI) for 3 days. Qualitative evaluation was performed via visual inspection. Complete absence of any localized germination or macroscopically visible colony formation surrounding or originating

from the carrier substrate after the incubation period indicates no-growth, while microbial growth is characterized by the proliferation of distinct colonies emerging directly from the carrier. All plates exhibiting no initial growth were maintained in the incubator for an extended validation period of no less than 3 days.

Line 148: The optimum growth temperature for Geobacillus stearothemophilus is 60ºC, I'm surprised and wondering about the choice of 50ºC. This 10ºC shift could slow down the growth rate, even more in a reduced 3-day incubation (as compared to the appropriate conventional 7 days), written in line 149.

Response: We appreciate the reviewer's diligent observation. While 55-65 ºC represents the peak optimum kinetic range for this highly thermophilic species, *G. stearothermophilus* possesses a broad growth temperature profile typically extending from 30 -75 ºC [Ref 1 and 2]. In our study, to ensure validation integrity, untreated positive controls were processed alongside all test samples at 50 °C, yielding robust and consistent microbial recovery. Furthermore, our temperature selection was to ensure experimental consistency across our broader research program, where our other ongoing investigations evaluate NO sterilization operating at maximum of 50 °C. Using a uniform incubation temperature avoids temperature-induced recovery artifacts and allows for future cross-study compatibility. To clarify this for the reader, we have updated the manuscript to discuss this.

Ref 1: BacDive. *Geobacillus stearothermophilus* (Strain ID 1420). Braunschweig, Germany: Leibniz Institute DSMZ; [updated 2026 May 29; cited 2026 Jun 22]. Available from: https://bacdive.dsmz.de/strain/1420

Ref 2: Mtimet, Narjes, et al. "Die another day: fate of heat-treated Geobacillus stearothermophilus ATCC 12980 spores during storage under growth-preventing conditions." *Food microbiology* 56 (2016): 87-95.

Revised version:

Normally 55 – 65 °C is recommended as optimum for *G. stearothermophilus* culture. Our temperature, 50 °C falls within the organism's growth range (30-75 °C) and is widely documented as an effective condition for colony forming [Ref 3]. In our studies, untreated positive controls were processed alongside all test samples at this temperature, resulting in consistent microbial recovery, which eliminated the risk of temperature-induced artifacts. Furthermore, this selection maintains experimental consistency across our broader research

program, including our other ongoing investigations running NO sterilization cycle at maximum of 50 °C. Matching temperature will ensure future cross-study compatibility.

[Ref 3] Wells-Bennik, Marjon HJ, et al. "Heat resistance of spores of 18 strains of Geobacillus stearothermophilus and impact of culturing conditions." *International journal of food microbiology* 291 (2019): 161-172.

Lines 156-157: "Based on the assumption of Log-linear inactivation kinetics". Not sure this assumption is correct for chemical processes. Has this been corroborated before this assumption was made for these experiments?

Response: Thanks for comments. Sorry for the confusion. Our analysis followed the standard framework outlined in ISO 11138-1 (Annex C), which mandates evaluating data against a first-order Log-linear model to establish a baseline for linear regression analysis. We have reworded this sentence in the revised manuscript to clarify that linearity was treated as a model hypothesis to be statistically verified.

Revised version:

In accordance with ISO 11138-1 guidelines, we evaluated the goodness-of-fit for each curve to test whether it meets the standard linear conformity requirements ($R^2 \geq 0.8$) before calculating the final D-values.

Line 161:

Lines 163-165: More information should be provided regarding the analyzer and internal data. Confidentiality in understood but the concentration of NO is crucial to understand and discuss the sterilization system.

Line 168: "levels outside of this range were marked as Fail." It would be interesting to see this range with more precision. Thresholds with exact numbers if the safety and robustness of the process is being analyzed.

Response: Thanks for comments. We detailed the method section, added the principle of NO delivery system and how critical level is determined in the discussion as well as user safety in the revised version.

Revised version:

NO level measurement

Quantification of NO level was performed using a Sievers Chemiluminescence Nitric Oxide Analyzer (NOA 280i; GE Analytical Instruments) [Ref1 and 2]. The instrument operates based on a gas-phase chemiluminescent reaction between NO and ozone, emitting light that is detected by a cooled photomultiplier tube to generate a proportional electrical signal. Prior to analysis, a two-point calibration protocol was executed by a zero-gas baseline calibration using a filtered sweep gas and followed by a span calibration using a certified standard reference gas containing 45 PPM of NO (GE HealthCare). To monitor internal concentrations during processing, the NOA sampling line was coupled to the sterilization chambers via a customized, small-gauge needle interface. Real-time data acquisition was recorded at a frequency of 1 Hz (PPM per second) and archived in raw, unmodifiable formats for trace validation. Each independent sterilization unit was sampled for a fixed duration of 30 s, followed by a 2-min purging interval between consecutive units to clear the sampling line and prevent cross-contamination. The representative NO level for each experimental run was determined by averaging the stable concentration profile obtained during the initial 10 s data integration window. Our internal reference threshold for effective NO sterilization was established at 100 PPM. We designate this level as the empirical sterilization unit (artificial unit or a.u.). This benchmark was selected based on our observations that NO exhibits a high sporicidal efficiency against resilient bacterial endospores around this concentration without causing discoloration of the target. Consequently, the real-time internal concentrations quantified by the NOA 280i were continuously evaluated and reported against this reference baseline.

[Ref]

1. Hampl V, Walters C, Archer S. Determination of nitric oxide by the chemiluminescence reaction with ozone. Methods in Nitric Oxide Research. Feelisch M, Stamler JS. 1996.
2. Reynolds MM, Frost MC, Meyerhoff ME. Nitric oxide-releasing hydrophobic polymers: preparation, characterization, and potential biomedical applications. Free Radical Biology and Medicine. 2004 Oct 1;37(7):926-36.

Added in the discussion:

Like other chemical sterilization methods, sterilization efficacy of gaseous NO depends on concentration, humidity, and exposure duration. However, NO's highly reactive with an extremely short half-life of several seconds under oxygenated conditions [Ref1]. Our system uses a continuous NO generation source Sterile Solution®, a specialized formulation of S-nitrosothiol solution, that can provide sustained NO generation for days [Ref2]. According to

established kinetic modeling of NO concentration profiles based on equilibrium of generation and consumption of NO, this constant flux allows us to achieve a steady-state where the internal NO concentration can reach a predictable and sustained plateau for sterilization applications [Ref 3 and 4]. In addition, due to the aqueous nature of Sterile Solution®, the internal RH concurrently reaches a stable equilibrium simply determined by the fixed liquid to headspace volume ratio. Therefore, this controlled configuration transforms a highly instable gas into a controlled sterilization environment capable of inactivating microbial reliably.

[Ref]

1. Palmer, Richard MJ, A. G. Ferrige, and Salvador Moncada. "Nitric oxide release accounts for the biological activity of endothelium-derived relaxing factor." *Nature* 327.6122 (1987): 524-526.
2. He, Weilue, and Megan C. Frost. "Direct measurement of actual levels of nitric oxide (NO) in cell culture conditions using soluble NO donors." *Redox biology* 9 (2016): 1-14.
3. Lancaster Jr, J. R. "A tutorial on the diffusibility and reactivity of free nitric oxide." *Nitric oxide* 1.1 (1997): 18-30.
4. LAURENT, Michel, Michel LEPOIVRE, and Jean-Pierre TENU. "Kinetic modelling of the nitric oxide gradient generated in vitro by adherent cells expressing inducible nitric oxide synthase." *Biochemical Journal* 314.1 (1996): 109-113.

Line 169: Data and statistical analyses. Information is very scarce. Tables with values for all measures and counts should be given. N should be higher for this process to be approved or conclusions to be more robust and unquestionable.

Response: Thanks for comments. We added the data in the Appendix.

Line 178: “n=5 for each BI type”. In line 144, N was informed as 3, Is it 3 o 5? Still a low N.

Response: Thanks for comments. We want to clarify that the primary intent of this manuscript is not to formally validate our setup as a newly cleared compliance-ready sterilization modality. We are currently conducting a separate, dedicated project strictly adhering to ISO 11138 standards, using both the Holcomb-Spearman-Karber and Stumbo-Murphy-Cochran methods to fully validate sterilization efficacy and sterility assurance levels using an entirely different industrial setup. In fact, our preliminary data from the smaller sample sizes (n = 3 or n = 5) presented here align closely with the highly consistent trends observed in our latest larger-scale validation studies (not included in this manuscript). While we fully acknowledge that our current sample size does not meet the strict statistical frameworks, the consistency of our preliminary outcomes

strongly suggests a robust underlying phenomenon. In early-stage scientific evaluation, omitting reproducible, foundational observations solely on the basis of sample size risks overlooking viable technological breakthroughs. However, we understand reviewer's concern. We add discussions as:

Revised version:

Study Limitations and Future Directions

This investigation was primarily designed as a fundamental, proof-of-concept mechanistic study rather than a final sterility validation protocol. The primary objective was to establish a scientific groundwork demonstrating that controlled NO conditions, delivered via controlled volume of Sterile Solution® in a defined closed space, can successfully achieve complete inactivation of highly resilient BIs under mild environmental conditions. However, we understand the constrained sample size used in this study only represents a preliminary phase of evaluation. Ongoing studies in our laboratory are focused on expanding sample sizes and conducting comprehensive tests in strict compliance with the industrial performance validation guidelines.

Line 181: "3 most used bacterial endospores". This is true, but there is bibliography specifically dedicated to demonstrate that these species are used because they have shown to be highly resistant to the sterilization process being monitored. How do we know that this is the same for NO ba50 sed MO inactivation? Was this assessed? Can you share evidence or data?

Response: Thanks for comments. We completely agree that establishing whether standard BIs represent the *worst-case scenario* for a novel sterilant is critical in full validation. We are actively expanding our research to evaluate a wider spectrum of species. Meanwhile we believe our current selection of these widely accepted endospores provides highly rigorous evidence of NO robust antimicrobial efficacy for this stage of study. Under the FDA's established hierarchy of microbial resistance, bacterial endospores sit at the very top as the most difficult microorganisms to kill. Standard sterilization validation frameworks rely on the principle that demonstrating a Log 6 reduction against these highly resilient endospores serves as a conservative proxy for the inactivation of less resistant pathogens. In our previous research and screening phases, we evaluated the effects of NO across various microbial classes. We did observe that vegetative bacteria (bacillus, staphylococcus, MRSA, TB), viruses (both enveloped and non-enveloped), and fungi (yeast) are significantly more susceptible to NO, requiring significantly lower concentrations and shorter exposure times. Bacterial endospores have been observed consistently as the most resilient species to our NO-based process thus far. According to ISO11138 guidelines, it is neither practical nor standard practice to test every individual

microorganism. Instead, our study is based on demonstrating efficacy against recognized reference organisms that possess extreme resistance to standard physical and chemical stress mechanisms. By demonstrating that controlled NO conditions via Sterile Solution® can repeatably inactivate these three standard BIs, we establish a foundation for its sporicidal and broad-spectrum potential.

Meanwhile, we have updated the manuscript to explicitly discuss the FDA resistance hierarchy and clarify our scientific rationale:

Revised version:

Under the FDA's established hierarchy of microbial resistance, bacterial endospores are considered as the most resilient biological entities to chemical and thermal inactivation. Our current data demonstrate that four commercial BIs evaluated in this study were completely inactivated by RT NO sterilization (Figure 3). *G. stearothermophilus*, *B. subtilis*, and *B. atrophaeus* displayed comparable resistance profiles (Figure 4). By demonstrating that controlled NO conditions can consistently achieve inactivation of these high-resistance strains, we are trying to establish a foundational understanding of the broad-spectrum sporicidal potential of this new sterilization modality. However, note that proposing or validating a specific BI to represent the worst-case scenario challenge for a novel sterilization modality is a stringent, multi-variable, and systematic process required for formal regulatory approval, which is not the scope of this early-stage study. Characterizing a customized process challenge device (PCD) remains a core focus of our ongoing research in our research center.

Line 205: "the assumption of Log-linear inactivation kinetics". This assumption is quite risky since chemical processes tend to display non-linear behavior when survivor curves are drawn.

Response: We thank the reviewer for this important caution. We are sorry for creating confusion here. We did not intend to suggest that linearity should be blindly assumed before evaluating the empirical trends. We are talking about, from pure math point, to apply a linear regression model the underlying assumption for the test is linearity. Our analysis followed the standard framework outlined in ISO 11138-1 (Annex C), which mandates evaluating data against a first-order Log-linear model to establish a baseline for linear regression analysis. We have reworded this sentence in the revised manuscript to clarify that linearity was treated as a model hypothesis to be statistically verified.

Revised version:

In accordance with ISO 11138-1 guidelines, we evaluated the goodness-of-fit for each curve to test whether it meets the standard linear conformity requirements ($R^2 \geq 0.8$) before calculating the final D-values.

And we also rewrote the method part. Please go to the method part:

Construction of survivor curves and D-value calculation

Figure 3: Correlation (R2) of the Bacillus subtilis chart is quite low (0,88) and is actually showing a non-linear model. Also, according to ISO 11138 standard, for D-value calculation at least 20 BIs should be used. Here, even though information is a little confusing and scarce, 5 exposure points with triplicate will give = 15 BIs.

Response: Thanks for comments. According to ISO 11138-1 6.4.4, “The survivor curve, when plotted as a semi-logarithmic curve of the log10 of the viable test organism count against time, shall be linear with a coefficient of determination, R^2 , of at least 0,8”. Our result is not against it. But we agree that our sample size can cause inaccuracy of representation of the true value, and we understand the reviewer’s concern. We have addressed the sample size concern in the discussion. Meanwhile we have an ongoing new manuscript using n=20 samples and comparison of different methods. In fact, our currently preliminary data shows there is no significant difference between values calculated from small sample size vs large sample size. We are working on the new publication involving large sample size as well. We addressed this comment in *Study limitations and future directions* section.

Figure 4: Correlation (R2) of the E. coli chart is quite low (0,81) and is actually showing a non-linear model. Attention should be paid to this behavior for D-value calculation. Too many assumptions are being made and data is not consistent.

Response: Thanks for pointing this out. We agree that this initial *D*-value calculation was simplified. To clarify our experimental design, *E. coli* was selected primarily to evaluate antimicrobial efficacy. And more importantly, unlike spores or *S. aureus*, which remain alive when dry, *E. coli* rapidly loses viability upon dehydration. Therefore, to assess the effects of NO, treatment and sampling were conducted while the *E. coli* suspension aliquot was still wet. Because NO is a gaseous sterilant, it must first overcome the physical barrier of the liquid droplet before reaching the bacteria. This mass transfer resistance introduces uncertainty and time lags. The low R^2 value is therefore consistent with the physical status of our experimental design. We have expanded our discussion in the revised manuscript to address this more cautiously.

Revised version:

Note that R^2 of *E. coli* is relatively low. Unlike bacterial endospores or *S. aureus*, which can maintain high viability in a dehydrated state, *E. coli* rapidly loses activity upon atmospheric drying even without treatment. To isolate NO's effects, *E. coli* trials were executed using wet suspension, meaning the sterilization cycles started immediately following inoculation. Since NO operates as a gaseous sterilant, the presence of a liquid droplet introduces a mass transfer boundary, where NO must first dissolve into and diffuse through the aqueous phase to reach the embedded bacteria. The observed time lag of reduction curve at the initial stage and smaller R^2 in fact presents a shoulder effect or non-linear deviation as recognized by ISO 14161. This setup was intentionally designed as a worst-case scenario challenge test. Our data demonstrates that despite the physical protection afforded by the liquid droplet barrier, gaseous NO effectively overcame this boundary layer. To establish SAL of 10^{-6} using both Gram-positive and Gram-negative bacteria as bioburden, 5 to 7 h under RT is recommended.

In summary:
Experimental design is not well established and/or explained.
Risky assumptions are being made in order to make simplified conclusions.
Statistics is oversimplified and replicates (N) are too low. No raw data is shown.
Other microorganisms and spore species should be assessed in order to make conclusions about the sterilization capacity of the system.
D-value calculation should be re-analyzed because there are calculation assumptions that are not quite safe to be made and chart show that behavior is not linear.
Despite the understandable confidentiality given this new approach, more information should be given to evaluate the robustness and sterilization level achieved by this system.

Response: We sincerely thank the reviewer for the valuable insights and constructive feedback. Our detailed, point-by-point responses to each comment are provided above.

Reviewer #2:

Dear Editor, I appreciate the opportunity to participate in this review. The manuscript presents a safe and promising alternative for low-temperature sterilization, although still at an early stage of development. The following points require further clarification by the authors:

Response: We sincerely appreciate the reviewer's constructive and insightful comments. We fully agree with the assessment regarding the preliminary nature of this early-stage data. Accordingly, we are currently expanding our evaluation using larger sample sizes and more

stringent testing protocols to further validate the system. Below, we have addressed each point in detail.

Abstract and line 32: Typographical error: "…specifically21-23 °C…". Please separate the temperature values.

Response: We sincerely appreciate the editor's thorough review and diligence. The requested correction has been made in the revised manuscript.

Line 98: The biological indicators tested used paper discs and paper strips as supports. Was any measure taken to evaluate the possible residual effect of the sterilizing agent on the supports? For example, the use of a neutralizing agent? In addition to paper, was another support such as non-absorbent material considered? I kindly ask the authors to clarify how they discard the hypothesis of residual effect, or whether they consider this a variable to be studied in future experiments.

Response: We appreciate the reviewer's insightful perspective. Mesa Lab BI (BI IV in Figure 2) used in this manuscript is in the form of stainless steel disc. The impact of different BI carrier materials and potential chemical residuals are indeed our concerns. This topics is currently under investigation, and we are preparing a separate manuscript systematically evaluating material compatibility and residuals. NO is characterized by an exceptionally short half-life on the order of seconds. While most endogenous signaling molecules rely on specific enzymatic degradation to terminate their activity, NO undergoes rapid autoxidation to nitrite and ultimately nitrate, both of which are benign to the human body. In fact, there is no physiological neutralizing agent in the natural environment as they are not needed. Upon exposure to air, it degrades within seconds. To confirm this and assess potential residues, we utilized Lugol's solution, a strong reducing agent that can convert NO oxidation products back to NO for analysis [Ref]. Our current results demonstrate no detectable nitrite present on PP vial. Since we are more interested in materials that are not compatible with steam sterilization, most of our current focus is polymer-based materials. But we believe stainless steel and cellulose are important as well. We'd like to share the data with the reviewers as follows but not included in the manuscript:

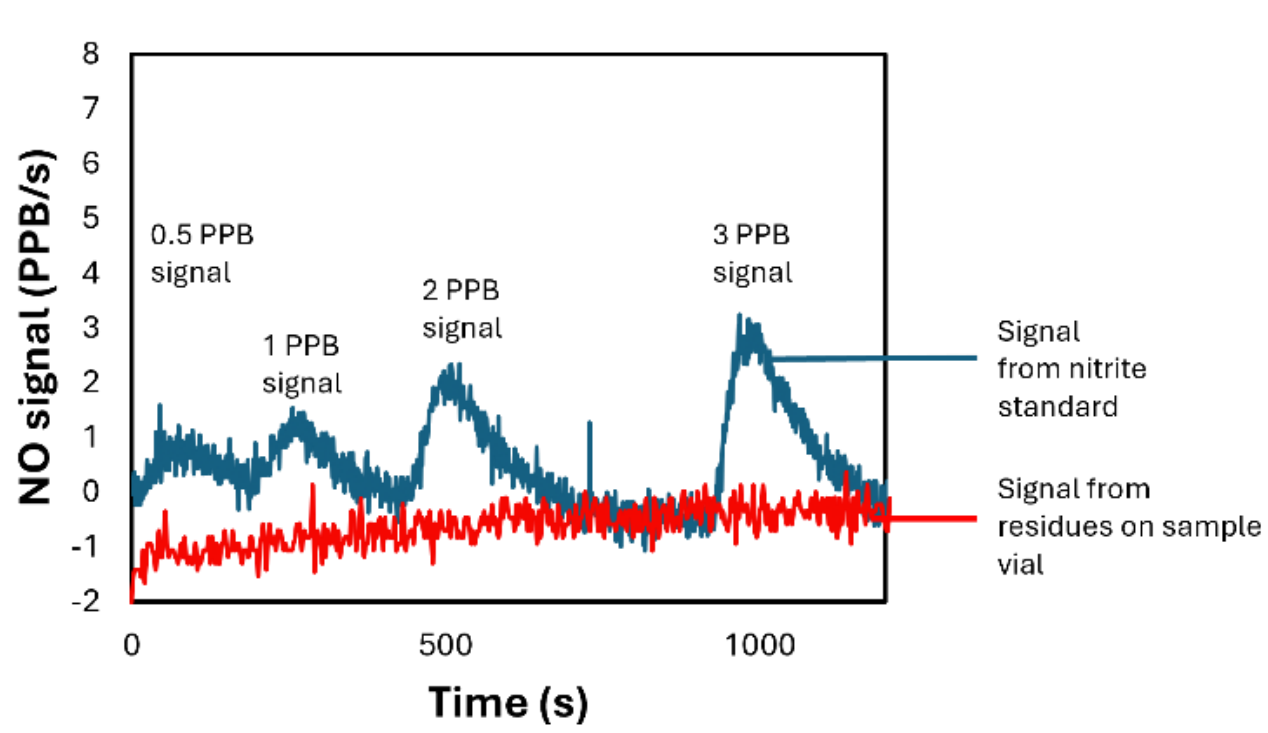

[Type here]

Residue analysis on the PP sample vial signal compared with signals from 0.5, 1, 2 and 3 PPB nitrite standards using Lugol solution and chemiluminescence.

Our assumption based on the chemistry is that any residual NO undergoes complete oxidation to nitrate. Detecting nitrate requires a stronger reducing agent, such as vanadium(III) chloride, which we plan to incorporate into our future work. However, nitrate exhibits an excellent safety profile, with acceptable human exposure limits exceeding 30 mg per day. Even if the entire volume of NO generated by 2 mL of 0.2 M Sterile Solution® were converted to nitrate, the total mass would yield only 12 mg, falling well within safe thresholds. Alternatively, NO could potentially induce nitration of exposed substrate materials, such as cellulose. To investigate this, our current study evaluated material compatibility using Fourier-transform infrared spectroscopy (FTIR) analysis. Results revealed minor change with no new absorbance peaks observed after NO treatment in the IR spectra. We'd like to share the data with the reviewers as follows but not included in the manuscript as more studies are going on currently for more comprehensive understanding.

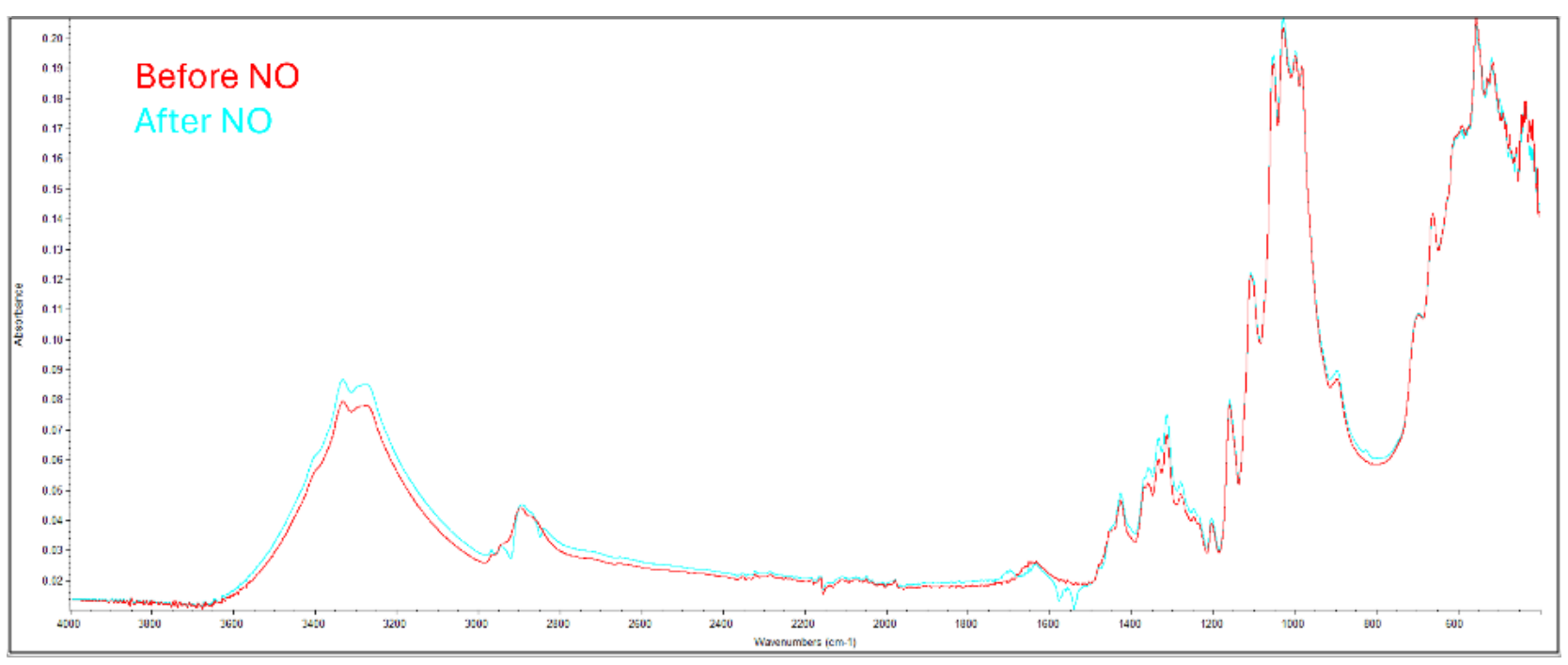

IR of cellulose paper-based material with and without NO sterilization cycle exposure.

We also did consider the sterilization efficacy across absorbent vs. non-absorbent carrier materials. While we observed that BIs on stainless steel substrates were inactivated slightly faster than those on absorbent carriers, this difference did not reach statistical significance thus far, maybe due to our small sample size. Additionally, we investigated antimicrobial or inhibitory effects from potential chemical residuals on porous matrices. Paper-based materials were first exposed to a standard NO sterilization cycle and subsequently used as substrates for BI inoculation and culture. We observed no bacterial inhibition or residual toxic effect from the paper-based BI carriers. Same results were observed when using stainless steel carrier materials to run the experiment.

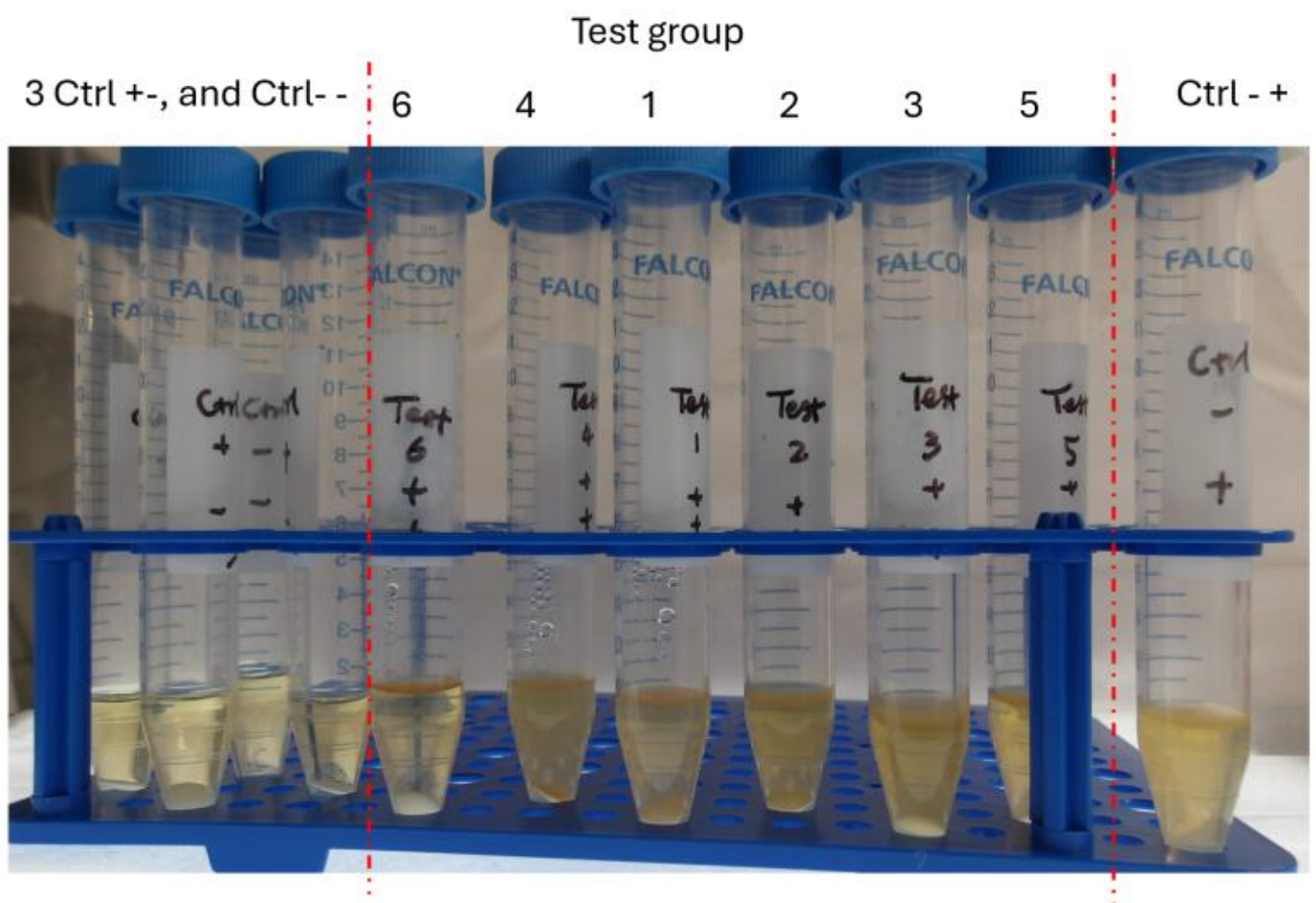

Potential residual on porous material's antimicrobial effect showing no residual effect was observed.

These data were excluded from the present manuscript as we are currently expanding these studies to provide a more comprehensive evaluation in a future publication.

Line 137: The role of the polypropylene tube was not clearly explained. According to the figure, the tube was placed in the device with a lid. Did the authors perform any complementary sterility tests? For example, inoculation in culture medium?

Response: We appreciate the reviewer pointing this out, and we apologize for any confusion caused by the original figure. We have revised the illustration and updated the corresponding text in the manuscript for clarity as:

[Type here]

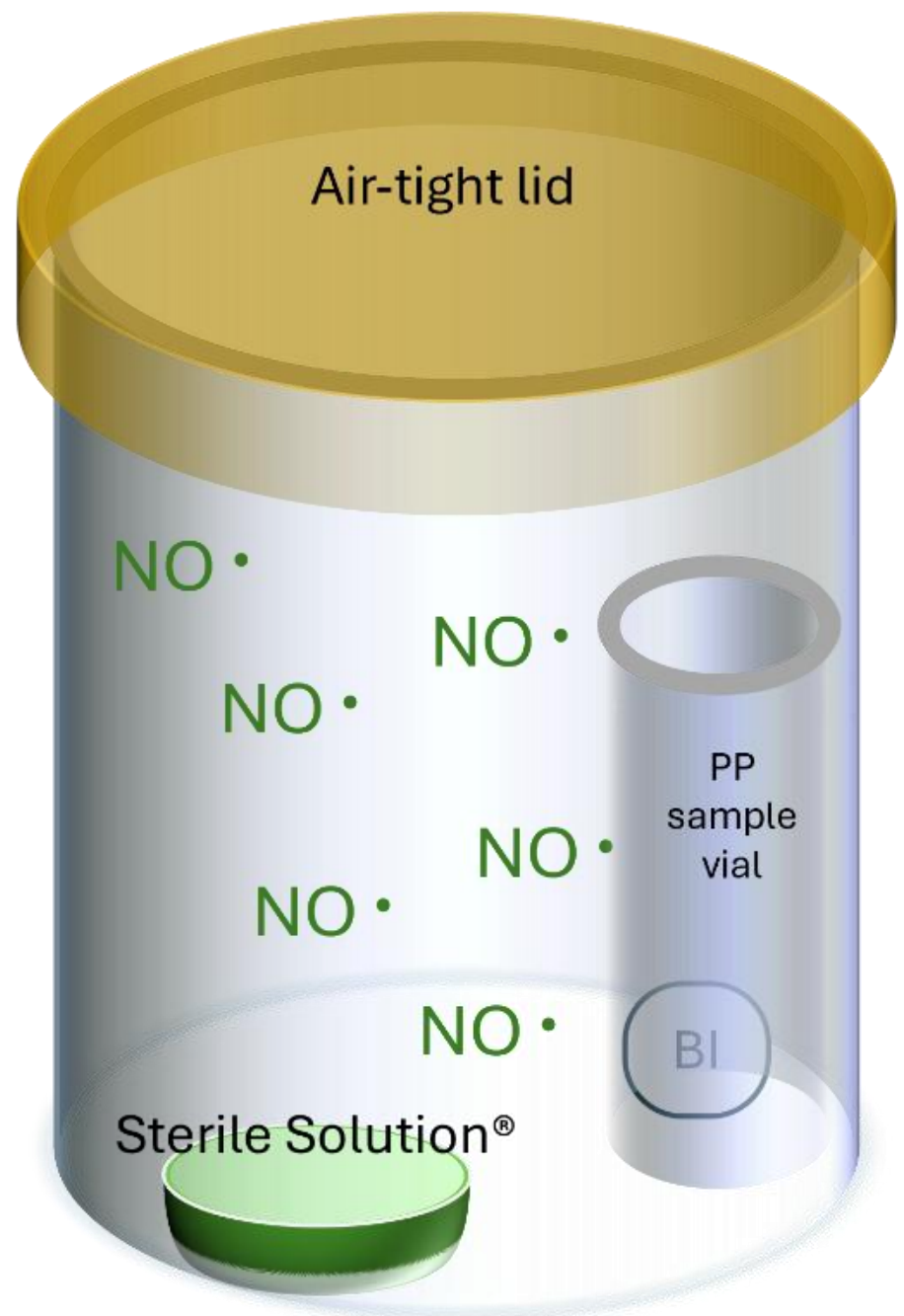

Figure 1. Representative setup of the Sterile State™ NO sterilization device. A polypropylene (PP) sample vial (the target object to be sterilized) containing a spore-inoculated commercial BI was loaded into the airtight 8 oz sterilization device. NO was delivered via 2 mL of 0.2 M Sterile Solution®. The container was securely sealed, allowing the internal NO concentration to reach above 100 PPM. The sterilization cycle was conducted at RT. Different sterilization durations were achieved by controlling the time when to open the vessel.

We did experiments using whole-vial immersion in culture media post-sterilization to monitor bacterial growth; no regrowth was observed after 7 days. We'd like to share with the reviewers as follows:

The whole PP sample vial was dropped into medium for culture.

Cloudy medium from positive control group after 7 days.

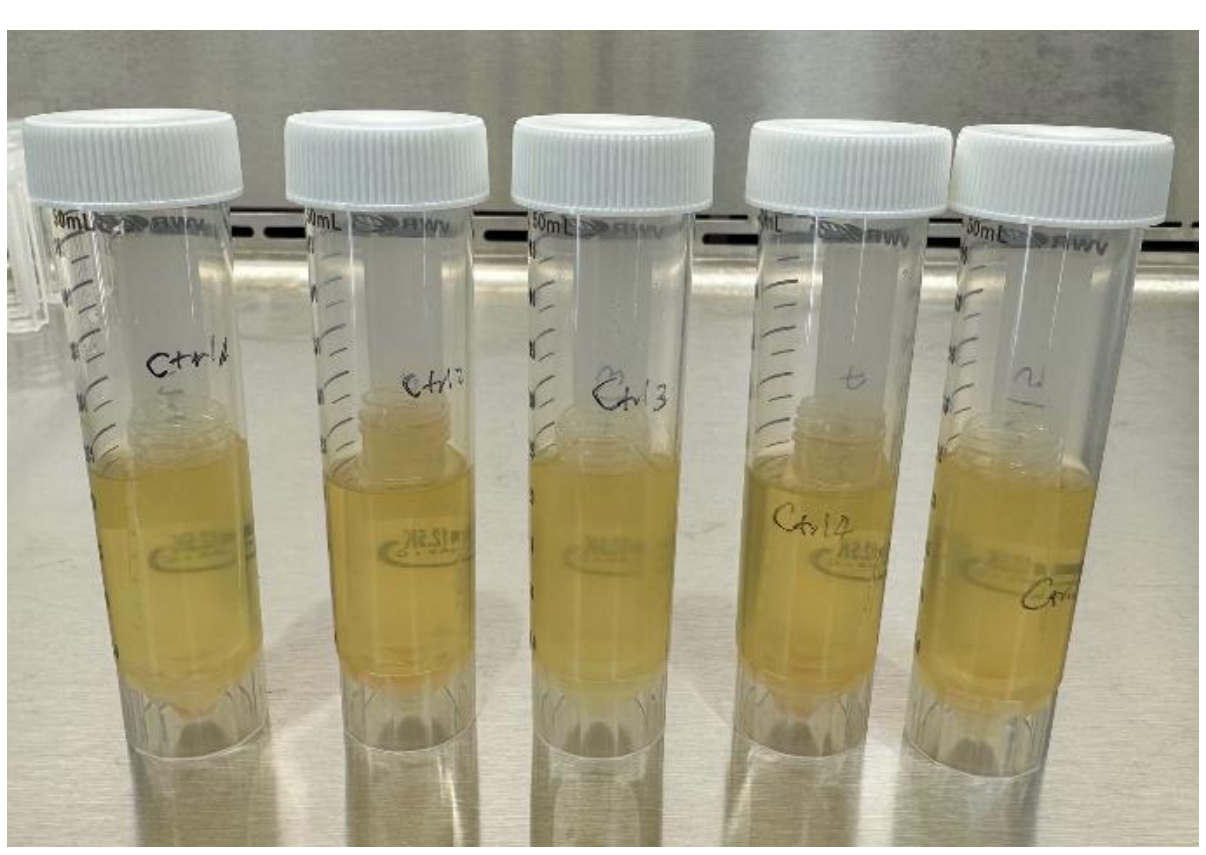

[Type here]

Clear medium from NO treated group after 7-day culture.

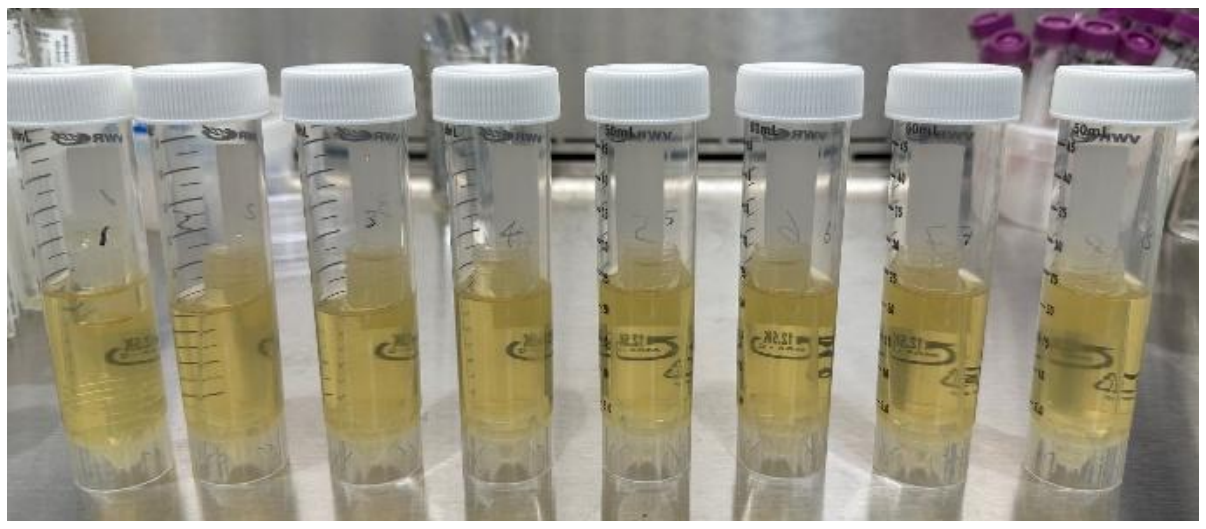

These data were excluded from the present manuscript because they fall outside our primary scope and will be better positioned in a future study investigating material residuals.

Lines 140 and 154: Please, provide the references supporting the method used, including any applicable pharmacopeial sources (e.g., USP, EP, or other recognized pharmacopeias/methods).

Response: Thanks for the great suggestion. We revised the entire section accordingly.

Revised version:

Bacterial cultivation and survivor population determination

To evaluate microbial inactivation kinetics across distinct exposure durations, multiple independent NO sterilization units were set up for different end points. The initial baseline population (N_0) was determined at $t = 0$. For spore BIs, samples were retrieved sequentially every 2 hours, whereas vegetative bacterial carriers were sampled at 30 or 60 min intervals. For each designated exposure time point, a minimum of three independent replicates (n = 3) were analyzed. Following exposure, treated BIs were aseptically retrieved and subjected to quantitative evaluation in accordance with the recovery and methodological validation principles of ISO 11737-1 and USP <61> / EP 2.6.12 for quantitative enumeration. More specifically, for viable count determination, treated BIs were transferred into 1 mL of PBS buffer and mechanically agitated to optimize microbial detachment, adhering to the extraction guidelines of ISO 11737-1. Serial ten-fold dilutions were performed in PBS to achieve an ideal target density of approximately 30 to 300 CFUs on each plate, trying to meet the statistical accuracy requirements of USP <61>. For those samples where the surviving population was expected to be lower than the 10^2 CFU level, no dilution was performed. The whole suspension was plated directly, and the corresponding BI carrier was also plated in the agar matrix to maximize the recovery efficiency.

Calculation of D-value

Based on guidelines specified in ISO 11138-1 (Annex C) and ISO 14161, microbial inactivation kinetics were analyzed by constructing semi-logarithmic survivor curves. The Log_{10} of the

surviving fraction ($Log_{10}N_t$) was plotted as a function of exposure time. The *D*-value, defined as the exposure time required to reduce the microbial population by 90% (one Log_{10} cycle), was determined using the standard equation: $D\text{-}value = t/(Log_{10}N_0 - Log_{10}N_t)$, where: t is the exposure time; N_0 is the initial viable population when t = 0; N_t is the surviving population at time of exposure. The *D*-value was derived as the negative reciprocal of the slope of the linear regression line fitted to the survivor plot data. In alignment with ISO 11138-1 (Section 6.4.4), a coefficient of determination ($R^2 \geq 0.8$) was established as the minimum threshold for linear conformity. Potential deviations from linearity were evaluated using the contextual guidance provided in ISO 14161. To achieve a theoretical SAL of 10^{-6} from a starting population (normally over 10^6 spores), the total required exposure time was calculated using the formula: Time to SAL of $10^{-6} = (Log_{10}N_0 - (-6)) \times D$-value.

Line 149: Did the authors conduct any preliminary tests to determine that three days is sufficient? There are publications showing that some bacteria require longer incubation times after being subjected to sterilization conditions. Additionally, some methodologies recommend thermal shock in sterility testing.

Response: Thanks for the great questions. The data presented in this manuscript represents our early-stage findings. To be more strictly, we now conduct experiments utilizing 7-day culture protocols for both drop test and plate enumeration. We observed no bacterial regrowth on plates if the plate remained clear after 3 days, with one exception that is under a drop test study involving 540 treatment samples, where one sample that was clear on Day 3 was confirmed as turbid on Day 5. While we do not claim that a 3-day culture period is sufficient for final sterility validation, these observations, when compared to control plates, clearly demonstrate the efficacy of NO against spores. We agree with the reviewer that many methodologies recommend thermal shock, it was not utilized in this study as we used the manufacturer's technical instructions (Mesa Labs, TS-120 Spore Suspension Technical Report https://mesalabs.com/hubfs/sporemind/public/TS-120%20Spore%20Suspension%20Technical%20Report.pdf). We added it to the method section. However, we fully agree with the reviewer that heat shock is more appropriate for spore enumeration, and we will take this into consideration for future work. To clarify this context, we have added the following statement to the discussion section:

Revised version:

Although standards such as ISO 11737-1 and USP <61> recommend a minimum incubation period of 2 to 3 days for general bioburden enumeration, we acknowledge that a 3-day negative result (clear plates) is not universally acceptable under other stringent standards, such as USP <71>. We recognize that our current data are preliminary, and more comprehensive, long-term incubation studies are currently underway at our research center for sterility tests.

[Type here]

Figure 2: Please specify each treatment in the figure description

Response: Thanks for the comments. We specified the treatment and conditions in the manuscript.

Revised version:

Treatment conditions: BIs of each type were loaded inside a 16 oz vessel without overlapping. NO was delivered through 2 mL of 0.2 M Sterile Solution®. Then the vessel was tightly capped to form an air-tight completely closed sterilization unit. After the sterilization cycle at RT for 12 h, the BIs were immediately collected and placed on agar plates for culture. In the positive control plates, all BIs grew after culture (after overnight culture), while all treated BIs in different repeats showed no bacterial growth (after over 3-day culture).

Appendix. Bacterial count

Table X. CFU count of the recovered *B. atrophaeus* after NO sterilization at RT

Time	Dilution ratio	Unit #	Sample 1	Sample 2	Sample 3	Average
0 h	10000	1	58	33	55	53
		2	89	112	55	
		3	15	36	28	
2 h	1000	1	71	84	93	82
	10000	1	33	42	36	38
		2	34	35	48	
4 h	10	1	12	13	20	83
		2	112	14	123	
		3	110	195	152	
6 h	N.D.	1	124	62	117	34
		2	0	0	0	
		3	0	5	0	
8 h	N.D.	1	0	0	0	0
		2	0	0	0	
		3	0	0	0	

Each sterilization unit contains three BI samples. Different sterilization unit numbers indicate different independent repeats of the study.

Table X. CFU count of the recovered *B. subtilis* after NO sterilization at RT

Time	Dilution ratio	Unit #	Sample 1	Sample 2	Sample 3	Average
0 h	10000	1	371	346	279	299
		2	292	252	289	
		3	250	338	270	
2 h	10000	1	178	94	87	151
		2	102	110	98	
		3	204	237	248	
4 h	1000	1	166	192	118	277
		2	368	258	201	
		3	294	453	447	
6 h	200	1	570	398	540	292
		2	128	243	343	
		3	163	145	104	
8 h	20	1	0	0	0	22

[Type here]

		2	0	0	0	
		3	76	89	33	
10 h	N.D.	1	0	0	0	0

Each sterilization unit contains three BI samples. Different sterilization unit numbers indicate different independent repeats of the study.

Table X. CFU count of the recovered *G. stearothermophilus* after NO sterilization at RT

Time	Dilution ratio	Unit #	Sample 1	Sample 2	Sample 3	Average
0 h	10000	1	113	89	108	128
		2	114	91	129	
		3	94	314	98	
2 h	10000	1	74	121	48	130
		2	280	178	177	
		3	94	122	73	
4 h	10000	1	57	36	124	72
	1000	2	198	189	252	208
		3	194	145	273	
6 h	10	1	56	82	10	57
		2	43	152	44	
		3	78	14	32	
8 h	N.D.	1	0	26	0	9
10 h	N.D.	1	0	0	0	0

Each sterilization unit contains three BI samples. Different sterilization unit numbers indicate different independent repeats of the study.

Table X. CFU count of the recovered *E. coli* after NO sterilization at RT

Time	Dilution ratio	Unit #	Sample 1	Sample 2	Sample 3	Average
0 h	10000	1	127	191	90	255
		2	314	463	345	
0.5 h	10000	1	224	212	219	204
		2	259	222	246	
		3	159	150	149	
1 h	10000	1	62	81	52	93
		2	152	148	104	
		3	63	95	81	
1.5 h	1000	1	78	62	160	65
		2	40	37	47	
		3	65	60	33	

[Type here]

2 h	100	1	103	22	39	26
		2	0	0	0	
2.5 h	N.D.	1	0	0	0	0
		2	0	0	0	

Each sterilization unit contains one bacterial suspension aliquot. Different sterilization unit numbers indicate different independent repeats of the study.

Table X. CFU count of the recovered *S. aureus* after NO sterilization at RT

Time	Dilution ratio	Unit #	Sample 1	Sample 2	Sample 3	Average
0 h	10000	1	8	10	21	199
		2	314	355	259	
		3	252	304	271	
1 h	10000	1	169	150	167	99
		2	93	55	62	
		3	53	80	59	
	1000	1	129	128	161	143
		2	106	195	185	
		3	136	133	113	
2 h	100	1	266	182	170	65
		2	140	138	103	
		3	318	204	212	
	1000	1	1	0	0	0
		2	0	0	0	
		3	0	0	1	
3 h	N.D.	1	5	0	85	61
		2	0	0	277	
4 h	N.D.	1	0	0	0	0

Each sterilization unit contains one bacterial suspension aliquot. Different sterilization unit numbers indicate different independent repeats of the study.
