# Supplementary material for: Using nitric oxide (NO) for gas sterilization at room temperature: A novel sterilization modality
Source: PLoS One. 2026 Aug 21;21(8):e0355735. doi: 10.1371/journal.pone.0355735 (PMC13496434; doi:10.1371/journal.pone.0355735)
Supplement: Supplementary file 2 [file pone.0355735.s001.docx]

**Supporting information**

**Using nitric oxide (NO) for gas sterilization at room temperature: a novel sterilization modality**

Weilue He*, Julie Osborne, Megan C. Frost*

R&D Department of Sterile State Inc., Grand Rapids, Michigan, United State of America

* Corresponding author

E-mail: weilue@sterilestate.com (WH) and mfrot@sterilestate.com (MF)

S. Table 1. Temperature and RH monitoring data

|  | Sample 1 | | Sample 2 | | Sample 3 | |
| --- | --- | --- | --- | --- | --- | --- |
| Time Abs | Temp (°F) | Humid (%) | Temp (°F) | Humid (%) | Temp (°F) | Humid (%) |
| 2/27/2025 8:00 | 68.18 | 24.8 | 68 | 25.2 | 68 | 25.3 |
| 2/27/2025 8:01 | 68.18 | 24.7 | 68 | 25.2 | 68 | 25.2 |
| 2/27/2025 8:02 | 68.18 | 24.8 | 68 | 25.3 | 68 | 25.2 |
| 2/27/2025 8:03 | 68.18 | 24.7 | 68 | 25.2 | 68 | 25.2 |
| 2/27/2025 8:04 | 68.18 | 24.7 | 68 | 25.2 | 68 | 25.2 |
| 2/27/2025 8:05 | 68.18 | 24.7 | 68 | 25.3 | 68 | 25.2 |
| 2/27/2025 8:06 | 68.18 | 24.8 | 68 | 25.2 | 68 | 25.2 |
| 2/27/2025 8:07 | 68.18 | 24.8 | 68 | 25.3 | 68 | 25.3 |
| 2/27/2025 8:08 | 68.18 | 24.8 | 68 | 25.2 | 68 | 25.2 |
| 2/27/2025 8:09 | 68.18 | 24.7 | 68 | 25.3 | 68 | 25.2 |
| 2/27/2025 8:10 | 68.18 | 24.8 | 68 | 25.2 | 68 | 25.3 |
| 2/27/2025 8:11 | 68.18 | 24.8 | 68 | 25.3 | 68 | 25.3 |
| 2/27/2025 8:12 | 68.18 | 24.7 | 68 | 25.2 | 68 | 25.2 |
| 2/27/2025 8:13 | 68.18 | 24.8 | 68 | 25.2 | 68 | 25.3 |
| 2/27/2025 8:14 | 68.18 | 24.8 | 68 | 25.3 | 68 | 25.3 |
| 2/27/2025 8:15 | 68.18 | 24.8 | 68 | 25.2 | 68 | 25.3 |
| 2/27/2025 8:16 | 68.18 | 24.8 | 68 | 25.3 | 68 | 25.2 |
| 2/27/2025 8:17 | 68.18 | 24.8 | 68 | 25.2 | 68 | 25.3 |
| 2/27/2025 8:18 | 68.18 | 24.8 | 68 | 25.2 | 68 | 25.2 |
| 2/27/2025 8:19 | 68.18 | 24.7 | 68 | 25.3 | 68 | 25.3 |
| 2/27/2025 8:20 | 68.18 | 24.7 | 68 | 25.3 | 68 | 25.3 |
| 2/27/2025 8:21 | 68.18 | 24.7 | 68 | 25.2 | 68 | 25.3 |
| 2/27/2025 8:22 | 68.18 | 24.7 | 68 | 25.2 | 68 | 25.2 |
| 2/27/2025 8:23 | 68.18 | 24.7 | 68 | 25.2 | 68 | 25.2 |
| 2/27/2025 8:24 | 68.18 | 24.7 | 68 | 25.2 | 68 | 25.3 |
| 2/27/2025 8:25 | 68.18 | 24.7 | 68 | 25.2 | 68 | 25.2 |
| 2/27/2025 8:26 | 68.18 | 24.8 | 68 | 25.3 | 68 | 25.3 |
| 2/27/2025 8:27 | 68.18 | 24.7 | 68 | 25.3 | 68 | 25.2 |
| 2/27/2025 8:28 | 68.18 | 24.8 | 68 | 25.2 | 68 | 25.2 |
| 2/27/2025 8:29 | 68.18 | 24.7 | 68 | 25.3 | 68 | 25.2 |
| 2/27/2025 8:30 | 68.18 | 24.7 | 68 | 25.2 | 68 | 25.3 |
| 2/27/2025 8:31 | 68.18 | 24.7 | 68 | 25.2 | 68 | 25.3 |
| 2/27/2025 8:32 | 68.18 | 24.7 | 68 | 25.2 | 68 | 25.2 |
| 2/27/2025 8:33 | 68.18 | 24.7 | 68.18 | 25.2 | 68 | 25.2 |
| 2/27/2025 8:34 | 68.18 | 24.7 | 68 | 25.2 | 68 | 25.2 |
| 2/27/2025 8:35 | 68.18 | 24.7 | 68 | 25.2 | 68 | 25.2 |
| 2/27/2025 8:36 | 68.18 | 24.7 | 68 | 25.2 | 68 | 25.2 |
| 2/27/2025 8:37 | 68.18 | 24.7 | 68 | 25.2 | 68 | 25.2 |
| 2/27/2025 8:38 | 68.18 | 24.7 | 68 | 25.2 | 68.18 | 25.2 |
| 2/27/2025 8:39 | 68.18 | 24.7 | 68 | 25.2 | 68 | 25.2 |
| 2/27/2025 8:40 | 68.18 | 24.7 | 68 | 25.2 | 68.18 | 25.2 |
| 2/27/2025 8:41 | 68.18 | 24.7 | 68 | 25.2 | 68 | 25.2 |
| 2/27/2025 8:42 | 68.18 | 24.7 | 68 | 25.2 | 68 | 25.2 |
| 2/27/2025 8:43 | 68.18 | 24.7 | 68 | 25.2 | 68.18 | 25.2 |
| 2/27/2025 8:44 | 68.36 | 24.7 | 68.18 | 25.2 | 68.18 | 25.3 |
| 2/27/2025 8:45 | 68.18 | 24.7 | 68.18 | 25.2 | 68 | 25.2 |
| 2/27/2025 8:46 | 68.18 | 24.7 | 68.18 | 25.2 | 68 | 25.2 |
| 2/27/2025 8:47 | 68.18 | 24.7 | 68 | 25.2 | 68.18 | 25.2 |
| 2/27/2025 8:48 | 68.36 | 24.6 | 68.18 | 25.2 | 68 | 25.2 |
| 2/27/2025 8:49 | 68.18 | 24.6 | 68.18 | 25.2 | 68.18 | 25.2 |
| 2/27/2025 8:50 | 68.18 | 24.6 | 68 | 25.2 | 68.18 | 25.2 |
| 2/27/2025 8:51 | 68.18 | 24.7 | 68.18 | 25.2 | 68 | 25.2 |
| 2/27/2025 8:52 | 68.18 | 24.6 | 68 | 25.2 | 68.18 | 25.2 |
| 2/27/2025 8:53 | 68.18 | 24.7 | 68.18 | 25.2 | 68 | 25.2 |
| 2/27/2025 8:54 | 68.36 | 24.7 | 68.18 | 25.2 | 68.18 | 25.2 |
| 2/27/2025 8:55 | 68.36 | 24.7 | 68.18 | 25.2 | 68.18 | 25.2 |
| 2/27/2025 8:56 | 68.36 | 24.7 | 68.18 | 25.2 | 68 | 25.2 |
| 2/27/2025 8:57 | 68.18 | 24.7 | 68.18 | 25.2 | 68 | 25.2 |
| 2/27/2025 8:58 | 68.36 | 24.7 | 68.18 | 25.2 | 68.18 | 25.2 |
| 2/27/2025 8:59 | 68.18 | 24.7 | 68.18 | 25.2 | 68.18 | 25.2 |
| 2/27/2025 9:00 | 68.18 | 24.7 | 68.18 | 25.2 | 68.18 | 25.2 |
| 2/27/2025 9:01 | 68.36 | 24.7 | 68 | 25.2 | 68.18 | 25.2 |
| 2/27/2025 9:02 | 68.18 | 24.7 | 68.18 | 25.2 | 68 | 25.2 |
| 2/27/2025 9:03 | 68.36 | 24.7 | 68.18 | 25.2 | 68.18 | 25.2 |
| 2/27/2025 9:04 | 68.18 | 24.7 | 68.18 | 25.2 | 68.18 | 25.2 |
| 2/27/2025 9:05 | 68.18 | 24.7 | 68.18 | 25.2 | 68.18 | 25.2 |
| 2/27/2025 9:06 | 68.36 | 24.7 | 68.18 | 25.2 | 68.18 | 25.2 |
| 2/27/2025 9:07 | 68.18 | 24.7 | 68.18 | 25.2 | 68.18 | 25.2 |
| 2/27/2025 9:08 | 68.18 | 24.7 | 68.18 | 25.2 | 68.18 | 25.2 |
| 2/27/2025 9:09 | 68.18 | 24.7 | 68.18 | 25.2 | 68.18 | 25.2 |
| 2/27/2025 9:10 | 68.18 | 24.7 | 68.18 | 25.2 | 68.18 | 25.2 |
| 2/27/2025 9:11 | 68.18 | 24.7 | 68.18 | 25.2 | 68.18 | 25.2 |
| 2/27/2025 9:12 | 68.18 | 24.7 | 68.18 | 25.2 | 68.18 | 25.2 |
| 2/27/2025 9:13 | 68.18 | 24.7 | 68.18 | 25.2 | 68.18 | 25.2 |
| 2/27/2025 9:14 | 68.36 | 24.7 | 68.18 | 25.2 | 68.18 | 25.2 |
| 2/27/2025 9:15 | 68.36 | 24.7 | 68.18 | 25.2 | 68.18 | 25.2 |
| 2/27/2025 9:16 | 68.36 | 24.7 | 68.18 | 25.2 | 68.18 | 25.2 |
| 2/27/2025 9:17 | 68.36 | 24.7 | 68 | 25.3 | 68.18 | 25.2 |
| 2/27/2025 9:18 | 68.36 | 24.7 | 68.18 | 25.2 | 68.18 | 25.2 |
| 2/27/2025 9:19 | 68.18 | 24.7 | 68.18 | 25.2 | 68.18 | 25.2 |
| 2/27/2025 9:20 | 68.36 | 24.7 | 68.18 | 25.2 | 68.18 | 25.2 |
| 2/27/2025 9:21 | 68.36 | 24.7 | 68.18 | 25.2 | 68.18 | 25.2 |
| 2/27/2025 9:22 | 68.36 | 24.7 | 68.18 | 25.2 | 68.18 | 25.2 |
| 2/27/2025 9:23 | 68.36 | 24.7 | 68.18 | 25.2 | 68.18 | 25.2 |
| 2/27/2025 9:24 | 68.36 | 24.7 | 68.18 | 25.2 | 68.18 | 25.2 |
| 2/27/2025 9:25 | 68.18 | 24.7 | 68.18 | 25.2 | 68.18 | 25.2 |
| 2/27/2025 9:26 | 68.18 | 24.7 | 68.18 | 25.2 | 68.18 | 25.2 |
| 2/27/2025 9:27 | 68.36 | 24.7 | 68.18 | 25.2 | 68.18 | 25.2 |
| 2/27/2025 9:28 | 68.36 | 24.7 | 68.18 | 25.2 | 68.18 | 25.2 |
| 2/27/2025 9:29 | 68.36 | 24.7 | 68.18 | 25.2 | 68.18 | 25.2 |
| 2/27/2025 9:30 | 68.36 | 24.7 | 68.18 | 25.2 | 68.18 | 25.2 |
| 2/27/2025 9:31 | 68.36 | 24.8 | 68.18 | 25.3 | 68.18 | 25.3 |
| 2/27/2025 9:32 | 68.36 | 25.1 | 68.18 | 25.5 | 68.18 | 25.6 |
| 2/27/2025 9:33 | 68.18 | 24.8 | 68.18 | 25.3 | 68.18 | 25.4 |
| 2/27/2025 9:34 | 68.18 | 24.8 | 68.18 | 25.3 | 68 | 25.4 |
| 2/27/2025 9:35 | 68.18 | 24.9 | 68.18 | 25.4 | 68 | 25.3 |
| 2/27/2025 9:36 | 68.18 | 24.9 | 68 | 25.3 | 68 | 25.3 |
| 2/27/2025 9:37 | 68.18 | 24.9 | 68 | 25.3 | 68 | 25.4 |
| 2/27/2025 9:38 | 68.18 | 24.9 | 68 | 25.3 | 68 | 25.4 |
| 2/27/2025 9:39 | 68.18 | 28.1 | 68 | 26.4 | 68 | 32.6 |
| 2/27/2025 9:40 | 68.9 | 33.8 | 69.26 | 36.3 | 69.98 | 40.3 |
| 2/27/2025 9:41 | 69.44 | 40.8 | 69.62 | 43.6 | 70.52 | 46.1 |
| 2/27/2025 9:42 | 69.62 | 44.8 | 69.62 | 48.7 | 70.52 | 50.5 |
| 2/27/2025 9:43 | 70.34 | 51.2 | 69.8 | 52.8 | 70.34 | 52.7 |
| 2/27/2025 9:44 | 70.52 | 54.4 | 70.16 | 55.3 | 70.52 | 51.7 |
| 2/27/2025 9:45 | 70.7 | 57.2 | 70.16 | 57.6 | 70.52 | 54.4 |
| 2/27/2025 9:46 | 70.7 | 59.6 | 70.16 | 59 | 70.52 | 56.3 |
| 2/27/2025 9:47 | 70.7 | 61.7 | 70.16 | 60.9 | 70.34 | 58.3 |
| 2/27/2025 9:48 | 70.7 | 63.4 | 70.16 | 62.2 | 70.52 | 59.9 |
| 2/27/2025 9:49 | 70.7 | 64.9 | 70.34 | 63.4 | 70.34 | 61.3 |
| 2/27/2025 9:50 | 70.7 | 66.3 | 70.34 | 64.5 | 70.52 | 62.5 |
| 2/27/2025 9:51 | 70.7 | 67.5 | 70.34 | 65.6 | 70.52 | 63.7 |
| 2/27/2025 9:52 | 70.7 | 68.5 | 70.34 | 66.5 | 70.52 | 64.7 |
| 2/27/2025 9:53 | 70.7 | 69.6 | 70.52 | 67.4 | 70.7 | 65.7 |
| 2/27/2025 9:54 | 70.7 | 70.4 | 70.52 | 68.3 | 70.7 | 66.7 |
| 2/27/2025 9:55 | 70.88 | 71.3 | 70.52 | 69 | 70.7 | 67.4 |
| 2/27/2025 9:56 | 70.88 | 72.2 | 70.52 | 69.8 | 70.7 | 68.3 |
| 2/27/2025 9:57 | 70.88 | 72.9 | 70.7 | 70.5 | 70.7 | 69 |
| 2/27/2025 9:58 | 70.88 | 73.5 | 70.7 | 71.2 | 70.7 | 69.7 |
| 2/27/2025 9:59 | 70.88 | 74.2 | 70.7 | 71.9 | 70.7 | 70.4 |
| 2/27/2025 10:00 | 70.88 | 74.7 | 70.7 | 72.5 | 70.88 | 71.1 |
| 2/27/2025 10:01 | 70.88 | 75.2 | 70.7 | 73.2 | 70.88 | 71.7 |
| 2/27/2025 10:02 | 70.88 | 75.7 | 70.88 | 73.7 | 70.88 | 72.3 |
| 2/27/2025 10:03 | 70.88 | 76.2 | 70.88 | 74.2 | 70.88 | 72.9 |
| 2/27/2025 10:04 | 70.88 | 76.7 | 70.88 | 74.7 | 70.88 | 73.4 |
| 2/27/2025 10:05 | 70.88 | 77.1 | 70.7 | 75.2 | 70.88 | 73.9 |
| 2/27/2025 10:06 | 70.7 | 77.5 | 70.88 | 75.6 | 70.88 | 74.4 |
| 2/27/2025 10:07 | 70.7 | 77.9 | 70.88 | 76.1 | 70.88 | 74.9 |
| 2/27/2025 10:08 | 70.88 | 78.4 | 70.88 | 76.5 | 70.88 | 75.3 |
| 2/27/2025 10:09 | 70.7 | 78.7 | 70.88 | 76.9 | 70.88 | 75.8 |
| 2/27/2025 10:10 | 70.7 | 79 | 70.88 | 77.4 | 70.88 | 76.2 |
| 2/27/2025 10:11 | 70.7 | 79.5 | 70.88 | 77.7 | 70.88 | 76.6 |
| 2/27/2025 10:12 | 70.7 | 79.8 | 70.88 | 78.1 | 70.88 | 77 |
| 2/27/2025 10:13 | 70.7 | 80.1 | 70.88 | 78.4 | 70.88 | 77.3 |
| 2/27/2025 10:14 | 70.7 | 80.4 | 70.88 | 78.7 | 70.88 | 77.7 |
| 2/27/2025 10:15 | 70.7 | 80.7 | 70.88 | 79 | 70.88 | 78 |
| 2/27/2025 10:16 | 70.7 | 81 | 70.88 | 79.3 | 70.88 | 78.3 |
| 2/27/2025 10:17 | 70.7 | 81.3 | 70.88 | 79.5 | 70.88 | 78.6 |
| 2/27/2025 10:18 | 70.7 | 81.5 | 70.88 | 79.9 | 70.88 | 78.8 |
| 2/27/2025 10:19 | 70.7 | 81.8 | 70.88 | 80.1 | 70.88 | 79.1 |
| 2/27/2025 10:20 | 70.7 | 82 | 70.88 | 80.3 | 70.88 | 79.4 |
| 2/27/2025 10:21 | 70.52 | 82.3 | 70.88 | 80.6 | 70.88 | 79.7 |
| 2/27/2025 10:22 | 70.7 | 82.5 | 70.88 | 80.8 | 70.88 | 79.9 |
| 2/27/2025 10:23 | 70.52 | 82.7 | 70.88 | 81.1 | 70.88 | 80.2 |
| 2/27/2025 10:24 | 70.52 | 83 | 70.88 | 81.3 | 70.88 | 80.4 |
| 2/27/2025 10:25 | 70.52 | 83.2 | 70.88 | 81.6 | 70.88 | 80.7 |
| 2/27/2025 10:26 | 70.52 | 83.4 | 70.88 | 81.7 | 70.88 | 80.9 |
| 2/27/2025 10:27 | 70.52 | 83.6 | 70.88 | 81.9 | 70.88 | 81.1 |
| 2/27/2025 10:28 | 70.52 | 83.7 | 70.88 | 82.1 | 70.88 | 81.3 |
| 2/27/2025 10:29 | 70.52 | 83.9 | 70.88 | 82.3 | 70.88 | 81.5 |
| 2/27/2025 10:30 | 70.52 | 84.1 | 70.88 | 82.5 | 70.88 | 81.6 |
| 2/27/2025 10:31 | 70.52 | 84.3 | 70.88 | 82.7 | 70.88 | 81.9 |
| 2/27/2025 10:32 | 70.52 | 84.5 | 70.88 | 82.8 | 70.88 | 82.1 |
| 2/27/2025 10:33 | 70.52 | 84.6 | 70.88 | 83 | 70.88 | 82.2 |
| 2/27/2025 10:34 | 70.52 | 84.7 | 70.88 | 83.2 | 70.88 | 82.4 |
| 2/27/2025 10:35 | 70.52 | 84.9 | 70.88 | 83.3 | 70.88 | 82.6 |
| 2/27/2025 10:36 | 70.52 | 85.1 | 70.88 | 83.5 | 70.88 | 82.7 |
| 2/27/2025 10:37 | 70.52 | 85.2 | 70.88 | 83.7 | 70.88 | 82.9 |
| 2/27/2025 10:38 | 70.52 | 85.4 | 70.88 | 83.8 | 70.88 | 83.1 |
| 2/27/2025 10:39 | 70.52 | 85.6 | 70.88 | 84 | 70.88 | 83.2 |
| 2/27/2025 10:40 | 70.52 | 85.7 | 70.88 | 84.1 | 70.88 | 83.4 |
| 2/27/2025 10:41 | 70.52 | 85.8 | 70.88 | 84.3 | 70.88 | 83.5 |
| 2/27/2025 10:42 | 70.52 | 86 | 70.88 | 84.4 | 70.88 | 83.7 |
| 2/27/2025 10:43 | 70.52 | 86.1 | 70.88 | 84.5 | 70.88 | 83.8 |
| 2/27/2025 10:44 | 70.7 | 86.2 | 70.88 | 84.7 | 70.88 | 83.9 |
| 2/27/2025 10:45 | 70.52 | 86.3 | 70.88 | 84.8 | 70.88 | 84 |
| 2/27/2025 10:46 | 70.7 | 86.5 | 70.88 | 84.9 | 70.88 | 84.1 |
| 2/27/2025 10:47 | 70.7 | 86.6 | 70.88 | 85 | 70.88 | 84.3 |
| 2/27/2025 10:48 | 70.52 | 86.7 | 70.88 | 85.2 | 70.88 | 84.4 |
| 2/27/2025 10:49 | 70.52 | 86.8 | 70.88 | 85.3 | 70.88 | 84.5 |
| 2/27/2025 10:50 | 70.52 | 86.9 | 70.88 | 85.4 | 70.88 | 84.6 |
| 2/27/2025 10:51 | 70.52 | 87 | 70.88 | 85.5 | 71.06 | 84.7 |
| 2/27/2025 10:52 | 70.52 | 87.2 | 70.88 | 85.7 | 71.06 | 84.8 |
| 2/27/2025 10:53 | 70.52 | 87.3 | 70.88 | 85.8 | 71.06 | 84.9 |
| 2/27/2025 10:54 | 70.52 | 87.4 | 70.88 | 85.9 | 70.88 | 85.1 |
| 2/27/2025 10:55 | 70.52 | 87.5 | 70.88 | 86 | 71.06 | 85.2 |
| 2/27/2025 10:56 | 70.7 | 87.6 | 70.88 | 86.2 | 71.06 | 85.3 |
| 2/27/2025 10:57 | 70.7 | 87.7 | 70.88 | 86.3 | 71.06 | 85.4 |
| 2/27/2025 10:58 | 70.52 | 87.8 | 70.88 | 86.4 | 70.88 | 85.5 |
| 2/27/2025 10:59 | 70.52 | 87.9 | 70.88 | 86.5 | 71.06 | 85.7 |
| 2/27/2025 11:00 | 70.7 | 88 | 70.88 | 86.6 | 71.06 | 85.8 |
| 2/27/2025 11:01 | 70.52 | 88.1 | 71.06 | 86.7 | 71.06 | 85.8 |
| 2/27/2025 11:02 | 70.52 | 88.2 | 70.88 | 86.8 | 71.06 | 85.9 |
| 2/27/2025 11:03 | 70.7 | 88.2 | 71.06 | 86.9 | 71.06 | 86.1 |
| 2/27/2025 11:04 | 70.7 | 88.3 | 70.88 | 87 | 71.06 | 86.2 |
| 2/27/2025 11:05 | 70.52 | 88.4 | 70.88 | 87.1 | 71.06 | 86.2 |
| 2/27/2025 11:06 | 70.7 | 88.5 | 71.06 | 87.2 | 71.06 | 86.3 |
| 2/27/2025 11:07 | 70.7 | 88.6 | 70.88 | 87.3 | 71.06 | 86.4 |
| 2/27/2025 11:08 | 70.7 | 88.7 | 70.88 | 87.4 | 71.06 | 86.5 |
| 2/27/2025 11:09 | 70.7 | 88.8 | 70.88 | 87.5 | 71.06 | 86.6 |
| 2/27/2025 11:10 | 70.7 | 88.9 | 70.88 | 87.6 | 71.06 | 86.7 |
| 2/27/2025 11:11 | 70.7 | 89 | 71.06 | 87.7 | 71.06 | 86.9 |
| 2/27/2025 11:12 | 70.7 | 89.1 | 71.06 | 87.8 | 71.06 | 87 |
| 2/27/2025 11:13 | 70.7 | 89.2 | 71.06 | 88 | 71.06 | 87.1 |
| 2/27/2025 11:14 | 70.7 | 89.3 | 71.06 | 88 | 71.06 | 87.2 |
| 2/27/2025 11:15 | 70.7 | 89.3 | 71.06 | 88.1 | 71.06 | 87.2 |
| 2/27/2025 11:16 | 70.7 | 89.4 | 71.06 | 88.2 | 71.06 | 87.4 |
| 2/27/2025 11:17 | 70.7 | 89.5 | 71.06 | 88.3 | 71.06 | 87.4 |
| 2/27/2025 11:18 | 70.7 | 89.5 | 71.06 | 88.3 | 71.06 | 87.5 |
| 2/27/2025 11:19 | 70.7 | 89.6 | 71.06 | 88.4 | 71.06 | 87.6 |
| 2/27/2025 11:20 | 70.7 | 89.7 | 71.06 | 88.5 | 71.06 | 87.7 |
| 2/27/2025 11:21 | 70.7 | 89.7 | 71.06 | 88.6 | 71.06 | 87.8 |
| 2/27/2025 11:22 | 70.7 | 89.8 | 71.06 | 88.6 | 71.06 | 87.8 |
| 2/27/2025 11:23 | 70.7 | 89.9 | 71.06 | 88.7 | 71.24 | 87.9 |
| 2/27/2025 11:24 | 70.88 | 89.9 | 71.06 | 88.8 | 71.06 | 87.9 |
| 2/27/2025 11:25 | 70.7 | 90 | 71.06 | 88.9 | 71.24 | 88.1 |
| 2/27/2025 11:26 | 70.88 | 90.1 | 71.06 | 88.9 | 71.06 | 88.1 |
| 2/27/2025 11:27 | 70.88 | 90.1 | 71.06 | 89 | 71.24 | 88.2 |
| 2/27/2025 11:28 | 70.88 | 90.2 | 71.06 | 89.1 | 71.24 | 88.3 |
| 2/27/2025 11:29 | 70.88 | 90.2 | 71.24 | 89.2 | 71.24 | 88.3 |
| 2/27/2025 11:30 | 70.88 | 90.3 | 71.24 | 89.2 | 71.24 | 88.4 |
| 2/27/2025 11:31 | 70.88 | 90.3 | 71.24 | 89.3 | 71.24 | 88.5 |
| 2/27/2025 11:32 | 70.88 | 90.4 | 71.24 | 89.3 | 71.24 | 88.5 |
| 2/27/2025 11:33 | 70.88 | 90.5 | 71.24 | 89.4 | 71.24 | 88.6 |
| 2/27/2025 11:34 | 70.88 | 90.5 | 71.24 | 89.5 | 71.24 | 88.6 |
| 2/27/2025 11:35 | 70.88 | 90.5 | 71.24 | 89.5 | 71.24 | 88.7 |
| 2/27/2025 11:36 | 70.88 | 90.5 | 71.24 | 89.6 | 71.24 | 88.8 |
| 2/27/2025 11:37 | 71.06 | 90.6 | 71.24 | 89.6 | 71.24 | 88.8 |
| 2/27/2025 11:38 | 70.88 | 90.7 | 71.24 | 89.7 | 71.24 | 88.9 |
| 2/27/2025 11:39 | 70.88 | 90.7 | 71.24 | 89.7 | 71.24 | 88.9 |
| 2/27/2025 11:40 | 70.88 | 90.7 | 71.24 | 89.8 | 71.24 | 89 |
| 2/27/2025 11:41 | 71.06 | 90.8 | 71.24 | 89.8 | 71.24 | 89 |
| 2/27/2025 11:42 | 70.88 | 90.8 | 71.24 | 89.9 | 71.24 | 89 |
| 2/27/2025 11:43 | 71.06 | 90.8 | 71.24 | 89.9 | 71.24 | 89 |
| 2/27/2025 11:44 | 71.06 | 90.9 | 71.24 | 89.9 | 71.24 | 89.1 |
| 2/27/2025 11:45 | 71.06 | 90.9 | 71.24 | 90 | 71.24 | 89.1 |
| 2/27/2025 11:46 | 70.88 | 91 | 71.24 | 90 | 71.24 | 89.2 |
| 2/27/2025 11:47 | 70.88 | 91 | 71.24 | 90 | 71.24 | 89.2 |
| 2/27/2025 11:48 | 71.06 | 91 | 71.24 | 90.1 | 71.24 | 89.3 |
| 2/27/2025 11:49 | 71.06 | 91.1 | 71.24 | 90.2 | 71.24 | 89.3 |
| 2/27/2025 11:50 | 70.88 | 91.1 | 71.24 | 90.2 | 71.24 | 89.4 |
| 2/27/2025 11:51 | 71.06 | 91.2 | 71.24 | 90.2 | 71.24 | 89.4 |
| 2/27/2025 11:52 | 71.06 | 91.2 | 71.24 | 90.2 | 71.24 | 89.5 |
| 2/27/2025 11:53 | 71.06 | 91.3 | 71.24 | 90.3 | 71.24 | 89.5 |
| 2/27/2025 11:54 | 71.06 | 91.3 | 71.24 | 90.3 | 71.24 | 89.6 |
| 2/27/2025 11:55 | 71.06 | 91.3 | 71.24 | 90.4 | 71.24 | 89.6 |
| 2/27/2025 11:56 | 70.88 | 91.3 | 71.24 | 90.4 | 71.24 | 89.6 |
| 2/27/2025 11:57 | 70.88 | 91.4 | 71.24 | 90.4 | 71.24 | 89.6 |
| 2/27/2025 11:58 | 70.88 | 91.4 | 71.24 | 90.5 | 71.24 | 89.7 |
| 2/27/2025 11:59 | 70.88 | 91.5 | 71.24 | 90.5 | 71.24 | 89.7 |
| 2/27/2025 12:00 | 70.88 | 91.6 | 71.24 | 90.6 | 71.24 | 89.8 |
| 2/27/2025 12:01 | 70.88 | 91.6 | 71.24 | 90.7 | 71.24 | 89.8 |
| 2/27/2025 12:02 | 70.88 | 91.7 | 71.24 | 90.7 | 71.24 | 89.9 |
| 2/27/2025 12:03 | 70.88 | 91.7 | 71.24 | 90.8 | 71.24 | 89.9 |
| 2/27/2025 12:04 | 70.88 | 91.8 | 71.24 | 90.8 | 71.42 | 89.9 |
| 2/27/2025 12:05 | 70.88 | 91.9 | 71.24 | 90.9 | 71.42 | 89.9 |
| 2/27/2025 12:06 | 70.88 | 92 | 71.24 | 91 | 71.24 | 90 |
| 2/27/2025 12:07 | 70.88 | 92.1 | 71.24 | 91 | 71.24 | 90.1 |
| 2/27/2025 12:08 | 70.88 | 92.1 | 71.24 | 91.1 | 71.24 | 90.2 |
| 2/27/2025 12:09 | 70.88 | 92.1 | 71.24 | 91.1 | 71.42 | 90.3 |
| 2/27/2025 12:10 | 71.06 | 92.1 | 71.24 | 91.1 | 71.24 | 90.3 |
| 2/27/2025 12:11 | 71.06 | 92.2 | 71.24 | 91.2 | 71.42 | 90.3 |
| 2/27/2025 12:12 | 70.88 | 92.2 | 71.24 | 91.2 | 71.42 | 90.4 |
| 2/27/2025 12:13 | 71.06 | 92.2 | 71.24 | 91.2 | 71.42 | 90.4 |
| 2/27/2025 12:14 | 71.06 | 92.3 | 71.24 | 91.3 | 71.42 | 90.4 |
| 2/27/2025 12:15 | 70.88 | 92.3 | 71.42 | 91.3 | 71.42 | 90.5 |
| 2/27/2025 12:16 | 71.06 | 92.3 | 71.24 | 91.3 | 71.24 | 90.5 |
| 2/27/2025 12:17 | 70.88 | 92.3 | 71.24 | 91.4 | 71.42 | 90.5 |
| 2/27/2025 12:18 | 71.06 | 92.4 | 71.24 | 91.4 | 71.42 | 90.5 |
| 2/27/2025 12:19 | 70.88 | 92.4 | 71.24 | 91.4 | 71.42 | 90.5 |
| 2/27/2025 12:20 | 71.06 | 92.4 | 71.24 | 91.4 | 71.42 | 90.5 |
| 2/27/2025 12:21 | 70.88 | 92.4 | 71.24 | 91.5 | 71.42 | 90.5 |
| 2/27/2025 12:22 | 70.88 | 92.5 | 71.24 | 91.5 | 71.42 | 90.6 |
| 2/27/2025 12:23 | 70.88 | 92.5 | 71.24 | 91.5 | 71.42 | 90.6 |
| 2/27/2025 12:24 | 70.88 | 92.5 | 71.24 | 91.6 | 71.42 | 90.6 |
| 2/27/2025 12:25 | 70.88 | 92.6 | 71.24 | 91.6 | 71.42 | 90.7 |
| 2/27/2025 12:26 | 70.88 | 92.6 | 71.24 | 91.7 | 71.42 | 90.7 |
| 2/27/2025 12:27 | 70.88 | 92.7 | 71.24 | 91.7 | 71.42 | 90.7 |
| 2/27/2025 12:28 | 70.88 | 92.7 | 71.24 | 91.8 | 71.24 | 90.8 |
| 2/27/2025 12:29 | 70.88 | 92.8 | 71.24 | 91.8 | 71.24 | 90.8 |
| 2/27/2025 12:30 | 70.88 | 92.8 | 71.24 | 91.9 | 71.24 | 90.9 |
| 2/27/2025 12:31 | 70.88 | 92.8 | 71.24 | 91.9 | 71.24 | 90.9 |
| 2/27/2025 12:32 | 70.88 | 92.9 | 71.06 | 92 | 71.24 | 91 |
| 2/27/2025 12:33 | 70.88 | 92.9 | 71.24 | 92.1 | 71.24 | 91 |
| 2/27/2025 12:34 | 70.88 | 93 | 71.24 | 92.1 | 71.24 | 91.1 |
| 2/27/2025 12:35 | 70.88 | 93 | 71.06 | 92.1 | 71.24 | 91.1 |
| 2/27/2025 12:36 | 70.88 | 93.1 | 71.24 | 92.2 | 71.24 | 91.2 |
| 2/27/2025 12:37 | 70.88 | 93.1 | 71.06 | 92.2 | 71.24 | 91.2 |
| 2/27/2025 12:38 | 70.88 | 93.2 | 71.24 | 92.3 | 71.24 | 91.2 |
| 2/27/2025 12:39 | 70.7 | 93.2 | 71.06 | 92.3 | 71.24 | 91.3 |
| 2/27/2025 12:40 | 70.88 | 93.3 | 71.06 | 92.4 | 71.24 | 91.3 |
| 2/27/2025 12:41 | 70.88 | 93.3 | 71.06 | 92.4 | 71.24 | 91.4 |
| 2/27/2025 12:42 | 70.88 | 93.3 | 71.06 | 92.5 | 71.24 | 91.4 |
| 2/27/2025 12:43 | 70.7 | 93.4 | 71.06 | 92.5 | 71.24 | 91.5 |
| 2/27/2025 12:44 | 70.7 | 93.4 | 71.06 | 92.5 | 71.24 | 91.5 |
| 2/27/2025 12:45 | 70.88 | 93.5 | 71.06 | 92.6 | 71.24 | 91.5 |
| 2/27/2025 12:46 | 70.7 | 93.5 | 71.06 | 92.6 | 71.24 | 91.6 |
| 2/27/2025 12:47 | 70.7 | 93.5 | 71.06 | 92.6 | 71.24 | 91.6 |
| 2/27/2025 12:48 | 70.7 | 93.6 | 71.06 | 92.7 | 71.24 | 91.7 |
| 2/27/2025 12:49 | 70.7 | 93.6 | 71.06 | 92.7 | 71.24 | 91.7 |
| 2/27/2025 12:50 | 70.7 | 93.7 | 71.06 | 92.8 | 71.24 | 91.8 |
| 2/27/2025 12:51 | 70.7 | 93.7 | 71.06 | 92.8 | 71.24 | 91.8 |
| 2/27/2025 12:52 | 70.7 | 93.7 | 71.06 | 92.9 | 71.24 | 91.8 |
| 2/27/2025 12:53 | 70.7 | 93.7 | 71.06 | 92.9 | 71.24 | 91.9 |
| 2/27/2025 12:54 | 70.88 | 93.8 | 71.06 | 92.9 | 71.06 | 91.9 |
| 2/27/2025 12:55 | 70.88 | 93.8 | 71.06 | 93 | 71.24 | 92 |
| 2/27/2025 12:56 | 70.7 | 93.9 | 71.06 | 93.1 | 71.24 | 92 |
| 2/27/2025 12:57 | 70.88 | 93.9 | 71.06 | 93.1 | 71.24 | 92 |
| 2/27/2025 12:58 | 70.88 | 93.9 | 71.06 | 93.1 | 71.24 | 92.1 |
| 2/27/2025 12:59 | 70.7 | 93.9 | 71.06 | 93.1 | 71.24 | 92.1 |
| 2/27/2025 13:00 | 70.7 | 94 | 71.06 | 93.1 | 71.24 | 92.2 |
| 2/27/2025 13:01 | 70.88 | 94 | 71.06 | 93.2 | 71.24 | 92.2 |
| 2/27/2025 13:02 | 70.7 | 94 | 71.06 | 93.2 | 71.24 | 92.2 |
| 2/27/2025 13:03 | 70.7 | 94 | 71.06 | 93.3 | 71.24 | 92.2 |
| 2/27/2025 13:04 | 70.7 | 94.1 | 71.06 | 93.3 | 71.06 | 92.3 |
| 2/27/2025 13:05 | 70.7 | 94.1 | 71.06 | 93.3 | 71.24 | 92.3 |
| 2/27/2025 13:06 | 70.7 | 94.1 | 71.06 | 93.3 | 71.24 | 92.3 |
| 2/27/2025 13:07 | 70.7 | 94.2 | 71.06 | 93.4 | 71.06 | 92.4 |
| 2/27/2025 13:08 | 70.7 | 94.2 | 71.06 | 93.4 | 71.24 | 92.4 |
| 2/27/2025 13:09 | 70.7 | 94.3 | 71.06 | 93.4 | 71.24 | 92.5 |
| 2/27/2025 13:10 | 70.7 | 94.3 | 71.06 | 93.5 | 71.24 | 92.5 |
| 2/27/2025 13:11 | 70.7 | 94.3 | 71.06 | 93.5 | 71.24 | 92.5 |
| 2/27/2025 13:12 | 70.7 | 94.3 | 71.06 | 93.6 | 71.24 | 92.6 |
| 2/27/2025 13:13 | 70.7 | 94.4 | 71.06 | 93.6 | 71.24 | 92.6 |
| 2/27/2025 13:14 | 70.7 | 94.4 | 71.06 | 93.7 | 71.24 | 92.7 |
| 2/27/2025 13:15 | 70.7 | 94.5 | 71.06 | 93.7 | 71.24 | 92.7 |
| 2/27/2025 13:16 | 70.7 | 94.5 | 71.06 | 93.7 | 71.06 | 92.8 |
| 2/27/2025 13:17 | 70.7 | 94.5 | 71.06 | 93.8 | 71.24 | 92.8 |
| 2/27/2025 13:18 | 70.7 | 94.6 | 71.06 | 93.8 | 71.24 | 92.8 |
| 2/27/2025 13:19 | 70.7 | 94.6 | 71.06 | 93.8 | 71.24 | 92.9 |
| 2/27/2025 13:20 | 70.7 | 94.6 | 71.06 | 93.9 | 71.24 | 92.9 |
| 2/27/2025 13:21 | 70.88 | 94.6 | 71.06 | 93.9 | 71.24 | 93 |
| 2/27/2025 13:22 | 70.88 | 94.7 | 71.24 | 94 | 71.24 | 93 |
| 2/27/2025 13:23 | 70.88 | 94.7 | 71.06 | 94 | 71.24 | 93 |
| 2/27/2025 13:24 | 70.88 | 94.8 | 71.06 | 94 | 71.24 | 93.1 |
| 2/27/2025 13:25 | 70.88 | 94.8 | 71.06 | 94 | 71.24 | 93.1 |
| 2/27/2025 13:26 | 70.88 | 94.8 | 71.06 | 94.1 | 71.24 | 93.1 |
| 2/27/2025 13:27 | 70.88 | 94.8 | 71.24 | 94.1 | 71.24 | 93.2 |
| 2/27/2025 13:28 | 70.88 | 94.9 | 71.24 | 94.1 | 71.24 | 93.2 |
| 2/27/2025 13:29 | 70.88 | 94.9 | 71.24 | 94.1 | 71.24 | 93.2 |
| 2/27/2025 13:30 | 70.88 | 94.9 | 71.24 | 94.1 | 71.24 | 93.2 |
| 2/27/2025 13:31 | 70.88 | 94.9 | 71.24 | 94.2 | 71.24 | 93.2 |
| 2/27/2025 13:32 | 70.88 | 94.9 | 71.24 | 94.2 | 71.24 | 93.3 |
| 2/27/2025 13:33 | 70.88 | 94.9 | 71.24 | 94.2 | 71.24 | 93.3 |
| 2/27/2025 13:34 | 70.88 | 95 | 71.24 | 94.2 | 71.24 | 93.3 |
| 2/27/2025 13:35 | 70.88 | 95 | 71.24 | 94.3 | 71.24 | 93.3 |
| 2/27/2025 13:36 | 70.88 | 95 | 71.24 | 94.3 | 71.42 | 93.3 |
| 2/27/2025 13:37 | 70.88 | 95.1 | 71.24 | 94.3 | 71.24 | 93.4 |
| 2/27/2025 13:38 | 70.88 | 95.1 | 71.24 | 94.4 | 71.42 | 93.4 |
| 2/27/2025 13:39 | 71.06 | 95.1 | 71.24 | 94.4 | 71.24 | 93.4 |
| 2/27/2025 13:40 | 71.06 | 95.2 | 71.24 | 94.4 | 71.42 | 93.5 |
| 2/27/2025 13:41 | 71.06 | 95.2 | 71.24 | 94.5 | 71.42 | 93.5 |
| 2/27/2025 13:42 | 71.06 | 95.3 | 71.24 | 94.5 | 71.42 | 93.6 |
| 2/27/2025 13:43 | 71.06 | 95.4 | 71.42 | 94.6 | 71.42 | 93.7 |
| 2/27/2025 13:44 | 71.06 | 95.4 | 71.42 | 94.6 | 71.42 | 93.7 |
| 2/27/2025 13:45 | 71.06 | 95.5 | 71.42 | 94.7 | 71.6 | 93.7 |
| 2/27/2025 13:46 | 71.24 | 95.5 | 71.42 | 94.7 | 71.6 | 93.7 |
| 2/27/2025 13:47 | 71.24 | 95.5 | 71.42 | 94.7 | 71.6 | 93.7 |
| 2/27/2025 13:48 | 71.24 | 95.4 | 71.42 | 94.6 | 71.6 | 93.6 |
| 2/27/2025 13:49 | 71.24 | 95.2 | 71.42 | 94.4 | 71.6 | 93.5 |
| 2/27/2025 13:50 | 71.06 | 95.1 | 71.42 | 94.3 | 71.6 | 93.4 |
| 2/27/2025 13:51 | 71.06 | 95 | 71.42 | 94.2 | 71.6 | 93.3 |
| 2/27/2025 13:52 | 71.06 | 94.9 | 71.42 | 94 | 71.6 | 93.1 |
| 2/27/2025 13:53 | 71.06 | 94.8 | 71.42 | 94 | 71.6 | 93.1 |
| 2/27/2025 13:54 | 71.06 | 94.8 | 71.42 | 93.9 | 71.42 | 93 |
| 2/27/2025 13:55 | 71.06 | 94.7 | 71.24 | 93.8 | 71.42 | 93 |
| 2/27/2025 13:56 | 71.06 | 94.7 | 71.24 | 93.8 | 71.42 | 93.1 |
| 2/27/2025 13:57 | 71.06 | 94.6 | 71.24 | 93.8 | 71.42 | 93.1 |
| 2/27/2025 13:58 | 70.88 | 94.6 | 71.24 | 93.8 | 71.42 | 93 |
| 2/27/2025 13:59 | 70.88 | 94.6 | 71.24 | 93.8 | 71.42 | 93.1 |
| 2/27/2025 14:00 | 70.88 | 94.6 | 71.24 | 93.7 | 71.42 | 93 |
| 2/27/2025 14:01 | 70.88 | 94.5 | 71.24 | 93.7 | 71.42 | 93 |
| 2/27/2025 14:02 | 70.88 | 94.5 | 71.24 | 93.7 | 71.42 | 93 |
| 2/27/2025 14:03 | 70.88 | 94.5 | 71.06 | 93.6 | 71.42 | 93 |
| 2/27/2025 14:04 | 70.88 | 94.5 | 71.06 | 93.6 | 71.42 | 92.9 |
| 2/27/2025 14:05 | 70.7 | 94.4 | 71.06 | 93.6 | 71.42 | 92.9 |
| 2/27/2025 14:06 | 70.7 | 94.4 | 71.06 | 93.6 | 71.42 | 92.9 |
| 2/27/2025 14:07 | 70.7 | 94.4 | 71.06 | 93.6 | 71.42 | 92.9 |
| 2/27/2025 14:08 | 70.7 | 94.3 | 71.06 | 93.5 | 71.42 | 92.9 |
| 2/27/2025 14:09 | 70.7 | 94.3 | 71.06 | 93.6 | 71.42 | 92.9 |
| 2/27/2025 14:10 | 70.7 | 94.3 | 71.06 | 93.6 | 71.42 | 92.9 |
| 2/27/2025 14:11 | 70.7 | 94.3 | 70.88 | 93.6 | 71.42 | 92.9 |
| 2/27/2025 14:12 | 70.7 | 94.3 | 70.88 | 93.6 | 71.42 | 92.9 |
| 2/27/2025 14:13 | 70.52 | 94.4 | 70.88 | 93.6 | 71.42 | 92.9 |
| 2/27/2025 14:14 | 70.52 | 94.3 | 70.88 | 93.7 | 71.42 | 92.9 |
| 2/27/2025 14:15 | 70.52 | 94.4 | 70.88 | 93.7 | 71.42 | 92.9 |
| 2/27/2025 14:16 | 70.52 | 94.4 | 70.88 | 93.8 | 71.42 | 92.9 |
| 2/27/2025 14:17 | 70.52 | 94.4 | 70.88 | 93.8 | 71.42 | 92.9 |
| 2/27/2025 14:18 | 70.52 | 94.4 | 70.88 | 93.8 | 71.6 | 92.9 |
| 2/27/2025 14:19 | 70.52 | 94.4 | 70.88 | 93.8 | 71.6 | 92.9 |
| 2/27/2025 14:20 | 70.52 | 94.4 | 70.88 | 93.8 | 71.6 | 92.9 |
| 2/27/2025 14:21 | 70.52 | 94.4 | 70.88 | 93.8 | 71.6 | 92.9 |
| 2/27/2025 14:22 | 70.52 | 94.5 | 70.88 | 93.8 | 71.6 | 92.8 |
| 2/27/2025 14:23 | 70.52 | 94.5 | 70.88 | 93.8 | 71.6 | 92.8 |
| 2/27/2025 14:24 | 70.52 | 94.5 | 70.88 | 93.9 | 71.6 | 92.8 |
| 2/27/2025 14:25 | 70.52 | 94.5 | 70.88 | 93.8 | 71.6 | 92.8 |
| 2/27/2025 14:26 | 70.52 | 94.5 | 70.88 | 93.9 | 71.6 | 92.8 |
| 2/27/2025 14:27 | 70.52 | 94.5 | 70.88 | 93.9 | 71.6 | 92.7 |
| 2/27/2025 14:28 | 70.52 | 94.5 | 70.88 | 93.8 | 71.6 | 92.8 |
| 2/27/2025 14:29 | 70.52 | 94.5 | 70.88 | 93.9 | 71.6 | 92.8 |
| 2/27/2025 14:30 | 70.52 | 94.5 | 70.88 | 93.9 | 71.6 | 92.7 |
| 2/27/2025 14:31 | 70.52 | 94.5 | 70.88 | 94 | 71.6 | 92.8 |
| 2/27/2025 14:32 | 70.52 | 94.5 | 70.88 | 94 | 71.6 | 92.8 |
| 2/27/2025 14:33 | 70.52 | 94.6 | 70.88 | 94 | 71.78 | 92.8 |
| 2/27/2025 14:34 | 70.52 | 94.5 | 70.88 | 94 | 71.78 | 92.8 |
| 2/27/2025 14:35 | 70.52 | 94.6 | 70.88 | 94 | 71.78 | 92.8 |
| 2/27/2025 14:36 | 70.52 | 94.5 | 70.88 | 94 | 71.78 | 92.8 |
| 2/27/2025 14:37 | 70.52 | 94.6 | 70.88 | 94 | 71.78 | 92.8 |
| 2/27/2025 14:38 | 70.52 | 94.6 | 70.88 | 94.1 | 71.78 | 92.8 |
| 2/27/2025 14:39 | 70.52 | 94.6 | 70.88 | 94.1 | 71.78 | 92.8 |
| 2/27/2025 14:40 | 70.52 | 94.6 | 70.88 | 94.1 | 71.78 | 92.8 |
| 2/27/2025 14:41 | 70.52 | 94.6 | 70.88 | 94.1 | 71.78 | 92.8 |
| 2/27/2025 14:42 | 70.52 | 94.6 | 70.88 | 94.1 | 71.78 | 92.8 |
| 2/27/2025 14:43 | 70.52 | 94.6 | 70.88 | 94.1 | 71.78 | 92.8 |
| 2/27/2025 14:44 | 70.52 | 94.6 | 70.88 | 94.1 | 71.78 | 92.8 |
| 2/27/2025 14:45 | 70.52 | 94.6 | 70.88 | 94.1 | 71.78 | 92.8 |
| 2/27/2025 14:46 | 70.52 | 94.6 | 70.88 | 94 | 71.96 | 92.8 |
| 2/27/2025 14:47 | 70.52 | 94.6 | 70.88 | 94 | 71.96 | 92.8 |
| 2/27/2025 14:48 | 70.52 | 94.6 | 70.88 | 94 | 71.96 | 92.8 |
| 2/27/2025 14:49 | 70.34 | 94.6 | 70.88 | 94.1 | 71.96 | 92.8 |
| 2/27/2025 14:50 | 70.52 | 94.6 | 70.88 | 94.1 | 71.96 | 92.8 |
| 2/27/2025 14:51 | 70.52 | 94.6 | 71.06 | 94.1 | 71.96 | 92.8 |
| 2/27/2025 14:52 | 70.52 | 94.6 | 70.88 | 94.1 | 71.96 | 92.8 |
| 2/27/2025 14:53 | 70.52 | 94.6 | 70.88 | 94.1 | 71.96 | 92.7 |
| 2/27/2025 14:54 | 70.34 | 94.6 | 70.88 | 94.1 | 71.96 | 92.7 |
| 2/27/2025 14:55 | 70.52 | 94.6 | 70.88 | 94.1 | 71.96 | 92.7 |
| 2/27/2025 14:56 | 70.34 | 94.6 | 70.88 | 94.1 | 71.96 | 92.7 |
| 2/27/2025 14:57 | 70.52 | 94.6 | 70.88 | 94.1 | 71.96 | 92.7 |
| 2/27/2025 14:58 | 70.34 | 94.6 | 70.88 | 94.1 | 71.96 | 92.7 |
| 2/27/2025 14:59 | 70.34 | 94.6 | 70.88 | 94.1 | 71.96 | 92.7 |
| 2/27/2025 15:00 | 70.34 | 94.6 | 70.88 | 94.1 | 71.96 | 92.7 |
| 2/27/2025 15:01 | 70.34 | 94.6 | 70.88 | 94.1 | 71.96 | 92.6 |
| 2/27/2025 15:02 | 70.34 | 94.5 | 70.88 | 94 | 71.96 | 92.6 |
| 2/27/2025 15:03 | 70.34 | 94.6 | 70.88 | 94 | 71.96 | 92.6 |
| 2/27/2025 15:04 | 70.34 | 94.6 | 70.88 | 94 | 71.78 | 92.6 |
| 2/27/2025 15:05 | 70.34 | 94.6 | 70.88 | 94 | 71.78 | 92.6 |
| 2/27/2025 15:06 | 70.34 | 94.6 | 70.88 | 94 | 71.78 | 92.6 |
| 2/27/2025 15:07 | 70.34 | 94.5 | 70.88 | 94 | 71.78 | 92.6 |
| 2/27/2025 15:08 | 70.34 | 94.5 | 70.88 | 94 | 71.78 | 92.6 |
| 2/27/2025 15:09 | 70.34 | 94.6 | 70.88 | 94.1 | 71.78 | 92.6 |
| 2/27/2025 15:10 | 70.34 | 94.6 | 70.88 | 94.1 | 71.78 | 92.7 |
| 2/27/2025 15:11 | 70.34 | 94.6 | 70.88 | 94.1 | 71.78 | 92.7 |
| 2/27/2025 15:12 | 70.34 | 94.5 | 70.7 | 94.1 | 71.78 | 92.7 |
| 2/27/2025 15:13 | 70.34 | 94.5 | 70.7 | 94.1 | 71.78 | 92.7 |
| 2/27/2025 15:14 | 70.34 | 94.5 | 70.88 | 94.1 | 71.78 | 92.7 |
| 2/27/2025 15:15 | 70.16 | 94.5 | 70.7 | 94.1 | 71.78 | 92.6 |
| 2/27/2025 15:16 | 70.16 | 94.5 | 70.7 | 94 | 72.68 | 47.8 |
| 2/27/2025 15:17 | 70.7 | 44.8 | 74.3 | 28.1 | 77 | 13.8 |
| 2/27/2025 15:18 | 71.78 | 35.7 | 75.74 | 24.5 | 78.62 | 13.4 |
| 2/27/2025 15:19 | 72.68 | 30.6 | 76.28 | 22.7 | 79.16 | 13.3 |
| 2/27/2025 15:20 | 73.04 | 27.8 | 76.46 | 21.4 | 78.98 | 13.3 |
| 2/27/2025 15:21 | 73.4 | 25.4 | 76.46 | 20.5 | 78.8 | 13.4 |
| 2/27/2025 15:22 | 73.58 | 23.8 | 76.28 | 19.6 | 78.44 | 13.5 |
| 2/27/2025 15:23 | 73.58 | 22.5 | 76.28 | 18.9 | 78.26 | 13.6 |
| 2/27/2025 15:24 | 73.58 | 21.5 | 76.1 | 18.2 | 78.08 | 13.7 |
| 2/27/2025 15:25 | 73.58 | 20.8 | 75.92 | 17.7 | 77.72 | 13.8 |
| 2/27/2025 15:26 | 73.58 | 19.9 | 75.74 | 17.2 | 77.54 | 13.9 |
| 2/27/2025 15:27 | 73.4 | 19.3 | 75.74 | 16.9 | 77.18 | 13.9 |
| 2/27/2025 15:28 | 73.4 | 18.7 | 75.56 | 16.4 | 76.82 | 14 |
| 2/27/2025 15:29 | 73.22 | 18.2 | 75.38 | 16 | 76.64 | 14.1 |
| 2/27/2025 15:30 | 73.22 | 17.8 | 75.2 | 15.6 | 76.46 | 14.1 |
| 2/27/2025 15:31 | 73.22 | 17.3 | 75.02 | 15.4 | 76.1 | 14.1 |
| 2/27/2025 15:32 | 73.04 | 16.9 | 75.02 | 15 | 75.92 | 14.2 |
| 2/27/2025 15:33 | 73.04 | 16.6 | 74.84 | 14.8 | 75.74 | 14.1 |
| 2/27/2025 15:34 | 72.86 | 16.3 | 74.66 | 14.5 | 75.56 | 14.2 |
| 2/27/2025 15:35 | 72.68 | 16 | 74.48 | 14.3 | 75.38 | 14.2 |
| 2/27/2025 15:36 | 72.68 | 15.7 | 74.48 | 14.1 | 75.2 | 14.2 |
| 2/27/2025 15:37 | 72.68 | 15.4 | 74.3 | 13.8 | 75.02 | 14.1 |
| 2/27/2025 15:38 | 72.5 | 15.1 | 74.12 | 13.6 | 74.84 | 14.1 |
| 2/27/2025 15:39 | 72.5 | 14.9 | 74.12 | 13.4 | 74.66 | 14.1 |
| 2/27/2025 15:40 | 72.5 | 14.7 | 73.94 | 13.2 | 74.48 | 14.1 |
| 2/27/2025 15:41 | 72.32 | 14.5 | 73.76 | 13.1 | 74.3 | 14 |
| 2/27/2025 15:42 | 72.32 | 14.3 | 73.76 | 12.9 | 74.12 | 14 |
| 2/27/2025 15:43 | 72.14 | 14.1 | 73.58 | 12.8 | 73.94 | 13.9 |
| 2/27/2025 15:44 | 72.32 | 13.9 | 73.58 | 12.6 | 73.94 | 13.9 |
| 2/27/2025 15:45 | 72.14 | 13.7 | 73.4 | 12.5 | 73.76 | 13.9 |
| 2/27/2025 15:46 | 72.14 | 13.6 | 73.4 | 12.3 | 73.58 | 13.8 |
| 2/27/2025 15:47 | 71.96 | 13.4 | 73.22 | 12.2 | 73.58 | 13.8 |
| 2/27/2025 15:48 | 71.96 | 13.3 | 73.22 | 12.1 | 73.4 | 13.7 |
| 2/27/2025 15:49 | 71.96 | 13.1 | 73.04 | 12 | 73.22 | 13.6 |
| 2/27/2025 15:50 | 71.78 | 13 | 72.86 | 11.8 | 73.22 | 13.6 |
| 2/27/2025 15:51 | 71.78 | 12.9 | 72.86 | 11.7 | 73.04 | 13.5 |
| 2/27/2025 15:52 | 71.78 | 12.7 | 72.86 | 11.6 | 73.04 | 13.4 |
| 2/27/2025 15:53 | 71.78 | 12.6 | 72.68 | 11.5 | 72.86 | 13.4 |
| 2/27/2025 15:54 | 71.6 | 12.5 | 72.68 | 11.5 | 72.86 | 13.3 |
| 2/27/2025 15:55 | 71.6 | 12.4 | 72.5 | 11.3 | 72.68 | 13.3 |
| 2/27/2025 15:56 | 71.6 | 12.2 | 72.5 | 11.3 | 72.68 | 13.2 |
| 2/27/2025 15:57 | 71.6 | 12.1 | 72.5 | 11.2 | 72.5 | 13.1 |
| 2/27/2025 15:58 | 71.6 | 12 | 72.32 | 11.1 | 72.5 | 13.1 |
| 2/27/2025 15:59 | 71.42 | 11.9 | 72.32 | 11 | 72.5 | 12.9 |
| 2/27/2025 16:00 | 71.42 | 11.8 | 72.32 | 10.9 | 72.32 | 12.9 |
| 2/27/2025 16:01 | 71.42 | 11.7 | 72.14 | 10.8 | 72.32 | 12.8 |
| 2/27/2025 16:02 | 71.42 | 11.6 | 72.14 | 10.7 | 72.14 | 12.8 |
| 2/27/2025 16:03 | 71.42 | 11.5 | 72.14 | 10.7 | 72.14 | 12.7 |
| 2/27/2025 16:04 | 71.42 | 11.4 | 72.14 | 10.7 | 72.14 | 12.6 |
| 2/27/2025 16:05 | 71.24 | 11.3 | 71.96 | 10.6 | 71.96 | 12.6 |
| 2/27/2025 16:06 | 71.24 | 11.3 | 71.96 | 10.5 | 71.96 | 12.5 |
| 2/27/2025 16:07 | 71.24 | 11.2 | 71.96 | 10.4 | 71.96 | 12.4 |
| 2/27/2025 16:08 | 71.24 | 11.1 | 71.96 | 10.3 | 71.96 | 12.4 |
| 2/27/2025 16:09 | 71.24 | 11 | 71.96 | 10.3 | 71.78 | 12.3 |
| 2/27/2025 16:10 | 71.24 | 11 | 71.78 | 10.2 | 71.78 | 12.2 |
| 2/27/2025 16:11 | 71.24 | 10.9 | 71.78 | 10.1 | 71.78 | 12.2 |
| 2/27/2025 16:12 | 71.24 | 10.8 | 71.78 | 10.1 | 71.78 | 12.1 |
| 2/27/2025 16:13 | 71.24 | 10.7 | 71.78 | 10 | 71.6 | 12 |
| 2/27/2025 16:14 | 71.24 | 10.7 | 71.78 | 9.9 | 71.6 | 12 |
| 2/27/2025 16:15 | 71.06 | 10.6 | 71.6 | 9.9 | 71.6 | 11.9 |
| 2/27/2025 16:16 | 71.24 | 10.5 | 71.6 | 9.9 | 71.6 | 11.9 |
| 2/27/2025 16:17 | 71.06 | 10.5 | 71.6 | 9.8 | 71.6 | 11.8 |
| 2/27/2025 16:18 | 71.06 | 10.4 | 71.6 | 9.7 | 71.42 | 11.7 |
| 2/27/2025 16:19 | 71.06 | 10.3 | 71.6 | 9.7 | 71.42 | 11.6 |
| 2/27/2025 16:20 | 71.06 | 10.3 | 71.6 | 9.7 | 71.42 | 11.6 |
| 2/27/2025 16:21 | 71.06 | 10.2 | 71.42 | 9.6 | 71.42 | 11.5 |
| 2/27/2025 16:22 | 71.06 | 10.2 | 71.42 | 9.5 | 71.42 | 11.4 |
| 2/27/2025 16:23 | 71.06 | 10.1 | 71.42 | 9.6 | 71.42 | 11.4 |
| 2/27/2025 16:24 | 71.06 | 10.1 | 71.42 | 9.4 | 71.24 | 11.4 |
| 2/27/2025 16:25 | 70.88 | 10 | 71.42 | 9.4 | 71.24 | 11.3 |
| 2/27/2025 16:26 | 70.88 | 10 | 71.24 | 9.3 | 71.24 | 11.2 |
| 2/27/2025 16:27 | 70.88 | 9.9 | 71.24 | 9.3 | 71.24 | 11.2 |
| 2/27/2025 16:28 | 70.88 | 9.8 | 71.24 | 9.3 | 71.24 | 11.1 |
| 2/27/2025 16:29 | 70.88 | 9.8 | 71.24 | 9.2 | 71.24 | 11.1 |
| 2/27/2025 16:30 | 70.88 | 9.7 | 71.24 | 9.2 | 71.06 | 11 |
| 2/27/2025 16:31 | 70.7 | 9.7 | 71.24 | 9.2 | 71.06 | 11 |
| 2/27/2025 16:32 | 70.7 | 9.7 | 71.06 | 9.1 | 71.06 | 10.9 |
| 2/27/2025 16:33 | 70.7 | 9.7 | 71.06 | 9.1 | 71.06 | 10.8 |
| 2/27/2025 16:34 | 70.7 | 9.6 | 71.06 | 9.1 | 71.06 | 10.8 |
| 2/27/2025 16:35 | 70.7 | 9.5 | 71.06 | 9 | 71.06 | 10.7 |
| 2/27/2025 16:36 | 70.7 | 9.5 | 71.06 | 9 | 70.88 | 10.7 |
| 2/27/2025 16:37 | 70.7 | 9.4 | 71.06 | 8.9 | 70.88 | 10.6 |
| 2/27/2025 16:38 | 70.7 | 9.4 | 71.06 | 8.9 | 70.88 | 10.6 |
| 2/27/2025 16:39 | 70.7 | 9.4 | 71.06 | 8.9 | 70.88 | 10.5 |
| 2/27/2025 16:40 | 70.7 | 9.4 | 71.06 | 8.8 | 70.88 | 10.5 |
| 2/27/2025 16:41 | 70.52 | 9.3 | 70.88 | 8.7 | 70.88 | 10.4 |
| 2/27/2025 16:42 | 70.7 | 9.2 | 70.88 | 8.7 | 70.88 | 10.4 |
| 2/27/2025 16:43 | 70.7 | 9.3 | 70.88 | 8.7 | 70.88 | 10.3 |
| 2/27/2025 16:44 | 70.52 | 9.2 | 70.88 | 8.7 | 70.88 | 10.3 |
| 2/27/2025 16:45 | 70.52 | 9.1 | 70.88 | 8.7 | 70.7 | 10.3 |
| 2/27/2025 16:46 | 70.52 | 9.1 | 70.88 | 8.6 | 70.7 | 10.2 |
| 2/27/2025 16:47 | 70.52 | 9 | 70.7 | 8.6 | 70.7 | 10.2 |
| 2/27/2025 16:48 | 70.52 | 9 | 70.88 | 8.6 | 70.7 | 10.1 |
| 2/27/2025 16:49 | 70.52 | 9 | 70.88 | 8.5 | 70.7 | 10 |
| 2/27/2025 16:50 | 70.52 | 8.9 | 70.7 | 8.5 | 70.7 | 10 |
| 2/27/2025 16:51 | 70.52 | 8.9 | 70.7 | 8.5 | 70.7 | 10 |
| 2/27/2025 16:52 | 70.34 | 8.9 | 70.7 | 8.4 | 70.7 | 9.9 |
| 2/27/2025 16:53 | 70.34 | 8.9 | 70.7 | 8.4 | 70.7 | 9.9 |
| 2/27/2025 16:54 | 70.34 | 8.8 | 70.7 | 8.4 | 70.7 | 9.8 |
| 2/27/2025 16:55 | 70.34 | 8.8 | 70.7 | 8.4 | 70.52 | 9.8 |
| 2/27/2025 16:56 | 70.34 | 8.8 | 70.7 | 8.3 | 70.52 | 9.7 |
| 2/27/2025 16:57 | 70.34 | 8.7 | 70.52 | 8.3 | 70.52 | 9.7 |
| 2/27/2025 16:58 | 70.34 | 8.7 | 70.52 | 8.2 | 70.52 | 9.7 |
| 2/27/2025 16:59 | 70.34 | 8.7 | 70.52 | 8.2 | 70.52 | 9.7 |
| 2/27/2025 17:00 | 70.34 | 8.6 | 70.52 | 8.2 | 70.52 | 9.6 |
| 2/27/2025 17:01 | 70.16 | 8.6 | 70.52 | 8.2 | 70.52 | 9.6 |
| 2/27/2025 17:02 | 70.34 | 8.6 | 70.52 | 8.2 | 70.52 | 9.6 |
| 2/27/2025 17:03 | 70.16 | 8.6 | 70.52 | 8.1 | 70.52 | 9.5 |
| 2/27/2025 17:04 | 70.16 | 8.5 | 70.52 | 8.1 | 70.52 | 9.5 |
| 2/27/2025 17:05 | 70.16 | 8.5 | 70.34 | 8.1 | 70.52 | 9.4 |
| 2/27/2025 17:06 | 70.16 | 8.5 | 70.34 | 8.1 | 70.34 | 9.4 |
| 2/27/2025 17:07 | 70.16 | 8.4 | 70.34 | 8 | 70.34 | 9.3 |
| 2/27/2025 17:08 | 70.16 | 8.4 | 70.34 | 8 | 70.34 | 9.3 |
| 2/27/2025 17:09 | 70.16 | 8.4 | 70.34 | 8 | 70.34 | 9.3 |
| 2/27/2025 17:10 | 70.16 | 8.4 | 70.34 | 8 | 70.34 | 9.3 |
| 2/27/2025 17:11 | 70.16 | 8.4 | 70.34 | 8 | 70.34 | 9.2 |
| 2/27/2025 17:12 | 69.98 | 8.3 | 70.34 | 7.9 | 70.34 | 9.3 |
| 2/27/2025 17:13 | 70.16 | 8.3 | 70.34 | 7.9 | 70.34 | 9.2 |
| 2/27/2025 17:14 | 69.98 | 8.3 | 70.34 | 7.9 | 70.34 | 9.1 |
| 2/27/2025 17:15 | 69.98 | 8.3 | 70.34 | 7.8 | 70.34 | 9.1 |
| 2/27/2025 17:16 | 69.98 | 8.2 | 70.16 | 7.8 | 70.16 | 9.1 |
| 2/27/2025 17:17 | 69.98 | 8.2 | 70.34 | 7.8 | 70.16 | 9 |
| 2/27/2025 17:18 | 69.98 | 8.2 | 70.16 | 7.8 | 70.34 | 9 |
| 2/27/2025 17:19 | 69.98 | 8.2 | 70.16 | 7.8 | 70.16 | 9 |
| 2/27/2025 17:20 | 69.98 | 8.2 | 70.16 | 7.7 | 70.16 | 9 |
| 2/27/2025 17:21 | 69.98 | 8.1 | 70.16 | 7.7 | 70.16 | 8.9 |
| 2/27/2025 17:22 | 69.98 | 8.1 | 70.16 | 7.7 | 70.16 | 8.9 |
| 2/27/2025 17:23 | 69.98 | 8.1 | 70.16 | 7.7 | 70.16 | 8.8 |
| 2/27/2025 17:24 | 69.98 | 8.1 | 70.16 | 7.7 | 70.16 | 8.8 |
| 2/27/2025 17:25 | 69.98 | 8 | 70.16 | 7.7 | 70.16 | 8.8 |
| 2/27/2025 17:26 | 69.98 | 8.1 | 70.16 | 7.6 | 70.16 | 8.8 |
| 2/27/2025 17:27 | 69.98 | 8 | 70.16 | 7.6 | 70.16 | 8.7 |
| 2/27/2025 17:28 | 69.98 | 8 | 69.98 | 7.6 | 70.16 | 8.7 |
| 2/27/2025 17:29 | 69.98 | 8 | 70.16 | 7.6 | 70.16 | 8.7 |
| 2/27/2025 17:30 | 69.8 | 7.9 | 70.16 | 7.5 | 70.16 | 8.6 |
| 2/27/2025 17:31 | 69.8 | 7.9 | 69.98 | 7.5 | 70.16 | 8.6 |
| 2/27/2025 17:32 | 69.8 | 7.9 | 69.98 | 7.6 | 70.16 | 8.6 |
| 2/27/2025 17:33 | 69.8 | 7.9 | 69.98 | 7.5 | 70.16 | 8.6 |
| 2/27/2025 17:34 | 69.8 | 7.8 | 69.98 | 7.4 | 69.98 | 8.6 |
| 2/27/2025 17:35 | 69.8 | 7.8 | 69.98 | 7.5 | 69.98 | 8.5 |
| 2/27/2025 17:36 | 69.8 | 7.8 | 69.98 | 7.4 | 69.98 | 8.5 |
| 2/27/2025 17:37 | 69.8 | 7.8 | 69.98 | 7.4 | 69.98 | 8.5 |
| 2/27/2025 17:38 | 69.8 | 7.8 | 69.98 | 7.4 | 69.98 | 8.5 |
| 2/27/2025 17:39 | 69.8 | 7.8 | 69.98 | 7.4 | 69.98 | 8.4 |
| 2/27/2025 17:40 | 69.8 | 7.8 | 69.98 | 7.4 | 69.98 | 8.4 |
| 2/27/2025 17:41 | 69.8 | 7.7 | 69.98 | 7.4 | 69.98 | 8.4 |
| 2/27/2025 17:42 | 69.62 | 7.7 | 69.98 | 7.3 | 69.98 | 8.4 |
| 2/27/2025 17:43 | 69.8 | 7.7 | 69.98 | 7.3 | 69.98 | 8.4 |
| 2/27/2025 17:44 | 69.8 | 7.7 | 69.98 | 7.3 | 69.98 | 8.3 |
| 2/27/2025 17:45 | 69.62 | 7.6 | 69.98 | 7.3 | 69.98 | 8.3 |
| 2/27/2025 17:46 | 69.62 | 7.7 | 69.98 | 7.4 | 69.98 | 8.3 |
| 2/27/2025 17:47 | 69.62 | 7.6 | 69.8 | 7.3 | 69.98 | 8.3 |
| 2/27/2025 17:48 | 69.62 | 7.6 | 69.8 | 7.3 | 69.98 | 8.2 |
| 2/27/2025 17:49 | 69.62 | 7.6 | 69.8 | 7.3 | 69.98 | 8.2 |
| 2/27/2025 18:01 | 69.44 | 7.4 | 69.8 | 7.2 | 69.98 | 8.2 |
| 2/27/2025 18:02 | 69.44 | 7.4 | 69.8 | 7.1 | 69.98 | 8.2 |
| 2/27/2025 18:03 | 69.44 | 7.4 | 69.8 | 7.2 | 69.8 | 8.2 |
| 2/27/2025 18:04 | 69.44 | 7.4 | 69.8 | 7.2 | 69.8 | 8.2 |
| 2/27/2025 18:05 | 69.44 | 7.4 | 69.8 | 7.2 | 69.8 | 8.1 |
| 2/27/2025 18:06 | 69.44 | 7.4 | 69.8 | 7.1 | 69.8 | 8.1 |
| 2/27/2025 18:07 | 69.44 | 7.3 | 69.8 | 7.1 | 69.8 | 8.1 |
| 2/27/2025 18:08 | 69.44 | 7.3 | 69.8 | 7.1 | 69.8 | 8.1 |
| 2/27/2025 18:09 | 69.44 | 7.3 | 69.8 | 7.1 | 69.8 | 8 |
| 2/27/2025 18:10 | 69.44 | 7.3 | 69.8 | 7.1 | 69.8 | 8 |
| 2/27/2025 18:11 | 69.44 | 7.3 | 69.8 | 7.1 | 69.8 | 7.9 |
| 2/27/2025 18:12 | 69.44 | 7.3 | 69.62 | 7 | 69.8 | 8 |
| 2/27/2025 18:13 | 69.44 | 7.2 | 69.8 | 7 | 69.8 | 7.9 |
| 2/27/2025 18:14 | 69.44 | 7.2 | 69.8 | 7.1 | 69.8 | 7.9 |
| 2/27/2025 18:15 | 69.44 | 7.2 | 69.62 | 7 | 69.8 | 7.9 |
| 2/27/2025 18:16 | 69.44 | 7.2 | 69.62 | 7 | 69.8 | 7.9 |
| 2/27/2025 18:17 | 69.44 | 7.2 | 69.8 | 7 | 69.8 | 7.9 |
| 2/27/2025 18:18 | 69.44 | 7.2 | 69.62 | 7 | 69.8 | 7.9 |
| 2/27/2025 18:19 | 69.44 | 7.2 | 69.62 | 7 | 69.8 | 7.8 |
| 2/27/2025 18:20 | 69.44 | 7.2 | 69.62 | 6.9 | 69.8 | 7.8 |
| 2/27/2025 18:21 | 69.44 | 7.2 | 69.62 | 6.9 | 69.8 | 7.8 |
| 2/27/2025 18:22 | 69.26 | 7.1 | 69.62 | 6.9 | 69.8 | 7.8 |
| 2/27/2025 18:23 | 69.26 | 7.1 | 69.62 | 6.9 | 69.8 | 7.8 |
| 2/27/2025 18:24 | 69.26 | 7.1 | 69.62 | 6.9 | 69.8 | 7.8 |
| 2/27/2025 18:25 | 69.26 | 7.1 | 69.62 | 6.9 | 69.8 | 7.7 |
| 2/27/2025 18:26 | 69.26 | 7 | 69.62 | 6.9 | 69.62 | 7.7 |
| 2/27/2025 18:27 | 69.26 | 7.1 | 69.62 | 6.8 | 69.8 | 7.7 |
| 2/27/2025 18:28 | 69.26 | 7 | 69.62 | 6.9 | 69.8 | 7.7 |
| 2/27/2025 18:29 | 69.26 | 7 | 69.62 | 6.8 | 69.62 | 7.6 |
| 2/27/2025 18:30 | 69.26 | 7 | 69.62 | 6.8 | 69.62 | 7.6 |
| 2/27/2025 18:31 | 69.26 | 7.1 | 69.62 | 6.9 | 69.62 | 7.6 |
| 2/27/2025 18:32 | 69.26 | 7 | 69.62 | 6.8 | 69.62 | 7.6 |
| 2/27/2025 18:33 | 69.26 | 7 | 69.62 | 6.8 | 69.62 | 7.6 |
| 2/27/2025 18:34 | 69.26 | 7 | 69.62 | 6.8 | 69.62 | 7.5 |
| 2/27/2025 18:35 | 69.26 | 7 | 69.62 | 6.8 | 69.62 | 7.6 |
| 2/27/2025 18:36 | 69.08 | 7 | 69.62 | 6.8 | 69.62 | 7.5 |
| 2/27/2025 18:37 | 69.26 | 6.9 | 69.44 | 6.7 | 69.62 | 7.5 |
| 2/27/2025 18:38 | 69.26 | 7 | 69.44 | 6.7 | 69.62 | 7.5 |
| 2/27/2025 18:39 | 69.26 | 7 | 69.62 | 6.7 | 69.62 | 7.5 |
| 2/27/2025 18:40 | 69.26 | 6.9 | 69.44 | 6.8 | 69.62 | 7.4 |
| 2/27/2025 18:41 | 69.26 | 7 | 69.44 | 6.7 | 69.62 | 7.4 |
| 2/27/2025 18:42 | 69.26 | 6.9 | 69.44 | 6.7 | 69.62 | 7.5 |
| 2/27/2025 18:43 | 69.26 | 6.9 | 69.44 | 6.7 | 69.62 | 7.4 |
| 2/27/2025 18:44 | 69.26 | 6.9 | 69.44 | 6.7 | 69.62 | 7.4 |
| 2/27/2025 18:45 | 69.26 | 6.9 | 69.44 | 6.7 | 69.62 | 7.4 |
| 2/27/2025 18:46 | 69.08 | 6.8 | 69.44 | 6.7 | 69.62 | 7.4 |
| 2/27/2025 18:47 | 69.08 | 6.9 | 69.44 | 6.6 | 69.62 | 7.4 |
| 2/27/2025 18:48 | 69.26 | 6.9 | 69.44 | 6.7 | 69.62 | 7.3 |
| 2/27/2025 18:49 | 69.26 | 6.9 | 69.44 | 6.6 | 69.62 | 7.3 |
| 2/27/2025 18:50 | 69.08 | 6.9 | 69.44 | 6.6 | 69.44 | 7.4 |
| 2/27/2025 18:51 | 69.08 | 6.8 | 69.44 | 6.6 | 69.44 | 7.3 |
| 2/27/2025 18:52 | 69.08 | 6.8 | 69.44 | 6.6 | 69.44 | 7.3 |
| 2/27/2025 18:53 | 69.08 | 6.8 | 69.44 | 6.6 | 69.44 | 7.3 |
| 2/27/2025 18:54 | 69.08 | 6.8 | 69.44 | 6.6 | 69.44 | 7.3 |
| 2/27/2025 18:55 | 69.08 | 6.8 | 69.44 | 6.6 | 69.44 | 7.3 |
| 2/27/2025 18:56 | 69.08 | 6.8 | 69.44 | 6.6 | 69.44 | 7.3 |
| 2/27/2025 18:57 | 69.08 | 6.8 | 69.44 | 6.6 | 69.44 | 7.2 |
| 2/27/2025 18:58 | 69.08 | 6.8 | 69.26 | 6.6 | 69.44 | 7.2 |
| 2/27/2025 18:59 | 69.08 | 6.8 | 69.44 | 6.5 | 69.44 | 7.2 |
| 2/27/2025 19:00 | 69.08 | 6.8 | 69.26 | 6.5 | 69.44 | 7.2 |
| 2/27/2025 19:01 | 69.08 | 6.8 | 69.44 | 6.5 | 69.44 | 7.2 |
| 2/27/2025 19:02 | 69.08 | 6.7 | 69.26 | 6.6 | 69.44 | 7.2 |
| 2/27/2025 19:03 | 69.08 | 6.8 | 69.26 | 6.5 | 69.44 | 7.2 |
| 2/27/2025 19:04 | 69.08 | 6.7 | 69.26 | 6.5 | 69.44 | 7.2 |
| 2/27/2025 19:05 | 69.08 | 6.7 | 69.26 | 6.5 | 69.44 | 7.2 |
| 2/27/2025 19:06 | 69.08 | 6.7 | 69.26 | 6.5 | 69.44 | 7.1 |
| 2/27/2025 19:07 | 69.08 | 6.7 | 69.26 | 6.5 | 69.44 | 7.1 |
| 2/27/2025 19:08 | 69.08 | 6.7 | 69.26 | 6.5 | 69.44 | 7.1 |
| 2/27/2025 19:09 | 69.08 | 6.7 | 69.26 | 6.5 | 69.44 | 7.1 |
| 2/27/2025 19:10 | 69.08 | 6.6 | 69.26 | 6.5 | 69.44 | 7.1 |
| 2/27/2025 19:11 | 69.08 | 6.7 | 69.26 | 6.5 | 69.44 | 7.1 |
| 2/27/2025 19:12 | 69.08 | 6.7 | 69.26 | 6.4 | 69.26 | 7.1 |
| 2/27/2025 19:13 | 69.08 | 6.7 | 69.26 | 6.4 | 69.44 | 7 |
| 2/27/2025 19:14 | 69.08 | 6.6 | 69.26 | 6.4 | 69.26 | 7 |
| 2/27/2025 19:15 | 69.08 | 6.6 | 69.26 | 6.4 | 69.44 | 7 |
| 2/27/2025 19:16 | 69.08 | 6.6 | 69.26 | 6.4 | 69.44 | 7 |
| 2/27/2025 19:17 | 69.08 | 6.6 | 69.26 | 6.4 | 69.26 | 7 |
| 2/27/2025 19:18 | 69.08 | 6.6 | 69.26 | 6.4 | 69.44 | 6.9 |
| 2/27/2025 19:19 | 69.08 | 6.6 | 69.26 | 6.4 | 69.26 | 7 |
| 2/27/2025 19:20 | 69.08 | 6.6 | 69.26 | 6.4 | 69.26 | 7 |
| 2/27/2025 19:21 | 69.08 | 6.6 | 69.08 | 6.3 | 69.26 | 7 |
| 2/27/2025 19:22 | 68.9 | 6.6 | 69.26 | 6.4 | 69.26 | 7 |
| 2/27/2025 19:23 | 69.08 | 6.6 | 69.26 | 6.3 | 69.26 | 6.9 |
| 2/27/2025 19:24 | 68.9 | 6.5 | 69.26 | 6.3 | 69.26 | 6.9 |
| 2/27/2025 19:25 | 69.08 | 6.6 | 69.26 | 6.3 | 69.26 | 6.9 |
| 2/27/2025 19:26 | 69.08 | 6.5 | 69.26 | 6.3 | 69.26 | 6.9 |
| 2/27/2025 19:27 | 68.9 | 6.5 | 69.08 | 6.3 | 69.26 | 6.9 |
| 2/27/2025 19:28 | 68.9 | 6.5 | 69.26 | 6.3 | 69.26 | 6.9 |
| 2/27/2025 19:29 | 68.9 | 6.5 | 69.26 | 6.3 | 69.26 | 6.9 |
| 2/27/2025 19:30 | 68.9 | 6.5 | 69.26 | 6.3 | 69.26 | 6.8 |
| 2/27/2025 19:31 | 68.9 | 6.5 | 69.08 | 6.3 | 69.26 | 6.9 |
| 2/27/2025 19:32 | 69.08 | 6.5 | 69.08 | 6.3 | 69.26 | 6.9 |
| 2/27/2025 19:33 | 68.9 | 6.5 | 69.08 | 6.3 | 69.26 | 6.9 |
| 2/27/2025 19:34 | 69.08 | 6.5 | 69.08 | 6.3 | 69.26 | 6.8 |
| 2/27/2025 19:35 | 68.9 | 6.5 | 69.08 | 6.2 | 69.26 | 6.8 |
| 2/27/2025 19:36 | 68.9 | 6.5 | 69.08 | 6.3 | 69.26 | 6.8 |
| 2/27/2025 19:37 | 68.9 | 6.4 | 69.08 | 6.3 | 69.26 | 6.8 |
| 2/27/2025 19:38 | 68.9 | 6.4 | 69.08 | 6.3 | 69.26 | 6.8 |
| 2/27/2025 19:39 | 68.9 | 6.5 | 69.08 | 6.2 | 69.26 | 6.8 |
| 2/27/2025 19:40 | 68.9 | 6.4 | 69.26 | 6.3 | 69.26 | 6.7 |
| 2/27/2025 19:41 | 68.9 | 6.5 | 69.08 | 6.3 | 69.26 | 6.8 |
| 2/27/2025 19:42 | 68.9 | 6.4 | 69.08 | 6.2 | 69.26 | 6.8 |
| 2/27/2025 19:43 | 68.9 | 6.4 | 69.08 | 6.2 | 69.26 | 6.7 |
| 2/27/2025 19:44 | 68.9 | 6.4 | 69.08 | 6.2 | 69.26 | 6.7 |
| 2/27/2025 19:45 | 68.9 | 6.4 | 69.08 | 6.2 | 69.26 | 6.8 |
| 2/27/2025 19:46 | 68.9 | 6.4 | 69.08 | 6.2 | 69.26 | 6.7 |
| 2/27/2025 19:47 | 68.9 | 6.4 | 69.08 | 6.2 | 69.26 | 6.7 |
| 2/27/2025 19:48 | 68.9 | 6.4 | 69.08 | 6.1 | 69.26 | 6.7 |
| 2/27/2025 19:49 | 68.9 | 6.4 | 69.08 | 6.2 | 69.26 | 6.7 |
| 2/27/2025 19:50 | 68.9 | 6.3 | 69.08 | 6.2 | 69.26 | 6.7 |
| 2/27/2025 19:51 | 68.9 | 6.4 | 69.08 | 6.1 | 69.26 | 6.7 |
| 2/27/2025 19:52 | 68.9 | 6.4 | 69.08 | 6.1 | 69.26 | 6.7 |
| 2/27/2025 19:53 | 68.9 | 6.4 | 69.08 | 6.2 | 69.26 | 6.6 |
| 2/27/2025 19:54 | 68.9 | 6.3 | 69.08 | 6.1 | 69.26 | 6.6 |
| 2/27/2025 19:55 | 68.9 | 6.3 | 69.08 | 6.1 | 69.26 | 6.7 |
| 2/27/2025 19:56 | 68.9 | 6.3 | 69.08 | 6.1 | 69.26 | 6.6 |
| 2/27/2025 19:57 | 68.9 | 6.3 | 69.08 | 6.2 | 69.26 | 6.6 |
| 2/27/2025 19:58 | 68.9 | 6.3 | 69.08 | 6.1 | 69.26 | 6.6 |
| 2/27/2025 19:59 | 68.9 | 6.3 | 69.08 | 6.1 | 69.26 | 6.6 |
| 2/27/2025 20:00 | 68.9 | 6.3 | 69.08 | 6.1 | 69.26 | 6.6 |
| 2/27/2025 20:01 | 68.9 | 6.3 | 69.08 | 6.1 | 69.26 | 6.6 |
| 2/27/2025 20:02 | 68.9 | 6.3 | 69.08 | 6.1 | 69.26 | 6.6 |
| 2/27/2025 20:03 | 68.9 | 6.3 | 69.08 | 6.1 | 69.26 | 6.6 |
| 2/27/2025 20:04 | 68.72 | 6.3 | 69.08 | 6.1 | 69.26 | 6.5 |
| 2/27/2025 20:05 | 68.9 | 6.3 | 69.08 | 6.1 | 69.26 | 6.5 |
| 2/27/2025 20:06 | 68.9 | 6.3 | 69.08 | 6 | 69.26 | 6.5 |
| 2/27/2025 20:07 | 68.9 | 6.3 | 69.08 | 6 | 69.26 | 6.5 |
| 2/27/2025 20:08 | 68.9 | 6.3 | 69.08 | 6 | 69.26 | 6.5 |
| 2/27/2025 20:09 | 68.9 | 6.3 | 69.08 | 6 | 69.26 | 6.5 |
| 2/27/2025 20:10 | 68.9 | 6.2 | 69.08 | 6 | 69.08 | 6.5 |
| 2/27/2025 20:11 | 68.72 | 6.2 | 69.08 | 6 | 69.26 | 6.5 |

S. Table 2. NOA raw data

|  | Measured NO level (PPM) | | | | |
| --- | --- | --- | --- | --- | --- |
| Time | Sample 1 | Sample 2 | Sample 3 | Mean | S.D. |
| 5 min | 77.5 | 89.0 | 121.5 | 96.0 | 22.8 |
| 30 min | 85.0 | 87.0 | 93.3 | 88.4 | 4.3 |
| 1 h | 61.5 | 67.8 | 66.3 | 65.2 | 3.3 |
| 2 h | 59.5 | 74.5 | 68.5 | 67.5 | 7.5 |
| 3 h | 62.3 | 77.3 | 108.5 | 82.7 | 23.6 |
| 4 h | 109.0 | 101.3 | 156.8 | 122.3 | 30.1 |
| 5 h | 128.8 | 142.5 | 119.8 | 130.3 | 11.5 |
| 6 h | 71.5 | 126.3 | 108.8 | 102.2 | 28.0 |
| 7 h | 94.8 | 114.3 | 100.5 | 103.2 | 10.0 |
| 8 h | 79.8 | 107.8 | 98.3 | 95.3 | 14.2 |
| 9 h | 120.8 | 121.0 | 186.5 | 142.8 | 37.9 |
| 10 h | 160.5 | 94.0 | 140.8 | 131.8 | 34.2 |
| 12 h | 102.5 | 120.5 | 115.3 | 112.8 | 9.3 |

Bacterial count data

S Table 3. CFU count of the recovered *B. atrophaeus* after NO sterilization at RT

| Time | Dilution ratio | Unit # | Sample 1 | Sample 2 | Sample 3 | Average |
| --- | --- | --- | --- | --- | --- | --- |
| 0 h | 10000 | 1 | 58 | 33 | 55 | 53 |
|  |  | 2 | 89 | 112 | 55 |  |
|  |  | 3 | 15 | 36 | 28 |  |
| 2 h | 1000 | 1 | 71 | 84 | 93 | 82 |
|  | 10000 | 1 | 33 | 42 | 36 | 38 |
|  |  | 2 | 34 | 35 | 48 |  |
| 4 h | 10 | 1 | 12 | 13 | 20 | 83 |
|  |  | 2 | 112 | 14 | 123 |  |
|  |  | 3 | 110 | 195 | 152 |  |
| 6 h | N.D. | 1 | 124 | 62 | 117 | 34 |
|  |  | 2 | 0 | 0 | 0 |  |
|  |  | 3 | 0 | 5 | 0 |  |
| 8 h | N.D. | 1 | 0 | 0 | 0 | 0 |
|  |  | 2 | 0 | 0 | 0 |  |
|  |  | 3 | 0 | 0 | 0 |  |

Each sterilization unit contains three BI samples. Different sterilization unit numbers indicate different independent repeats of the study.

S Table 4. CFU count of the recovered *B. subtilis* after NO sterilization at RT

| Time | Dilution ratio | Unit # | Sample 1 | Sample 2 | Sample 3 | Average |
| --- | --- | --- | --- | --- | --- | --- |
| 0 h | 10000 | 1 | 371 | 346 | 279 | 299 |
|  |  | 2 | 292 | 252 | 289 |  |
|  |  | 3 | 250 | 338 | 270 |  |
| 2 h | 10000 | 1 | 178 | 94 | 87 | 151 |
|  |  | 2 | 102 | 110 | 98 |  |
|  |  | 3 | 204 | 237 | 248 |  |
| 4 h | 1000 | 1 | 166 | 192 | 118 | 277 |
|  |  | 2 | 368 | 258 | 201 |  |
|  |  | 3 | 294 | 453 | 447 |  |
| 6 h | 200 | 1 | 570 | 398 | 540 | 292 |
|  |  | 2 | 128 | 243 | 343 |  |
|  |  | 3 | 163 | 145 | 104 |  |
| 8 h | 20 | 1 | 0 | 0 | 0 | 22 |
|  |  | 2 | 0 | 0 | 0 |  |
|  |  | 3 | 76 | 89 | 33 |  |
| 10 h | N.D. | 1 | 0 | 0 | 0 | 0 |

Each sterilization unit contains three BI samples. Different sterilization unit numbers indicate different independent repeats of the study.

S Table 5. CFU count of the recovered *G. stearothermophilus* after NO sterilization at RT

| Time | Dilution ratio | Unit # | Sample 1 | Sample 2 | Sample 3 | Average |
| --- | --- | --- | --- | --- | --- | --- |
| 0 h | 10000 | 1 | 113 | 89 | 108 | 128 |
|  |  | 2 | 114 | 91 | 129 |  |
|  |  | 3 | 94 | 314 | 98 |  |
| 2 h | 10000 | 1 | 74 | 121 | 48 | 130 |
|  |  | 2 | 280 | 178 | 177 |  |
|  |  | 3 | 94 | 122 | 73 |  |
| 4 h | 10000 | 1 | 57 | 36 | 124 | 72 |
|  | 1000 | 2 | 198 | 189 | 252 | 208 |
|  |  | 3 | 194 | 145 | 273 |  |
| 6 h | 10 | 1 | 56 | 82 | 10 | 57 |
|  |  | 2 | 43 | 152 | 44 |  |
|  |  | 3 | 78 | 14 | 32 |  |
| 8 h | N.D. | 1 | 0 | 26 | 0 | 9 |
| 10 h | N.D. | 1 | 0 | 0 | 0 | 0 |

Each sterilization unit contains three BI samples. Different sterilization unit numbers indicate different independent repeats of the study.

S. Table 6. CFU count of the recovered *E. coli* after NO sterilization at RT

| Time | Dilution ratio | Unit # | Sample 1 | Sample 2 | Sample 3 | Average |
| --- | --- | --- | --- | --- | --- | --- |
| 0 h | 10000 | 1 | 127 | 191 | 90 | 255 |
|  |  | 2 | 314 | 463 | 345 |  |
| 0.5 h | 10000 | 1 | 224 | 212 | 219 | 204 |
|  |  | 2 | 259 | 222 | 246 |  |
|  |  | 3 | 159 | 150 | 149 |  |
| 1 h | 10000 | 1 | 62 | 81 | 52 | 93 |
|  |  | 2 | 152 | 148 | 104 |  |
|  |  | 3 | 63 | 95 | 81 |  |
| 1.5 h | 1000 | 1 | 78 | 62 | 160 | 65 |
|  |  | 2 | 40 | 37 | 47 |  |
|  |  | 3 | 65 | 60 | 33 |  |
| 2 h | 100 | 1 | 103 | 22 | 39 | 26 |
|  |  | 2 | 0 | 0 | 0 |  |
| 2.5 h | N.D. | 1 | 0 | 0 | 0 | 0 |
|  |  | 2 | 0 | 0 | 0 |  |

Each sterilization unit contains one bacterial suspension aliquot. Different sterilization unit numbers indicate different independent repeats of the study.

S. Table 7. CFU count of the recovered *S. aureus* after NO sterilization at RT

| Time | Dilution ratio | Unit # | Sample 1 | Sample 2 | Sample 3 | Average |
| --- | --- | --- | --- | --- | --- | --- |
| 0 h | 10000 | 1 | 8 | 10 | 21 | 199 |
|  |  | 2 | 314 | 355 | 259 |  |
|  |  | 3 | 252 | 304 | 271 |  |
| 1 h | 10000 | 1 | 169 | 150 | 167 | 99 |
|  |  | 2 | 93 | 55 | 62 |  |
|  |  | 3 | 53 | 80 | 59 |  |
|  | 1000 | 1 | 129 | 128 | 161 | 143 |
|  |  | 2 | 106 | 195 | 185 |  |
|  |  | 3 | 136 | 133 | 113 |  |
| 2 h | 100 | 1 | 266 | 182 | 170 | 65 |
|  |  | 2 | 140 | 138 | 103 |  |
|  |  | 3 | 318 | 204 | 212 |  |
|  | 1000 | 1 | 1 | 0 | 0 | 0 |
|  |  | 2 | 0 | 0 | 0 |  |
|  |  | 3 | 0 | 0 | 1 |  |
| 3 h | N.D. | 1 | 5 | 0 | 85 | 61 |
|  |  | 2 | 0 | 0 | 277 |  |
| 4 h | N.D. | 1 | 0 | 0 | 0 | 0 |

Each sterilization unit contains one bacterial suspension aliquot. Different sterilization unit numbers indicate different independent repeats of the study.
