# Supplementary material for: Using nitric oxide (NO) for gas sterilization at room temperature: A novel sterilization modality
Source: PLoS One. 2026 Aug 21;21(8):e0355735. doi: 10.1371/journal.pone.0355735 (PMC13496434; doi:10.1371/journal.pone.0355735)
Supplement: Peer review history

# Peer review: Decision Letter 0

**DOI:** 10.1371/journal.pone.0355735.r001

**Authors:** Paulo Laranjeira

9 Jun 2026

Dear Dr. He,

Thank you for submitting your manuscript to PLOS ONE. After careful consideration, we feel that it has merit but does not fully meet PLOS ONE’s publication criteria as it currently stands. Therefore, we invite you to submit a revised version of the manuscript that addresses the points raised during the review process.

The following key issues must be addressed to ensure that the proposed sterilization approach is scientifically robust, reproducible, and sufficiently described for proper evaluation:

plosone@plos.org. When you're ready to submit your revision, log on to https://www.editorialmanager.com/pone/ and select the 'Submissions Needing Revision' folder to locate your manuscript file.

- A letter that responds to each point raised by the academic editor and reviewer(s). You should upload this letter as a separate file labeled 'Response to Reviewers'.
- A marked-up copy of your manuscript that highlights changes made to the original version. You should upload this as a separate file labeled 'Revised Manuscript with Track Changes'.
- An unmarked version of your revised paper without tracked changes. You should upload this as a separate file labeled 'Manuscript'.

If you would like to make changes to your financial disclosure, please include your updated statement in your cover letter. Guidelines for resubmitting your figure files are available below the reviewer comments at the end of this letter.

If applicable, we recommend that you deposit your laboratory protocols in protocols.io to enhance the reproducibility of your results. Protocols.io assigns your protocol its own identifier (DOI) so that it can be cited independently in the future. For instructions see: https://journals.plos.org/plosone/s/submission-guidelines#loc-laboratory-protocols. Additionally, PLOS ONE offers an option for publishing peer-reviewed Lab Protocol articles, which describe protocols hosted on protocols.io. Read more information on sharing protocols at https://plos.org/protocols?utm_medium=editorial-email&utm_source=authorletters&utm_campaign=protocols.

As the corresponding author, your ORCID iD is verified in the submission system and will appear in the published article. PLOS supports the use of ORCID, and we encourage all coauthors to register for an ORCID iD and use it as well. Please encourage your coauthors to verify their ORCID iD within the submission system before final acceptance, as unverified ORCID iDs will not appear in the published article. *Only*  the individual author can complete the verification step; PLOS staff *cannot*  verify ORCID iDs on behalf of authors.

We look forward to receiving your revised manuscript.

Kind regards,

Paulo Laranjeira

Academic Editor

PLOS One

Journal requirements:

When submitting your revision, we need you to address these additional requirements.

1. Please ensure that your manuscript meets PLOS ONE's style requirements, including those for file naming. The PLOS ONE style templates can be found at

https://journals.plos.org/plosone/s/file?id=wjVg/PLOSOne_formatting_sample_main_body.pdf and

https://journals.plos.org/plosone/s/file?id=ba62/PLOSOne_formatting_sample_title_authors_affiliations.pdf

2. Thank you for stating the following financial disclosure:

“This study was funded and conducted by Sterile State Inc.  The funder provided support in the form of salaries for authors. Company website: https://sterilestate.com/.”

Please state what role the funders took in the study.  If the funders had no role, please state: "The funders had no role in study design, data collection and analysis, decision to publish, or preparation of the manuscript."

If this statement is not correct you must amend it as needed.

Please include this amended Role of Funder statement in your cover letter; we will change the online submission form on your behalf.

3. Thank you for stating the following in the Competing Interests section:

“The funder provided support in the form of salaries for authors, but did not have any roles in the experiment design, data collection and analysis.  The funder was involved in the decision to submit the work for publication.”

Please confirm that this does not alter your adherence to all PLOS ONE policies on sharing data and materials, by including the following statement: "This does not alter our adherence to  PLOS ONE policies on sharing data and materials.” (as detailed online in our guide for authors http://journals.plos.org/plosone/s/competing-interests).  If there are restrictions on sharing of data and/or materials, please state these. Please note that we cannot proceed with consideration of your article until this information has been declared.

Please include your updated Competing Interests statement in your cover letter; we will change the online submission form on your behalf.

4. In the online submission form you indicate that your data is not available for proprietary reasons and have provided a contact point for accessing this data. Please note that your current contact point is a co-author on this manuscript. According to our Data Policy, the contact point must not be an author on the manuscript and must be an institutional contact, ideally not an individual. Please revise your data statement to a non-author institutional point of contact, such as a data access or ethics committee, and send this to us via return email. Please also include contact information for the third party organization, and please include the full citation of where the data can be found.

5. Please update your submission to use the PLOS LaTeX template. The template and more information on our requirements for LaTeX submissions can be found at http://journals.plos.org/plosone/s/latex.

If the reviewer comments include a recommendation to cite specific previously published works, please review and evaluate these publications to determine whether they are relevant and should be cited. There is no requirement to cite these works unless the editor has indicated otherwise.

[Note: HTML markup is below. Please do not edit.]

Reviewers' comments:

Reviewer's Responses to Questions

**Comments to the Author**

1. Is the manuscript technically sound, and do the data support the conclusions?

Reviewer #1: Partly

Reviewer #2: Partly

**********

2. Has the statistical analysis been performed appropriately and rigorously?

Reviewer #1: No

Reviewer #2: No

**********

3. Have the authors made all data underlying the findings in their manuscript fully available?

The PLOS Data policy

Reviewer #1: No

Reviewer #2: Yes

**********

4. Is the manuscript presented in an intelligible fashion and written in standard English?

Reviewer #1: Yes

Reviewer #2: No

**********

Reviewer #1: Lines 112-113: Spore count protocol should be detailed for spore count to be successful and reproducible. Otherwise, there could be wrong counting. Was the manufacturer recommendation followed? Two different manufacturers were used.

Line 125: no details for spore inoculation (procedure, carrier material, drying, etc.)

Figure 1. NO sterilization device is not well described. More details should be given in order to understand how the BIs and bacterial strains are exposed. The illustration is too simplified.

Line 144: “N=3 different BIs were investigated”. Triplicate is ok, but how was the exposure? inside the same container or in 3 different real replicates in separate containers?

Lines 145-146: I don't understand the aim of this test. This is an inhibition test, so are you trying to test inhibition of what? the BI carrier inoculated with the spore suspension? or the possible absorption of NO on the BI carrier?

Line 148: The optimum growth temperature for Geobacillus stearothemophilus is 60ºC, I’m surprised and wondering about the choice of 50ºC. This 10ºC shift could slow down the growth rate, even more in a reduced 3-day incubation (as compared to the appropriate conventional 7 days), written in line 149.

Lines 156-157: “Based on the assumption of Log-linear inactivation kinetics”. Not sure this assumption is correct for chemical processes. Has this been corroborated before this assumption was made for these experiments?

Line 161:

Lines 163-165: More information should be provided regarding the analyzer and internal data. Confidentiality in understood but the concentration of NO is crucial to understand and discuss the sterilization system.

Line 168: “levels outside of this range were marked as Fail.” It would be interesting to see this range with more precision. Thresholds with exact numbers if the safety and robustness of the process is being analyzed.

Line 169: Data and statistical analyses. Information is very scarce. Tables with values for all measures and counts should be given. N should be higher for this process to be approved or conclusions to be more robust and unquestionable.

Line 178: “n=5 for each BI type”. In line 144, N was informed as 3, Is it 3 o 5? Still a low N.

Line 181: “3 most used bacterial endospores”. This is true, but there is bibliography specifically dedicated to demonstrate that these species are used because they have shown to be highly resistant to the sterilization process being monitored. How do we know that this is the same for NO based MO inactivation? Was this assessed? Can you share evidence or data?

Line 205: “the assumption of Log-linear inactivation kinetics”. This assumption is quite risky since chemical processes tend to display non-linear behavior when survivor curves are drawn.

Figure 3: Correlation (R2) of the Bacillus subtilis chart is quite low (0,88) and is actually showing a non-linear model. Also, according to ISO 11138 standard, for D-value calculation at least 20 BIs should be used. Here, even though information is a little confusing and scarce, 5 exposure points with triplicate will give = 15 BIs.

Figure 4: Correlation (R2) of the E. coli chart is quite low (0,81) and is actually showing a non-linear model. Attention should be paid to this behavior for D-value calculation. Too many assumptions are being made and data is not consistent.

In summary:

Experimental design is not well established and/or explained.

Risky assumptions are being made in order to make simplified conclusions.

Statistics is oversimplified and replicates (N) are too low. No raw data is shown.

Other microorganisms and spore species should be assessed in order to make conclusions about the sterilization capacity of the system.

D-value calculation should be re-analyzed because there are calculation assumptions that are not quite safe to be made and chart show that behavior is not linear.

Despite the understandable confidentiality given this new approach, more information should be given to evaluate the robustness and sterilization level achieved by this system.

Reviewer #2: Dear Editor,

I appreciate the opportunity to participate in this review. The manuscript presents a safe and promising alternative for low‑temperature sterilization, although still at an early stage of development. The following points require further clarification by the authors:

Abstract and line 32: Typographical error: “…specifically21-23 °C…”. Please separate the temperature values.

Line 98: The biological indicators tested used paper discs and paper strips as supports. Was any measure taken to evaluate the possible residual effect of the sterilizing agent on the supports? For example, the use of a neutralizing agent? In addition to paper, was another support such as non‑absorbent material considered? I kindly ask the authors to clarify how they discard the hypothesis of residual effect, or whether they consider this a variable to be studied in future experiments.

Line 137: The role of the polypropylene tube was not clearly explained. According to the figure, the tube was placed in the device with a lid. Did the authors perform any complementary sterility tests? For example, inoculation in culture medium?

Lines 140 and 154: Please, provide the references supporting the method used, including any applicable pharmacopeial sources (e.g., USP, EP, or other recognized pharmacopeias/methods).

Line 149: Did the authors conduct any preliminary tests to determine that three days is sufficient? There are publications showing that some bacteria require longer incubation times after being subjected to sterilization conditions. Additionally, some methodologies recommend thermal shock in sterility testing.

Figure 2: Please specify each treatment in the figure description

Best regards,

**********

what does this mean?). If published, this will include your full peer review and any attached files.

If you choose “no”, your identity will remain anonymous but your review may still be made public.

**Do you want your identity to be public for this peer review?**  For information about this choice, including consent withdrawal, please see our Privacy Policy

Reviewer #1: No

Reviewer #2: No

**********

[NOTE: If reviewer comments were submitted as an attachment file, they will be attached to this email and accessible via the submission site. Please log into your account, locate the manuscript record, and check for the action link "View Attachments". If this link does not appear, there are no attachment files.]

To ensure your figures meet our technical requirements, please review our figure guidelines: https://journals.plos.org/plosone/s/figures

You may also use PLOS’s free figure tool, NAAS, to help you prepare publication quality figures: https://journals.plos.org/plosone/s/figures#loc-tools-for-figure-preparation.

NAAS will assess whether your figures meet our technical requirements by comparing each figure against our figure specifications.

# Author comment: Author response to Decision Letter 1

**DOI:** 10.1371/journal.pone.0355735.r002

29 Jun 2026

Dear Editor and Reviewers,

We would like to express our sincere appreciation for your time, effort, and constructive feedback on our manuscript. These comments help us improve both the content and the overall flow of our work. In response to the feedback, we have significantly revised the manuscript. Specifically, we have expanded the text to provide comprehensive information regarding the chemical properties and delivery mechanisms of NO, alongside detailed methodologies for both the sterilization set-up and the bacterial experiments, especially clarification of sampling and adjustment in data presentation. A point-by-point response to each of the specific comments is provided in the Response to Reviewers file.

Sincerely,

Weilue He

Attachment Submitted filename: Response to Reviewers.pdf

# Peer review: Decision Letter 1

**DOI:** 10.1371/journal.pone.0355735.r003

**Authors:** Paulo Laranjeira

26 Jul 2026

Using nitric oxide (NO) for gas sterilization at room temperature: a novel sterilization modality

PONE-D-26-23211R1

Dear Dr. He,

We’re pleased to inform you that your manuscript has been judged scientifically suitable for publication and will be formally accepted for publication once it meets all outstanding technical requirements.

Within one week, you’ll receive an e-mail detailing the required amendments. When these have been addressed, you’ll receive a formal acceptance letter and your manuscript will be scheduled for publication.

An invoice will be generated when your article is formally accepted. Please note, if your institution has a publishing partnership with PLOS and your article meets the relevant criteria, all or part of your publication costs will be covered. Please make sure your user information is up-to-date by logging into Editorial Manager at Editorial Manager® and clicking the ‘Update My Information' link at the top of the page. For questions related to billing, please contact billing support.

If your institution or institutions have a press office, please notify them about your upcoming paper to help maximize its impact. If they’ll be preparing press materials, please inform our press team as soon as possible -- no later than 48 hours after receiving the formal acceptance. Your manuscript will remain under strict press embargo until 2 pm Eastern Time on the date of publication. For more information, please contact onepress@plos.org.

Kind regards,

Paulo Laranjeira

Academic Editor

PLOS One

Additional Editor Comments (optional):

Reviewers' comments:

Reviewer's Responses to Questions

**Comments to the Author**

Reviewer #1: All comments have been addressed

Reviewer #2: All comments have been addressed

**********

2. Is the manuscript technically sound, and do the data support the conclusions?

Reviewer #1: Yes

Reviewer #2: Yes

**********

3. Has the statistical analysis been performed appropriately and rigorously?

Reviewer #1: Yes

Reviewer #2: Yes

**********

4. Have the authors made all data underlying the findings in their manuscript fully available?

The PLOS Data policy

Reviewer #1: Yes

Reviewer #2: Yes

**********

5. Is the manuscript presented in an intelligible fashion and written in standard English?

Reviewer #1: Yes

Reviewer #2: Yes

**********

Reviewer #1: I really appreciate the fact that Authors have addressed all the questions and comments made in the previous review. This paper and the addressed NO technology looks very promising for the future development of a new sterilization technology.

Reviewer #2: The authors have satisfactorily addressed the concerns raised during the previous round of review and have appropriately acknowledged the limitations of their study. Given the preliminary nature of the data, the proposed method appears capable of inactivating the microorganisms. Nevertheless, I would like to reiterate that the principal limitations of the study remain the three-day incubation period and the use of incubation temperatures lower than those recommended in the manufacturer's Instructions for Use (IFU), since the stress imposed by the sterilization process may delay spore germination and, consequently, prolong the time required to detect surviving microorganisms.

**********

what does this mean?). If published, this will include your full peer review and any attached files.

If you choose “no”, your identity will remain anonymous but your review may still be made public.

**Do you want your identity to be public for this peer review?**  For information about this choice, including consent withdrawal, please see our Privacy Policy

Reviewer #1: No

Reviewer #2: No

**********
